# Reconstructing differentially co-expressed gene modules and regulatory networks of soybean cells

**DOI:** 10.1186/1471-2164-13-437

**Published:** 2012-08-31

**Authors:** Mingzhu Zhu, Xin Deng, Trupti Joshi, Dong Xu, Gary Stacey, Jianlin Cheng

**Affiliations:** 1 Department of Computer Science, University of Missouri, Columbia, MO 65211, U.S.A; 2 Informatics Institute, University of Missouri, Columbia, MO 65211, U.S.A; 3 C.S. Bond Life Science Center, University of Missouri, Columbia, MO 65211, U.S.A; 4 Divisions of Plant Sciences and Biochemistry, University of Missouri, Columbia, MO 65211, U.S.A

**Keywords:** Gene co-expression module, Gene regulatory network, Transcription factor, Microarray, Soybean

## Abstract

**Background:**

Current experimental evidence indicates that functionally related genes show coordinated expression in order to perform their cellular functions. In this way, the cell transcriptional machinery can respond optimally to internal or external stimuli. This provides a research opportunity to identify and study co-expressed gene modules whose transcription is controlled by shared gene regulatory networks.

**Results:**

We developed and integrated a set of computational methods of differential gene expression analysis, gene clustering, gene network inference, gene function prediction, and DNA motif identification to automatically identify differentially co-expressed gene modules, reconstruct their regulatory networks, and validate their correctness. We tested the methods using microarray data derived from soybean cells grown under various stress conditions. Our methods were able to identify 42 coherent gene modules within which average gene expression correlation coefficients are greater than 0.8 and reconstruct their putative regulatory networks. A total of 32 modules and their regulatory networks were further validated by the coherence of predicted gene functions and the consistency of putative transcription factor binding motifs. Approximately half of the 32 modules were partially supported by the literature, which demonstrates that the bioinformatic methods used can help elucidate the molecular responses of soybean cells upon various environmental stresses.

**Conclusions:**

The bioinformatics methods and genome-wide data sources for gene expression, clustering, regulation, and function analysis were integrated seamlessly into one modular protocol to systematically analyze and infer modules and networks from only differential expression genes in soybean cells grown under stress conditions. Our approach appears to effectively reduce the complexity of the problem, and is sufficiently robust and accurate to generate a rather complete and detailed view of putative soybean gene transcription logic potentially underlying the responses to the various environmental challenges. The same automated method can also be applied to reconstruct differentially co-expressed gene modules and their regulatory networks from gene expression data of any other transcriptome.

## Background

Genes and proteins in a cell are often organized as a network of interacting modules (e.g. biological pathways) in order to carry out their biological functions. For instance, multiple proteins may form a stable protein complex to regulate gene expression or interact transiently to transduce biological signals. Similarly, a number of genes involved in the same biological process may show coordinated regulation in order to respond effectively to biotic and abiotic stresses. Identifying and characterizing the functional modules (e.g. co-regulated genes and their transcription logic) in a cell would be a natural and necessary approach to studying biological mechanisms underlying various cell activities. Genome-wide profiling of transcriptomes by high-throughput microarray and RNA-sequencing techniques can generate a dynamic, global view of gene expression reflecting gene regulation activities under various biological conditions, which provides necessary information for developing and testing computational modeling methods to decipher transcriptional responses underlying various cellular and molecular processes [1].

As in [2], the whole regulation machinery of a cell can be dissected into a number of transcription regulatory modules. A transcription module is generally comprised of several transcription factors (TFs) and a group of target genes collaboratively or alternatively regulated by the TFs in a combinatorial way. Upon internal or external cellular stimuli, transcription factors of a module may be activated to either up- or down-regulate the target genes in order to respond to the stimuli. The changes in expression levels of target genes and transcription factors captured by microarray techniques can be combined with other genomic data, such as the sequence information and functional annotations of all the genes in order to reversely infer co-regulated genes and their regulators [3]. Accurate prediction of transcription regulatory modules can generate valuable testable hypotheses for designing biological experiments to identify genes and interactions important for biological phenotypes and to elucidate cellular mechanisms underlying various biological conditions and environmental stresses.

Several computational methods have been developed to construct gene regulatory networks or modules from gene expression data [2,4-6]. The Bayesian probabilistic network method [2] can integrate multiple sources of observed information such as gene expression, known transcription factors, and known DNA binding motifs with a probabilistic inference framework to infer co-regulated genes and their putative regulators – transcription factors. The method was successfully applied to the microarray gene expression data of a model species - *Saccharomyces cerevisiae* - measured in multiple biological conditions, identifying a number of highly confident gene regulatory modules. Thus, it is very desirable to develop a general tool that implements and adapts this method to construct gene regulatory modules from gene expression data of any species perturbed by any biological condition, such as plants which have very large, but less well studied genomes and transcriptomes.

Some existing gene regulatory network inference methods require prior biological information about relationships between transcription factors and target genes. Dana *et al*. [5] used mutual information to evaluate relations between a target and its active regulators. This method often limits its search on a set of prior candidate transcription factors, whereas our method (MULTICOM-GNET) can consider all differentially expressed TFs shown in data or even all the known TFs of a species without the need of such prior knowledge. Yao *et al*. [3] developed a maximum likelihood method to prune a rough gene regulatory network based on microarray data. The initial network was constructed from the potential TF-gene regulatory pairs obtained by mining the literature and databases. Thus, the method might not be effectively applied to species with limited gene regulatory knowledge, such as most plants. Joshi *et al*. [7] developed the method for network inference by automatically selecting centroid-like clusters and their TFs. In this method, clustering of genes and assignment of TFs were two separated steps, where our method optimizes the two steps iteratively.

Although a few computational methods were designed to predict transcription regulators and their target genes in *Arabidopsis thaliana*[3,8], the computational prediction of gene regulatory networks for plant species is still at an early stage, partially due to lack of bioinformatic tools or integration methods to combine gene expression data with other data sources to study co-expressed gene modules. Specifically, very little work has been done to construct gene regulatory networks for soybean, an important agricultural crop [9-11], despite the huge amount of gene expression data accumulated for this species during the last several years.

With the availability of the complete genome sequence of the soybean [12] and numerous subsequent annotations of soybean genes and proteins (e.g. SoyDB, a function annotation database of all putative transcription factors [13] and SoyKB, a comprehensive all-inclusive web resource for soybean [14]), it is important and also possible to develop and integrate a set of bioinformatic methods to reliably construct gene regulatory modules by integrating the vast soybean gene expression data with functional genomics data. In this direction, we designed and developed a modular protocol to integrate a set of complementary bioinformatic methods for gene expression data preprocessing, differential expression analysis, gene expression clustering, co-regulated gene module and regulator construction, DNA binding motif identification, and gene function prediction to construct and validate gene regulatory modules. The approach combines both transcriptomic and genomic data to improve gene regulatory network construction. We applied the approach to the gene expression data of soybeans derived from various stress conditions. The analysis produced 32 gene regulatory modules with a high co-expression correlation and function coherence. Approximately half of these modules could be partially validated by the literature. The results demonstrate that our approach can be reliably applied to specific, large-scale expression data of a complicated eukaryotic transcriptome to elucidate the underlying biological mechanisms and processes. The testable gene regulatory networks not only explain the gene expression data and previously known biological evidence but also, perhaps more importantly, can be used to formulate hypotheses that can be tested to generate new biological understanding.

## Methods

### Data

The input data required by our approach includes the soybean genome sequence and gene annotations, a list of the candidate transcription factors (TF) curated in SoyDB [13], and the gene expression profiles calculated from the microarray data of soybean cells from a number of stress-induced experiments [15].

### Microarray gene expression data

The gene expression data used to construct the gene regulatory modules is the publicly available Affymetrix microarray data of soybean cells measured under a number of stress treatments [15]. The RNAs used to generate the data were isolated from multiple soybean tissues, such as leaves and roots. The data include expression measurements of 61,169 gene probes on 99 microarrays. The data was grouped into eight sets corresponding to eight categories of stress treatments, which are 1) iron deficient, 2) *Phytopthora sojae* infected hypocotyl, 3) RNAi storage protein suppression, 4) RNAi oleosion suppression, 5) inoculated with the nitrogen fixing symbiont, *Bradyrhizobium japonicum* 6) inoculated with the fungal pathogen, *Phakopsora pachyrhizi*, 7) Syncytium infected, and 8) infected with the soybean cyst nematode parasite, *Heterodera glycines*. Each set may include several sub-sets of expression data measured with different levels of stress treatment. The total number of treatments for all eight stress categories is 35, each of which may have a few microarray replicates. The average expression value of a gene in multiple microarray replicates was used to represent the expression value of the gene under the treatment.

### Candidate transcription factors

All the 5671 transcription factors (TF) curated in SoyDB [13], which had been automatically classified into 63 TF families by hidden Markov models, were initially selected as candidate gene regulators. The 5480 TFs that actually had expression profiles in the microarray data were used to construct gene regulatory networks.

### Soybean genomics data

We retrieved protein sequences of 46,430 highly confident putative genes from the soybean genome database [12] for gene and protein function prediction, and extracted gene sequences and their 500 upstream DNA sequences from start codon for analysis of DNA binding sites according to the genome locations of the gene probes downloaded from the Affymetrix website (http://www.affymetrix.com).

## Methods

**Figure 1:**
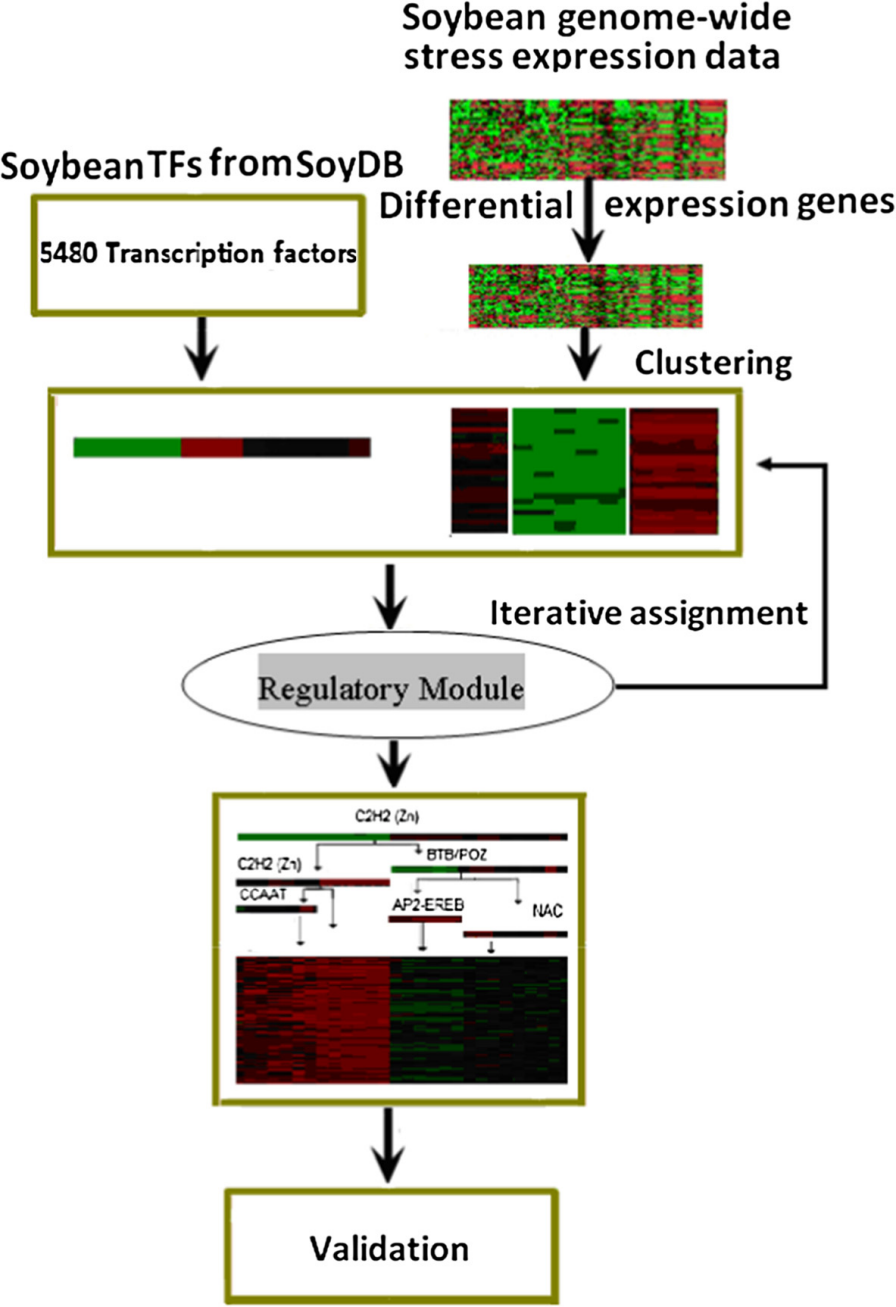
The workflow of the gene regulatory network construction protocol.

The workflow of our gene regulatory network construction protocol is shown in Figure 1. The protocol consists of three main steps: (1) identify differentially expressed genes in control and other treatment conditions; (2) iteratively cluster differentially expressed genes and identify their TF regulators in order to generate modules of co-regulated genes having similar expression patterns in multiple biological conditions and their putative regulators; and (3) validate the gene regulatory modules by checking both the functional consistency of co-regulated genes and the match between conserved binding motifs in upstream regions of the genes and the predicted DNA binding sites of the predicted transcription regulators of the genes. Different from some network construction methods that use all expressed genes as input, our method uses only differentially expressed genes and TFs to construct the regulation networks. This approach may greatly reduce the complexity of network construction by decreasing the number of genes in consideration from almost all the genes in a genome (e.g. >60K) to a much smaller number of differentially expressed genes (e.g. 10K). It may also better address the biological problem under investigation by focusing on the more relevant genes that are most likely activated and deactivated under a particular biological condition. Compared to the use of all expressed genes, our method selects differentially expressed genes that are more specific and relevant in response to experimental conditions, which can increase signal to noise ratio in data analysis. Furthermore, the balance between the specificity and sensitivity of selected genes can be controlled by the threshold of choosing differentially expressed genes. However, one potential limitation of the approach is that some relevant genes and transcription factors that do not have significant expression fluctuation may be missed by the analysis. The problem may be alleviated by incorporating prior knowledge (e.g. known relevant genes) into the automated modeling process. The following sections describe the detailed techniques used in this process.

### Differential gene expression analysis

The microarray data were normalized into gene expression profiles using the RMA algorithm in GeneSpring 10GX [15]. The signal of a probe was normalized by median signal value, i.e., the median of the logarithm expression values of each probe from all samples was subtracted from the logarithm expression value in each sample [15]. Genes with normalized expression values > 3 or < −3 were selected as differentially expressed genes. A total of 10,618 genes (more precisely gene probes), a union of differentially expressed genes in all treatment conditions, were used in gene regulatory network construction. This list included putative transcription factor genes.

### Co-expressed gene clustering and regulatory network construction

As in [5], a regulatory module includes several TFs and a number of genes whose expression is presumably regulated by TFs collaboratively in a series of biological conditions (e.g. stress treatments). It is assumed that the TFs regulate the expression of the genes in a module through the change of their own expression level, which may also be transcriptionally regulated. This assumption is an incomplete simplification of the complex regulatory logic of TFs, because some TFs may not be regulated at the transcriptional level but at a post-translational level, e.g. phosphorylation of TFs. In this work, our method mainly considers the case that the expression profiles of TFs provide information about their activity levels, although other kinds of regulation (e.g., phosphorylation) can be added into our method if data are available. Based on the expression profile – a vector of expression levels under different biological conditions, the expression levels of a TF were clustered into either two or three categories (1: highly expressed, 0: normally expressed, -1: lowly expressed) using the K-means clustering algorithm, where the number of categories equals the number of types (>3, <−3, or between) of the expression situation. The set of transcription factors are assumed to regulate the expression of the genes in a module through a path in the binary decision tree composed of the TFs as internal nodes and condition sub-groups as leaf nodes (Figure 1). A regulatory path from the root node to the leaf node can be interpreted as a series of binary queries on the expression level (up-regulated or not OR down-regulated or not) of internal nodes (i.e., TFs) under treatment conditions leading to the observed expression levels of the genes in the leaf node under the same treatment conditions. Therefore, the regulatory decision tree represents the combinatorial logic by which the TFs regulate the expression of the genes in the module under different treatment conditions.

In order to reduce the complexity of gene regulatory network construction, all the differentially expressed genes were clustered using the K-means algorithm [16], aiming to assign genes exhibiting similar expression patterns across all the treatment conditions into the same cluster. The number of clusters (k) was chosen based on how average correlation coefficients of expression values of genes in the same clusters changed with cluster numbers or the average size of clusters (i.e., number of genes). The knee [17] in the plot of the correlation coefficient versus the average size, which represents the most drastic change of the balance between the two factors, was used to determine the number of clusters. In our experiment, the genes were initially clustered into 100 clusters (see Additional file 1: Figures S1 and S2).

Starting from the initial gene clusters, the gene regulatory modules were constructed in an iterative two-step manner, including (1) constructing a binary tree consisting of several TFs that can best interpret the expression of the genes in a cluster, and (2) re-assigning genes into clusters whose regulatory tree can explain their expression patterns best, i.e., with maximum likelihood. The two steps were alternated until the likelihood of the gene expression data was maximized. Given a gene cluster, a regulatory decision tree was constructed by recursively selecting TFs to divide experimental conditions into two sub-groups such that the expression values of the genes in each group are more coherent. Specifically, the experimental conditions are separated into two sub-groups according to the expression level of a TF in the conditions, i.e., the conditions where a TF was highly expressed (resp. lowly expressed) were assigned to one sub-group and others to the other sub-group. Assuming the expression values in each sub-group of conditions obey the normal distribution, the probability of the expression value (*x*) of a gene *i* (g_i_) is calculated as $\frac{1}{\sqrt{2\pi \varsigma }}e^{\frac{{\left(x-\mu \right)}^{2}}{2\varsigma^{2}}}$ , where *μ* is the mean expression value in the sub-group, *σ* the standard deviation of expression values in the sub-group, and *x* the expression value of the gene g_i_ in a condition assigned to the sub-group. The product of the probabilities of the expression values of every gene under every condition is considered the probability that the TF regulates this group of conditions. The product was further transformed by the logarithm function into a likelihood score. The TF that divided the group of conditions with the highest likelihood score was selected. The sub-group of conditions can be further divided by another TF in the same fashion until the gene expression values grouped together are similar enough or the maximum level of partitions had been reached. The TFs were selected to divide conditions from the nodes of the regulatory tree, where each node has two branches corresponding to its two expression states: highly expressed (resp. lowly expressed) or not-highly-expressed (resp. not-lowly-regulated). A branch connects one TF node (parent node) in the upper level to another TF node (child node) in the lower level that was selected to divide a sub-group generated by the parent TF node. The first TF selected to divide the whole group of genes was the root of the tree. The TFs that do not have TF children nodes directly connect to the sub-groups of conditions divided by them. Generally a regulatory decision tree has one to five levels of TF nodes. A path from the root node to a leaf node forms a regulatory logic, i.e., a list of combinatorial states of TF nodes on the path, which regulates the expression of genes in a sub set of conditions represented by the leaf node.

After a gene regulatory tree was constructed for every gene cluster, a gene re-assignment procedure was used to assign each gene to a cluster whose regulatory tree best explained its expression values in all the treatment conditions as follows:

Assuming that a regulatory tree divides experimental conditions into a set of sub-groups $-S=\left\{S_{1},S_{2},S3\right\}$and the mean and standard deviation of the gene expression values in a sub-group S_k_ were μ_k_ and σ_k_, respectively, according to the normal distribution, the probability (likelihood) of the expression values of a gene g_i_ under all treatment conditions was calculated as:

$p\left(g_{i}\right)=\prod_{k-1}^{s}\prod_{jes}\frac{1}{\sqrt{2\pi \sigma_{k}}}e^{-\frac{\left(x-\mu \right)}{2\sigma }}$where *x*_*ij*_ was the expression value of g_i_ under condition *j*. This calculation of likelihood was based on the simplified assumption that normalized expression values of a gene under different conditions were independent, which was a largely reasonable approximation if gene expression experiments were carried out independently. However, the approximation did not account for the co-variation between expression values. The log-likelihood of gene g_i_ was $l\left(g_{i}\right)∼\overset{m}{\underset{k-1}{\Sigma }}\sum_{jes}-\frac{{\left(x_{\text{ij}}-\mu_{k}\right)}^{2}}{2\sigma_{k}^{2}}-1n\left(\sigma_{k}\right)$. After the log-likelihood scores of g_i_ were calculated with respect to all regulatory trees, g_i_ was assigned to the regulatory tree yielding the highest likelihood score. The genes assigned to the same regulatory tree formed a cluster. In this way, all the genes were clustered into a new set of clusters. The log-likelihood of a gene cluster $M=\left\{g_{1},g_{2},g_{n}\right\}$ can be represented as: $l\left(M\right)∼\overset{n}{\underset{i-1}{\Sigma }}\overset{m}{\underset{k-1}{\Sigma }}\underset{jes}{\Sigma }-\frac{{\left(x_{\text{ij}}-\mu_{k}\right)}^{2}}{2\sigma_{k}^{2}}-1n\left(\sigma_{k}\right)$. For each group, a regulatory tree was constructed according to the same protocol described above. The regulatory tree construction step and the gene re-assignment step were iterated until the assignment of genes did not change. When the protocol stopped, the final clusters of genes and their regulatory trees formed a set of predicted gene regulatory modules.

### Function prediction

We used MULTICOM [18,19], a protein structure and function prediction software, to predict the functions of the differentially expressed genes in order to study the function coherence of genes in regulatory modules. MULTICOM tried to predict three categories of functions (i.e. biological processes (BP), molecular function (MF), and cellular component (CC)) in terms of the Gene Ontology (GO) definition [20] for each differentially expressed gene. The predictions were presented as both GO terms and human readable descriptions.

### Statistical consistency analysis of modules

We evaluated the coherence of each regulatory module from two aspects: 1) Pearson correlation coefficient of expression values of genes in the module; and 2) GO gene function enrichment. According to gene expression values, we calculated the Pearson correlation coefficient of every two genes of the module, and then we averaged all the pairwise correlation coefficients within the module as the correlation coefficient of the module.

### Gene function enrichment analysis

To analyze the biological relevance of each module, we studied the functional consistency of genes assigned to the same module.The predicted GO function terms of the genes were compared according to the hypergeometric distribution [21,22] in order to check if some biological process terms or molecular function terms were significantly more enriched than by chance. If one gene probe on the microarray chip corresponded to more than one gene (e.g. multiple isoforms of a gene), all the corresponding genes were considered for functional analysis.

### DNA binding site analysis

As in [2], we used a DNA binding site analysis to further validate if the predicted TFs of a module likely regulated the genes in the module. The locus information corresponding to each probe was downloaded from http://www.affymetrix.com. We extracted the upstream 500 bp sequences of genes within every predicted gene module. We used MEME [23] to analyze 500 bp upstream sequences of the genes in each module to identify significantly conserved sequence motifs consisting of 6 to 18 nucleotides, which were considered potential sites for TFs to bind. The sites with p-value < 0.001 were selected as putative motifs. The putative motifs were compared with known TF binding motifs in a transcription factor database JASPAR [24] by TomTom [25]. TomTom ranked the motifs in the target database according to their similarity with the putative motifs of the genes. The annotated transcription factors of the motifs in the JASPAR database that were significantly similar to the putative motifs of the genes in the module were examined against the predicted regulators of the module. If they shared similar function or belonged to the same TF family, the predicted regulators were considered more likely to regulate the genes in the module.

## Results

### The overall analysis of all differentially expressed genes

**Figure 2:**
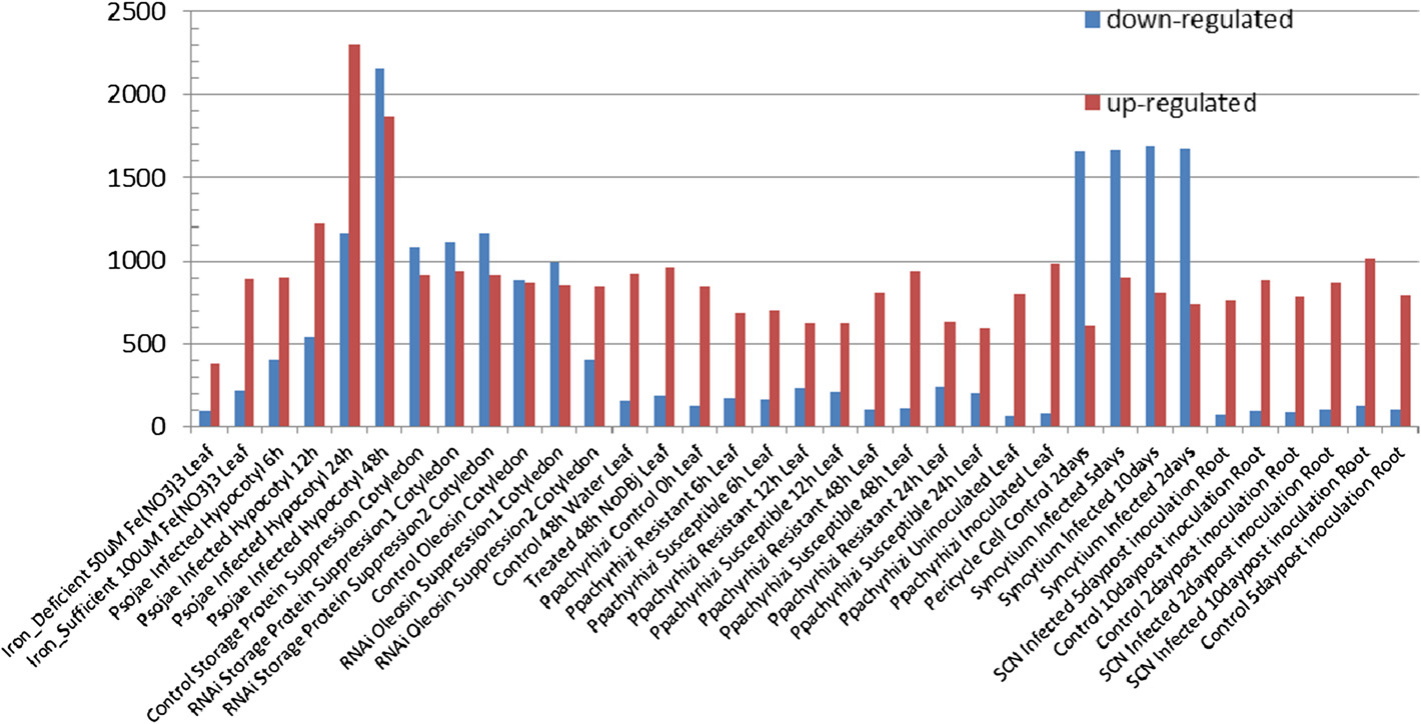
Number of differentially expressed genes in 35 conditions.

**Figure 3:**
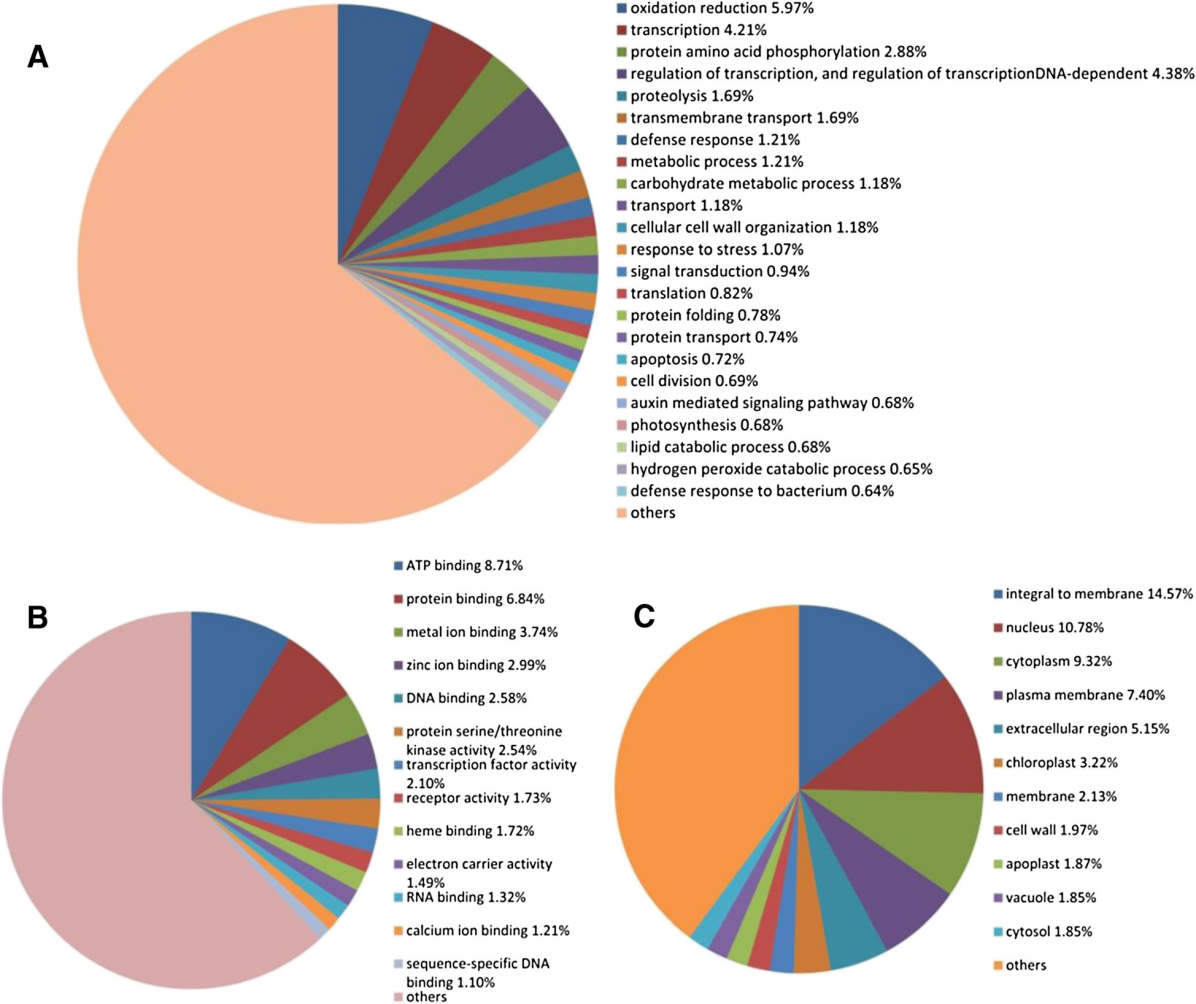
Function distribution of differentially expressed genes in terms of biological process (A), molecular function (B), and cellular components (C).

Figure 2 reports the number of differentially expressed (up- and down-regulated) genes (DEG) in each of 35 specific experimental conditions. It shows that the numbers of up- and down-regulated genes are often very different within a condition and between conditions. Figure 3 depicts the distribution of predicted functions of all the differentially expressed genes in terms of biological processes, molecular function, and cellular components (Figure 3). Figure 3(A) shows that a large portion of genes involved in oxidation reduction, transcription, and phosphorylation, indicating that oxidation reduction pathways, gene regulatory pathways, and signal transduction pathways may be perturbed by the stress conditions. According to Figure 3(B), many differentially expressed genes participated in various binding activities, including ATP binding, protein binding, metal ion binding, zinc ion binding, DNA binding, RNA binding and heme binding, in response to the stresses.

### A large-scale analysis of predicted gene regulatory modules

**Figure 4:**
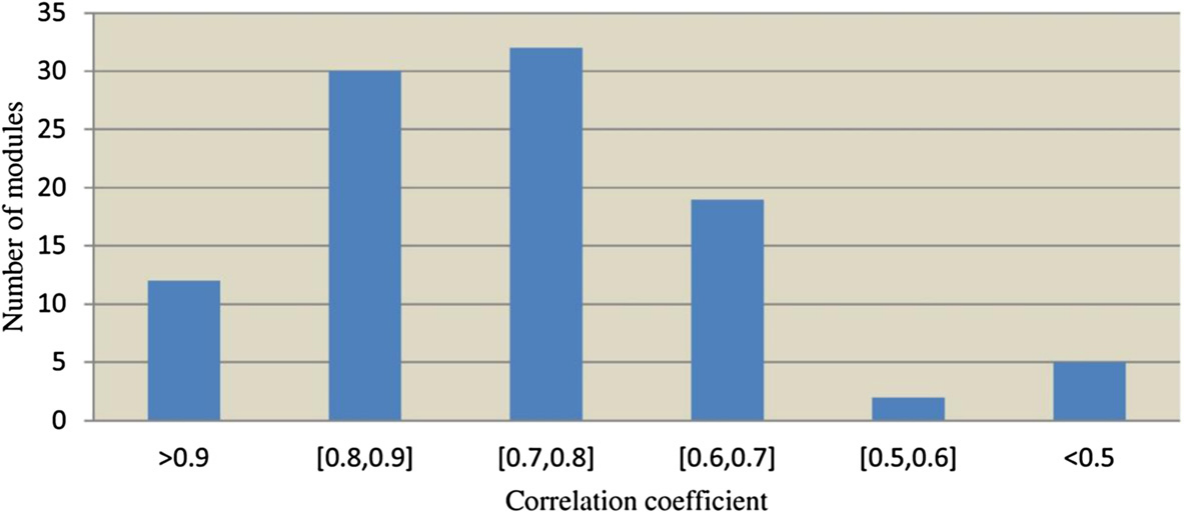
The histogram of the Pearson correlation coefficients of 100 predicted gene modules.

All the differentially expressed genes were assigned to 100 gene regulatory modules. Figure 4 is the histogram of Pearson’s correlation coefficients of modules of co-expressed genes. As shown, 93 gene modules have a correlation coefficient value greater than 0.6 (the details of these modules were reported in Part A and B in the Additional file 1), while 42 gene modules have a correlation coefficient value greater than 0.8 (Additional file 1: Table S1 in Part A in the supplemental document), in which some gene functions appear to be significantly enriched.

We focused on assessing the validity of 42 gene regulatory modules whose correlation coefficients were greater than 0.8 from four aspects: (1) functional enrichment of genes in a module, (2) interaction potential between TFs predicted by STRING [26], (3) the goodness of fit between the motifs extracted from upstream of genes in a module and the annotated target motifs of the TFs predicted to regulate the module, and (4) literature confirmation of the regulatory function of TFs and the genes in corresponding experimental conditions. A final list of 32 modules had some and all supporting evidence using these four criteria (Table 1). The results show that a variety of biological processes may be activated or deactivated under different stress treatments, in which the expression of response genes seemed to be highly coordinated. The validity of most modules was furthered confirmed by the potential interactions among the predicted TFs within these modules predicted by STRING or by the possible match between the DNA binding motifs of the putative TFs and the conserved motifs in the upstream sequences of the genes in the modules. Perhaps of greater importance, the relationships between the putative TFs / the stress treatments and the gene function of 16 modules were supported by published work according to our literature search (Table 1). 

**Table 1:** 32 Gene regulatory modules with high correlation coefficients and other supports

**Correlation coefficient**	Most enriched biological process*	**TF**	**Gene**	**Coherence**^**†**^	**I**^**$**^	**M**^**&**^	**L**^**#**^
0.989913	lipid transport	7	62	6	Ce	√	√ [27]
0.945372	positive regulation of release of sequestered calcium ion into cytosol	7	11	36			√ [36]
0.930583	vegetative to reproductive phase transition of meristem	8	161	3	Ce	√	√ [37,38]
0.926575	proteolysis	8	27	18	E	√	√ [39]
0.910277	steroid biosynthetic process	7	81	3		√	√ [40]
0.902439	photosynthesis	9	114	12	T		√ [41]
0.898638	protein transport	9	183	8	Ce	√	
0.896121	regulation of cell shape	8	50	6	E&H		
0.893346	ATP-dependent chromatin remodeling	6	163	1	E	√	
0.890829	photosynthesis, light harvesting	11	102	12		√	√ [41]
0.888479	mRNA processing	9	218	7	Ce	√	
0.882899	translational elongation	9	85	4	E	√	
0.880672	regulation of flower development	9	57	7	Ce	√	√ [42]
0.867582	lipid transport	7	63	6	Ce	√	
0.862264	skeletal system morphogenesis	9	70	4	T	√	
0.857502	translation	11	125	12	Ce		
0.855305	carotenoid biosynthetic process	9	103	3	Ce	√	
0.852366	lactate metabolic process	7	84	4	E,T&H	√	
0.843827	establishment or maintenance of polarity of embryonic epithelium	5	99	6	E	√	
0.841523	flavonoid biosynthetic process	8	44	6	E	√	√ [29]
0.836985	base-excision repair	10	36	8	E		
0.830088	flavonoid biosynthetic process	9	38	15	E&T	√	√ [29]
0.826087	acetyl-CoA biosynthetic process	7	158	3	E		√ [43]
0.824363	auxin mediated signaling pathway	12	76	5	E	√	√ [44]
0.820377	carbohydrate metabolic process	9	71	11	T		√ [45]
0.817311	transcription	9	56	25	E	√	
0.81347	superoxide metabolic process	10	46	6	T		
0.812358	nodulation	10	73	4	E	√	√ [28]
0.809849	PSII associated light-harvesting complex II catabolic process	8	108	2	E&Ce	√	√ [45]
0.808305	metabolic process	7	44	13	T	√	
0.806202	secondary cell wall biogenesis	8	97	6	E	√	√ [46]
0.804089	DNA replication initiation	10	96	4	Ce	√	

Column 1 lists the average of the pairwise Pearson’s correlation coefficients of expression values of genes in these modules. Column 2* reports the most significantly enriched GO biological process in each module that has the smallest p-value. Column 3 lists the number of TFs predicted for a module. Column 4 lists the number of genes in a module that had GO annotations. Column 5^†^ lists the percent of genes in each module participating in the biological process. Column 6 (I^$^) shows if at least two predicted TFs in a module interacted according to the STRING predictions and the BLAST homology search. Evidence sources for STRING predictions include E - experiments; Ce - co-expression; T – text mining; H - homology. Column 7 (M^&^) reports if the DNA binding motifs of some predicted TF families in a module matched the motifs extracted from the upstream sequences of the regulated genes in the module. Column 8 (L^#^) lists if previous literature had reported a relationship amongst at least one predicted TF family, a stress condition, and the predicted gene function of a module.

### A detailed case study of some gene regulatory modules

**Figure 5:**
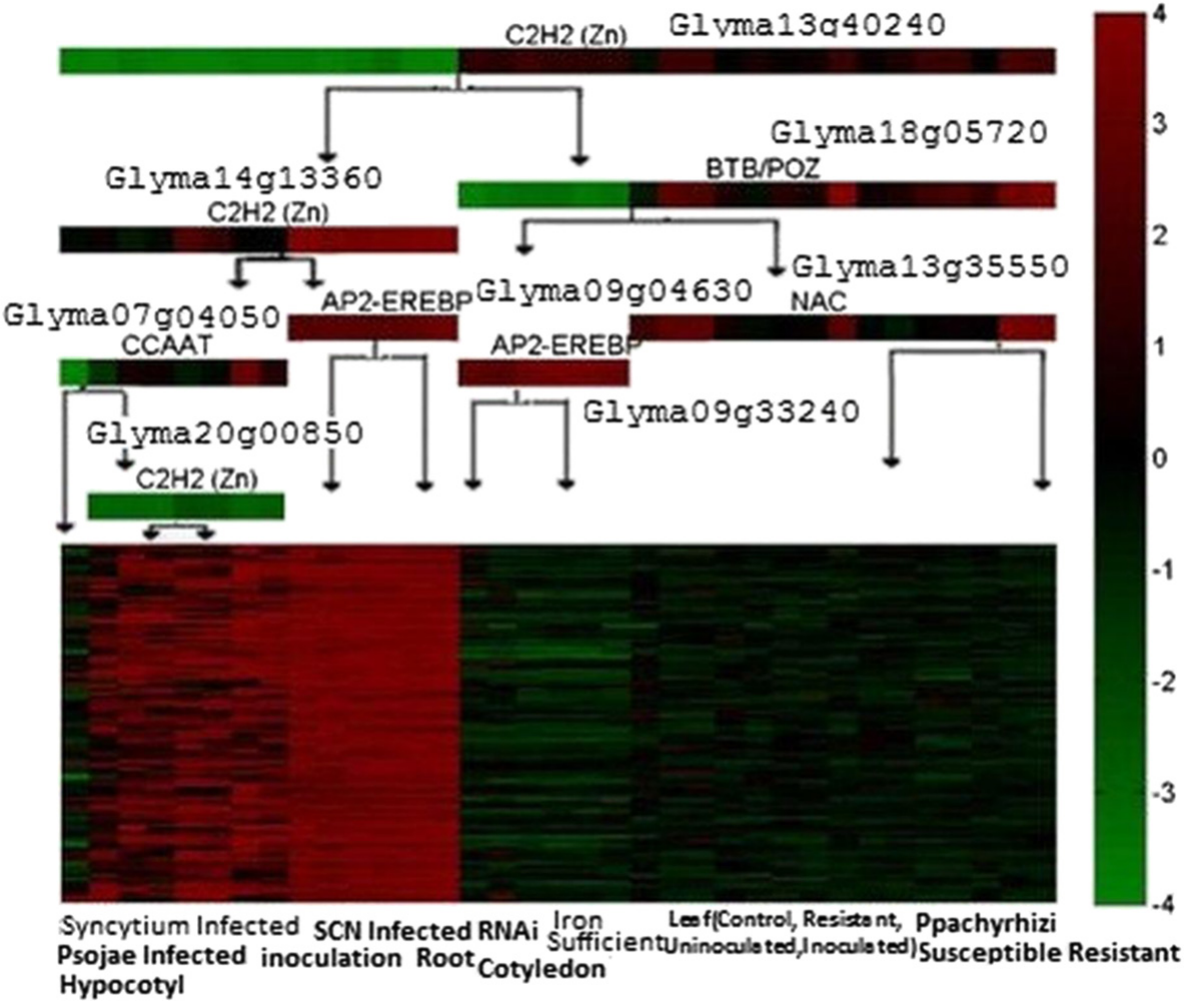
**Gene regulatory module 1.** The average expression coefficient of this module is 0.8124. The module contains 95 genes, 73 of which have predicted GO functions. The tree-like upper part visualizes the predicted regulatory decision tree of the module, where a tree node (i.e. color bar) represents a query on the expression level of a predicted TF and a branch (i.e., arrowed edge) denotes a yes / no answer (highly / lowly expressed or not) to the query. The family names and gene IDs of the predicted TFs are listed above the nodes. A path from the top to a sub-group of conditions shown in the square box at the bottom predicts that the TFs on the path collaboratively regulate the expression of the genes in these conditions. For example, the right most path suggests that, if C2H2 is highly expressed, BTB/POZ highly expressed, and NAC highly expressed, the genes in the modules will be down regulated in the *P. pachyrhizi* resistant condition. The colored square box visualizes the expression levels of all genes in the module across all the conditions, where a row denotes a gene and a column denotes a stress condition. The color bar on the right illustrates a specific color with an expression value, from the lowest (green) to the highest (red).

By way of example, we chose to present two gene regulatory modules in greater detail. The information of all other modules can be found in the supplemental document. Figure 5 depicts the first module with the average correlation coefficient 0.8124, which includes 95 gene probes (more precisely gene probes on the microarray chip). These genes were predicted to be regulated by 8 TFs in a combinatorial manner. For instance, if C2H2 (ZN) (Glyma13g40240) – a member of DNA and chromatin-binding TF family - is lowly expressed, C2H2 (ZN) (Glyma14g13360) highly expressed, and AP2/EREBP (Glyma09g04630) highly expressed, the genes in the module will be up-regulated. This is consistent with the report of Riechmann, *et al*. [27] showing that AP2/ EREBP genes are involved in the response to various types of biotic and abiotic stress, as well as the report of TAIR [28] that about a half of the AP2-EREBP family transcription factors are ethylene responsive element binding factors. Furthermore, according to the enrichment analysis on the predicted functions of 73 genes whose functions could be predicted by MULTICOM, the enriched biological processes of the module included the ethylene-mediated signaling pathway, transcription regulation, cellular cell wall organization, etc., and the enriched molecular functions included sequence-specific DNA binding, transcription factor activity, and protein dimerization (Table 2), which are consistent with the functions of the TF families. 

**Table 2:** Enriched gene ontology functions in gene regulatory module 1

	**Enriched GO function**	**Number of genes**	**P-value**
GO:0006350	P:transcription	11	3.57E-03
GO:0006355	P:regulation of transcription, DNA-dependent	9	1.60E-04
GO:0007047	P:cellular cell wall organization	6	9.88E-04
GO:0009873	P:ethylene mediated signaling pathway	3	1.00E-02
GO:0008360	P:regulation of cell shape	3	6.06E-04
GO:0009877	P:nodulation	3	1.44E-04
GO:0042744	P:hydrogen peroxide catabolic process	3	2.00E-02
GO:0009252	P:peptidoglycan biosynthetic process	3	4.31E-04
GO:0043565	F:sequence-specific DNA binding	6	8.73E-04
GO:0003700	F:transcription factor activity	8	8.96E-04
GO:0030528	F:transcription regulator activity	3	1.00E-02
GO:0046983	F:protein dimerization activity	3	2.00E-02
GO:0004601	F:peroxidase activity	3	2.00E-02
GO:0003680	F:AT DNA binding	3	2.37E-05
GO:0003690	F:double-stranded DNA binding	3	3.76E-05

Columns 1 and 2 list the GO terms and the names of the enriched GO biological processes and molecular functions, respectively. P - biological process, F - molecular function. Columns 3 and 4 report the number of genes with the predicted function and the corresponding p-value of enrichment. The p-values were calculated according to a hypergeometric distribution as in [6].

**Figure 6:**
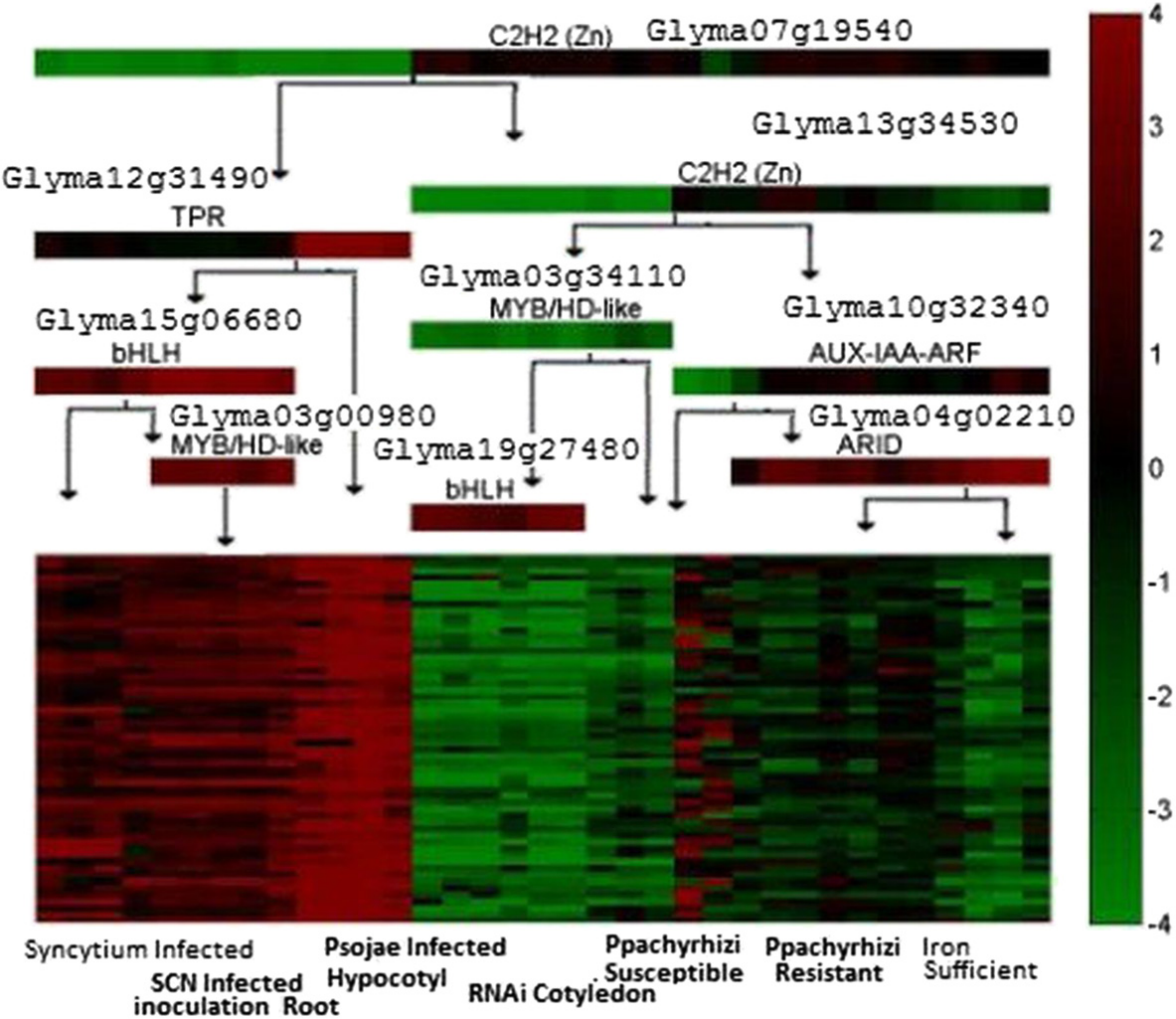
The gene regulatory module 2.

The second module (Figure 6) has 56 genes (more precisely gene probes on the microarray chip) whose average correlation coefficient is 0.8301. MULTICOM was able to predict GO functions for 38 genes (Table 3). According to the enrichment analysis, several biological processes such as flavonoid biosynthetic process and defense response were significantly enriched. This is consistent with the previous research on the transcriptional regulation of the flavonoid biosynthetic pathway [29] that the structural gene expression in plant development was orchestrated by a ternary complex involving TF families R2R3-MYB, basic helix–loop–helix (bHLH), and WD40, which regulated the genes of the flavonoid biosynthetic pathway leading to the biosynthesis of anthocyanins and condensed tannins [29]. Interestingly, the TFs in the bHLH and MYB families were also predicted by our method to regulate the genes in this module (Figure 6). Furthermore, we used Blast2GO [30] to map the predicted genes in the module to the pathways in KEGG [31], and found 4 of them (Glyma19g32650, Glyma03g29950, Glyma19g32880 and Glyma06g03860) could be mapped to flavonoid biosynthesis pathway in KEGG. 

**Table 3:** Enriched functional terms of gene regulatory module 2

	**GO function name**	**Annotation number**	**Hypergeometric P-value**
GO:0009813	P:flavonoid biosynthetic process	6	5.94E-07
GO:0006952	P:defense response	4	3.96E-03
GO:0009607	P:response to biotic stimulus	3	1.03E-03
GO:0042744	P:hydrogen peroxide catabolic process	4	5.19E-04
GO:0055114	P:oxidation reduction	19	1.99E-10
GO:0009055	F:electron carrier activity	6	1.17E-04
GO:0020037	F:heme binding	9	2.58E-07
GO:0004497	F:monooxygenase activity	3	2.03E-03
GO:0004601	F:peroxidase activity	5	3.64E-05
GO:0050662	F:coenzyme binding	5	1.71E-06
GO:0045552	F:dihydrokaempferol 4-reductase activity	5	2.01E-08
GO:0047890	F:flavanone 4-reductase activity	5	1.53E-09

In order to assess the robustness of our module construction process against thresholds of selecting DEGs, we compared the two modules above with the corresponding modules constructed from DEGs with absolute normalized expression values > 4 and 5, respectively. Based on threshold 4, 49 of 95 genes (more precisely gene probes on the microarray chip) in the gene regulatory module (based on threshold 3 above) were retained, and 41 out of 49 genes were grouped into the same cluster. Three TFs predicted for the cluster based on threshold 3 were the same as those in module 1 based on threshold 3. If the threshold were increased to 5, only 8 of 95 genes would be kept, which was probably too small to form a cluster in this case. As for the gene regulatory module 2, based on threshold 5, 47 out of 56 genes were selected, and 34 of them were grouped into the same cluster. Furthermore, 29 out of 34 genes in the cluster overlapped with the cluster of module 2. And interestingly, all 9 TFs predicted for the cluster were exactly the same as those in module 2. This analysis demonstrates that our method can produce rather consistent results with a reasonable range of thresholds of selecting differentially expressed genes. The threshold may be decided based on both the stringency of statistics significance and how muchbiological information is needed.

### DNA binding site validation

In addition to functional analysis, we studied the potential DNA binding sites of the genes and the transcription factors in the two modules. Table 4 shows the most significant DNA motifs extracted from the upstream sequences of the genes in the two modules. The two TF motifs - AZF1 and YGR067C – predicted to bind to the DNA binding sites by TomTom are both zinc finger domains in the BetaBetaAlpha-zinc finger family. The TF family contains the C2H2(ZN) subfamily [32] that was predicted to be a regulator of the two modules by our method. Furthermore, previous studies [33-35] reported that AZF1 regulated a set of genes that maintained cell wall integrity, which was consistent with the cellular cell wall organization function predicted for six genes in module 1 (see Table 2). 

**Table 4:** Binding site predictions for module 1 and module 2

**Module**	**Consensus sequence**	**Motif logo**	**P-value**	**TF motif**	**TF family**
1	AAAAAGAAA		1.68e-05	AZF1	BetaBetaAlpha – Zinc Finger
2	ACCCCACT		3.74e-04	YGR067C	BetaBetaAlpha – Zinc Finger

Column 1 lists the putative consensus DNA binding motifs. Column 2 is the motif logo visualizing the information content of nucleotides (i.e. information content) at each position of the DNA motifs. Column 3 shows the p-value of the motifs. Column 4 is the predicted TF motifs that may bind to the DNA motifs. Column 5 is the family name of the TFs that may bind to the DNA motifs. The TF motifs were predicted by TomTom from the DNA binding motifs.

## Discussion

In this work, we developed and applied a series of computational methods to construct the gene regulation networks involved in soybean’s responses to a number of stress conditions. The soybean is a good choice for this demonstration since, although of major agronomic importance, this plant has not been as extensively studied as other model species, such as yeast, mouse, human and Arabidopsis. The networks consisted of a list of gene regulation modules that included both a set of genes expressed similarly under the various stress conditions and several putative TF regulators. The regulatory networks were reconstructed from gene expression data in conjunction with other data sources such as genomics data and protein function data. In the same computational framework, a large number of predicted gene regulatory modules were validated by gene expression coherence, function enrichment analysis, TF-gene binding potentials, and the literature [27-29,36-46]. The results demonstrate that the approach can infer detailed and testable gene regulatory modules that link TFs, regulated genes, and biological conditions together, which can be used to design targeted biological experiments, such as gene knock out, chip-Seq DNA binding analysis, protein-protein interaction test, and RNA interference of TFs. Particularly, predictive hypotheses may be used to validate predicted TFs that have not yet been confirmed.

In addition to the capability of integrating multiple data sources, applicable to gene expression data of any species, and considering both spatial and temporal information in different tissues and multiple replicates, our approach is unique in focusing on differentially expressed genes in the process of gene regulatory network construction, which appears to reduce its complexity and increase its biological relevance. In the future, we plan to integrate more data sources such as protein-protein interaction, protein phosphorylation, proteomics, and miRNA data to improve the accuracy of gene regulatory network construction. We also aim to elucidate the relationships between gene regulatory modules through shared genes and TFs, and to construct metabolic and signal transduction pathways involving genes in the same regulatory modules.

## Conclusion

In this work, we developed and applied a modular bioinformatics procedure to automatically construct gene regulatory networks for any species by integrating microarray gene expression data with other data sources. We benchmarked the method on the gene expression data of soybean. It effectively predicted a number of partially validated gene regulatory modules. The experiment demonstrates that the bioinformatics approach can be used to automatically predict gene regulatory networks from large-scale transcriptomic and genomic data for a species with large genome and transcriptome under specific biological conditions. The predicted networks can be used to generate biological hypotheses for experimental design and validation.

## Competing interests

The authors declare that they have no competing interests.

## Authors’ contributions

JC conceived the project. JC and MZ designed the method and experiment. MZ implemented the methods and carried out the experiment. MZ and JC analyzed the data. XD contributed to DNA binding site analyses. TJ and DX contributed to microarray data preprocessing. MZ and JC wrote the manuscript. GS contributed to the generation of some of the datasets and aided in providing a biological context for the results. All of the authors edited and approved the manuscript.

## Supplementary Material

Additional file 1 **A supplemental document.** The supplemental document includes three parts. Part A: all the modules with correlation coefficient greater than 0.600. Part B: the list of numbers used to represent different experimental conditions in gene regulatory network figures shown in Part C. Part C: the detailed information of the modules with correlation coefficient >= 0.800, including a module ID, a figure visualizing gene regulatory network and gene cluster, the list of enriched Gene Ontology biological processes and the p-values, the IDs and families of predicted transcription factors, and the IDs of genes in the module. Part D: Figures 1 and 2 illustrating how to determine the number of clusters. Click here for file
