# Supplementary material for: Reconstructing differentially co-expressed gene modules and regulatory networks of soybean cells
Source: BMC Genomics. 2012 Aug 31;13:437. doi: 10.1186/1471-2164-13-437 (PMC3563468; doi:10.1186/1471-2164-13-437)
Supplement: Additional file 1 — A supplemental document. The supplemental document includes three parts. Part A: all the modules with correlation coefficient greater than 0.600. Part B: the list of numbers used to represent different experimental conditions in gene regulatory network figures shown in Part C. Part C: the detailed information of the modules with correlation coefficient >= 0.800, including a module ID, a figure visualizing gene regulatory network and gene cluster, the list of enriched Gene Ontology biological processes and the p-values, the IDs and families of predicted transcription factors, and the IDs of genes in the module. Part D: Figures 1 and 2 illustrating how to determine the number of clusters. [file 1471-2164-13-437-S1.pdf]

# Supplemental Materials

# Part A

**Table S1:** All the modules with correlation coefficient greater than 0.600.

| Module ID | GO ID | GO_NAME | N1 | N 2 | N 3 | Ratio 1 | Ratio 2 | P value |
|---|---|---|---|---|---|---|---|---|
| 7 | GO:0009408 | P:response to heat | 74 | 3 | 40 | 0.54 | 0.07 | 0.00248 |
| 57 | GO:0009399 | P:nitrogen fixation | 103 | 3 | 65 | 0.63 | 0.04 | 3.02E-05 |
| 15 | GO:0042128 | P:nitrate assimilation | 93 | 3 | 59 | 0.63 | 0.05 | 7.47E-05 |
| 99 | GO:0009409 | P:response to cold | 72 | 3 | 43 | 0.59 | 0.06 | 0.00416 |
| 85 | GO:0055114 | P:oxidation reduction | 85 | 20 | 67 | 0.78 | 0.29 | 7.54E-08 |
| 49 | GO:0055114 | P:oxidation reduction | 96 | 25 | 76 | 0.79 | 0.32 | 1.79E-10 |
| 29 | GO:0046373 | P:L-arabinose metabolic process | 87 | 4 | 64 | 0.73 | 0.06 | 6.23E-08 |
| 81 | GO:0005975 | P:carbohydrate metabolic process | 116 | 7 | 85 | 0.73 | 0.08 | 0.000541 |
| 62 | GO:0006821 | P:chloride transport | 107 | 3 | 68 | 0.63 | 0.04 | 9.81E-06 |
| 65 | GO:0019318 | P:hexose metabolic process | 123 | 4 | 83 | 0.67 | 0.04 | 1.72E-08 |
| 55 | GO:0030163 | P:protein catabolic process | 88 | 3 | 56 | 0.63 | 0.05 | 0.000756 |
| 75 | GO:0009944 | P:polarity specification of adaxial/abaxial axis | 67 | 3 | 44 | 0.65 | 0.06 | 1.31E-05 |
| 64 | GO:0006695 | P:cholesterol biosynthetic process | 97 | 4 | 78 | 0.8 | 0.05 | 3.25E-08 |
| 82 | GO:0006915 | P:apoptosis | 54 | 10 | 43 | 0.79 | 0.23 | 0 |
| 82 | GO:0009626 | P:plant-type hypersensitive response | 54 | 10 | 43 | 0.79 | 0.23 | 0 |
| 46 | GO:0050826 | P:response to freezing | 76 | 3 | 46 | 0.6 | 0.06 | 1.21E-05 |
| 12 | GO:0009873 | P:ethylene mediated signaling pathway | 61 | 4 | 36 | 0.59 | 0.11 | 0.000186 |
| 17 | GO:0051865 | P:protein autoubiquitination | 137 | 3 | 90 | 0.65 | 0.03 | 2.1E-06 |
| 23 | GO:0006468 | P:protein amino acid phosphorylation | 118 | 15 | 71 | 0.6 | 0.21 | 1.13E-06 |
| 87 | GO:0006355 | P:regulation of transcription, DNA-dependent | 74 | 11 | 58 | 0.78 | 0.18 | 3.43E-07 |

| | | | | | | | | |
|---|---|---|---|---|---|---|---|---|
| 87 | GO:0009631 | P:cold acclimation | 74 | 3 | 58 | 0.78 | 0.05 | 3.77E-05 |
| 100 | GO:0044403 | P:symbiosis, encompassing mutualism through parasitism | 83 | 4 | 65 | 0.78 | 0.06 | 5.15E-08 |
| 61 | GO:0016042 | P:lipid catabolic process | 128 | 17 | 95 | 0.74 | 0.17 | 4.08E-11 |
| 27 | GO:0009607 | P:response to biotic stimulus | 73 | 6 | 44 | 0.6 | 0.13 | 1.99E-07 |
| 6 | GO:0009809 | P:lignin biosynthetic process | 101 | 6 | 63 | 0.62 | 0.09 | 1.88E-06 |
| 28 | GO:0009867 | P:jasmonic acid mediated signaling pathway | 107 | 4 | 65 | 0.6 | 0.06 | 3.05E-06 |
| 13 | GO:0006869 | P:lipid transport | 61 | 9 | 56 | 0.91 | 0.16 | 3.05E-11 |
| 35 | GO:0009734 | P:auxin mediated signaling pathway | 107 | 5 | 59 | 0.55 | 0.08 | 0.000775 |
| 96 | GO:0009607 | P:response to biotic stimulus | 71 | 5 | 41 | 0.57 | 0.12 | 4.9E-06 |
| 44 | GO:0046274 | P:lignin catabolic process | 119 | 6 | 97 | 0.81 | 0.06 | 1.51E-07 |
| 5 | GO:0006526 | P:arginine biosynthetic process | 148 | 3 | 120 | 0.81 | 0.02 | 0.00021 |
| 32 | GO:0010224 | P:response to UV-B | 96 | 4 | 76 | 0.79 | 0.05 | 4.87E-05 |
| 2 | GO:0009809 | P:lignin biosynthetic process | 66 | 6 | 50 | 0.75 | 0.12 | 1.49E-07 |
| 97 | GO:0009809 | P:lignin biosynthetic process | 111 | 4 | 64 | 0.57 | 0.06 | 0.000774 |
| 31 | GO:0006355 | P:regulation of transcription, DNA-dependent | 88 | 7 | 67 | 0.76 | 0.1 | 0.0024 |
| 73 | GO:0055114 | P:oxidation reduction | 98 | 22 | 71 | 0.72 | 0.3 | 4.2E-08 |
| 18 | GO:0006206 | P:pyrimidine base metabolic process | 84 | 3 | 46 | 0.54 | 0.06 | 3.88E-05 |
| 88 | GO:0000272 | P:polysaccharide catabolic process | 73 | 3 | 36 | 0.49 | 0.08 | 2.54E-05 |
| 72 | GO:0015074 | P:DNA integration | 66 | 7 | 49 | 0.74 | 0.14 | 0 |
| 72 | GO:0006278 | P:RNA-dependent DNA replication | 66 | 8 | 49 | 0.74 | 0.16 | 0 |
| 10 | GO:0007047 | P:cellular cell wall organization | 55 | 5 | 30 | 0.54 | 0.16 | 0.000505 |
| 10 | GO:0009607 | P:response to biotic stimulus | 55 | 3 | 30 | 0.54 | 0.1 | 0.000976 |
| 60 | GO:0006096 | P:glycolysis | 61 | 3 | 32 | 0.52 | 0.09 | 0.00445 |
| 51 | GO:0008152 | P:metabolic process | 68 | 4 | 43 | 0.63 | 0.09 | 0.00746 |
| 91 | GO:0002100 | P:tRNA wobble adenosine to inosine editing | 82 | 3 | 56 | 0.68 | 0.05 | 4.39E-06 |
| 92 | GO:0006310 | P:DNA recombination | 102 | 3 | 81 | 0.79 | 0.03 | 0.000178 |
| 90 | GO:0055114 | P:oxidation reduction | 111 | 17 | 75 | 0.67 | 0.22 | 0.000264 |

| | | | | | | | | |
|---|---|---|---|---|---|---|---|---|
| 47 | GO:0000103 | P:sulfate assimilation | 159 | 4 | 127 | 0.79 | 0.03 | 3.23E-06 |
| 39 | GO:0006355 | P:regulation of transcription, DNA-dependent | 100 | 11 | 83 | 0.83 | 0.13 | 7.21E-06 |
| 53 | GO:0009825 | P:multidimensional cell growth | 114 | 3 | 100 | 0.87 | 0.03 | 4.09E-05 |
| 79 | GO:0009664 | P:plant-type cell wall organization | 73 | 4 | 32 | 0.43 | 0.12 | 2.74E-05 |
| 45 | GO:0006069 | P:ethanol oxidation | 133 | 3 | 89 | 0.66 | 0.03 | 1.92E-06 |
| 3 | GO:0016567 | P:protein ubiquitination | 80 | 3 | 59 | 0.73 | 0.05 | 0.000744 |
| 22 | GO:0042742 | P:defense response to bacterium | 127 | 6 | 96 | 0.75 | 0.06 | 0.000101 |
| 11 | GO:0009086 | P:methionine biosynthetic process | 101 | 3 | 33 | 0.32 | 0.09 | 4.52E-05 |
| 50 | GO:0006270 | P:DNA replication initiation | 132 | 4 | 96 | 0.72 | 0.04 | 1.13E-07 |
| 24 | GO:0009834 | P:secondary cell wall biogenesis | 109 | 6 | 97 | 0.88 | 0.06 | 4.24E-11 |
| 76 | GO:0008152 | P:metabolic process | 55 | 6 | 44 | 0.8 | 0.13 | 3.55E-05 |
| 34 | GO:0010304 | P:PSII associated light-harvesting complex II catabolic process | 138 | 3 | 108 | 0.78 | 0.02 | 4.18E-05 |
| 78 | GO:0009877 | P:nodulation | 95 | 3 | 73 | 0.76 | 0.04 | 0.000144 |
| 94 | GO:0006801 | P:superoxide metabolic process | 77 | 3 | 46 | 0.59 | 0.06 | 0.000058 |
| 9 | GO:0006350 | P:transcription | 77 | 14 | 56 | 0.72 | 0.25 | 7.34E-06 |
| 41 | GO:0005975 | P:carbohydrate metabolic process | 92 | 8 | 71 | 0.77 | 0.11 | 0.000016 |
| 67 | GO:0009734 | P:auxin mediated signaling pathway | 117 | 4 | 76 | 0.64 | 0.05 | 0.0081 |
| 26 | GO:0006085 | P:acetyl-CoA biosynthetic process | 158 | 5 | 158 | 1 | 0.03 | 4.09E-09 |
| 4 | GO:0009813 | P:flavonoid biosynthetic process | 56 | 6 | 38 | 0.67 | 0.15 | 5.94E-07 |
| 33 | GO:0006284 | P:base-excision repair | 54 | 3 | 36 | 0.66 | 0.08 | 1.24E-07 |
| 38 | GO:0009813 | P:flavonoid biosynthetic process | 70 | 3 | 44 | 0.62 | 0.06 | 0.00685 |
| 25 | GO:0016332 | P:establishment or maintenance of polarity of embryonic epithelium | 106 | 6 | 99 | 0.93 | 0.06 | 6.36E-09 |
| 30 | GO:0006089 | P:lactate metabolic process | 99 | 4 | 84 | 0.84 | 0.04 | 1.05E-07 |
| 63 | GO:0016117 | P:carotenoid biosynthetic process | 142 | 4 | 103 | 0.72 | 0.03 | 2.06E-06 |
| 98 | GO:0006412 | P:translation | 154 | 15 | 125 | 0.81 | 0.12 | 1.21E-11 |
| 66 | GO:0048705 | P:skeletal system morphogenesis | 95 | 3 | 70 | 0.73 | 0.04 | 6.85E-06 |

| | | | | | | | | |
|---|---|---|---|---|---|---|---|---|
| 59 | GO:0006869 | P:lipid transport | 98 | 4 | 63 | 0.64 | 0.06 | 0.00174 |
| 37 | GO:0009909 | P:regulation of flower development | 89 | 4 | 57 | 0.64 | 0.07 | 5.63E-07 |
| 20 | GO:0006414 | P:translational elongation | 118 | 4 | 85 | 0.72 | 0.04 | 0.000109 |
| 74 | GO:0006397 | P:mRNA processing | 253 | 16 | 218 | 0.86 | 0.07 | 1.47E-11 |
| 48 | GO:0009765 | P:photosynthesis, light harvesting | 137 | 13 | 102 | 0.74 | 0.12 | 1.22E-11 |
| 86 | GO:0043044 | P:ATP-dependent chromatin remodeling | 191 | 3 | 163 | 0.85 | 0.01 | 5.73E-06 |
| 93 | GO:0008360 | P:regulation of cell shape | 67 | 3 | 50 | 0.74 | 0.06 | 0.000216 |
| 71 | GO:0015031 | P:protein transport | 201 | 15 | 183 | 0.91 | 0.08 | 1.43E-10 |
| 77 | GO:0015979 | P:photosynthesis | 137 | 14 | 114 | 0.83 | 0.12 | 1.66E-11 |
| 21 | GO:0006694 | P:steroid biosynthetic process | 144 | 3 | 81 | 0.56 | 0.03 | 9.68E-06 |
| 19 | GO:0006508 | P:proteolysis | 60 | 5 | 27 | 0.45 | 0.18 | 0.00383 |
| 56 | GO:0010228 | P:vegetative to reproductive phase transition of meristem | 168 | 6 | 161 | 0.95 | 0.03 | 4.31E-11 |
| 69 | GO:0051281 | P:positive regulation of release of sequestered calcium ion into cytosol | 200 | 4 | 11 | 0.05 | 0.36 | 1.22E-07 |
| 16 | GO:0006869 | P:lipid transport | 99 | 4 | 62 | 0.62 | 0.06 | 0.0018 |

The modules were assigned the names according to the most enriched Gene Ontology biological process categories with the smallest P values. Num1 is the number of gene probes in the corresponding module, Num2 the number of genes annotated with the GO term in the module, and Num3 the number of genes that have any GO annotations in the module. Ratio 1 is the ratio of Num3 and Num1 and Ratio2 the ratio of Num2 and Num3.

# Part B

## The list of numbers used to represent different experimental conditions in gene regulatory network figures shown afterwards.

3) Iron_Deficient 50uM Fe(NO3)3 Leaf

4) Iron_Sufficient 100uM Fe(NO3)3 Leaf

5) Psojae Infected Hypocotyl 6h

6) Psojae Infected Hypocotyl 12h

7) Psojae Infected Hypocotyl 24h

8) Psojae Infected Hypocotyl 48h

9) Control Storage Protein Suppression Cotyledon

10) RNAi Storage Protein Suppression1 Cotyledon

11) RNAi Storage Protein Suppression2 Cotyledon

12) Control Oleosin Cotyledon

13) RNAi Oleosin Suppression1 Cotyledon

14) RNAi Oleosin Suppression2 Cotyledon

15) Control 48h Water Leaf

16) Treated 48h NoDBj Leaf

17) Ppachyrhizi Control 0h Leaf

18) Ppachyrhizi Resistant 6h Leaf

19) Ppachyrhizi Susceptible 6h Leaf

20) Ppachyrhizi Resistant 12h Leaf

21) Ppachyrhizi Susceptible 12h Leaf

22) Ppachyrhizi Resistant 48h Leaf

23) Ppachyrhizi Susceptible 48h Leaf

24) Ppachyrhizi Resistant 24h Leaf

25) Ppachyrhizi Susceptible 24h Leaf

26) Ppachyrhizi Uninoculated Leaf

27) Ppachyrhizi Inoculated Leaf

28) Pericycle Cell Control 2days

29) Syncytium Infected 5days

30) Syncytium Infected 10days

31) Syncytium Infected 2days

32) SCN Infected 5daypost inoculation Root

33) Control 10daypost inoculation Root

34) Control 2daypost inoculation Root

35) SCN Infected 2daypost inoculation Root

36) SCN Infected 10daypost inoculation Root

37) Control 5daypost inoculation Root

## Part C

**The details of the modules with correlation coefficient >= 0.800 are described one by one below. Each module has a module ID used in Table S1, a figure visualizing gene regulatory network and gene cluster, the list of enriched Gene Ontology biological processes and the p-values, the IDs and families of predicted transcription factors, and the IDs of genes in the module. If a gene probe maps to several genes, all of them are listed because no further information is available to decide which one was expressed.**

### Module 4

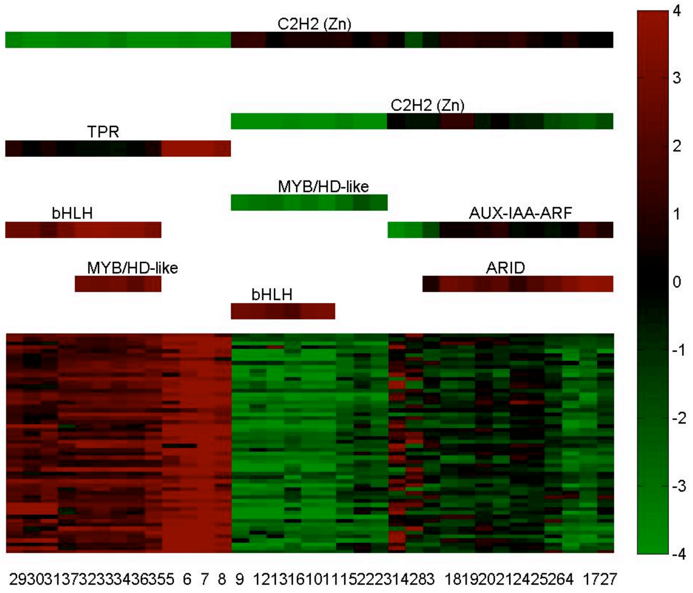


| GO_ACC | GO name | P value |
|---|---|---|
| GO:0042744 | P:hydrogen peroxide catabolic process | 0.00051864 |
| GO:0006952 | P:defense response | 0.003958 |
| GO:0009607 | P:response to biotic stimulus | 0.0010285 |
| GO:0009813 | P:flavonoid biosynthetic process | 0.000000594 |
| GO:0055114 | P:oxidation reduction | 1.99E-10 |

| TF | family | Binding site | Gene number |
|---|---|---|---|
| Glyma03 g00980 | MYB/HD -like | | |
| Glyma03 g34110 | MYB/HD -like | | |
| Glyma04 g02210 | ARID | | |
| Glyma07 g19540 | C2H2 (Zn) | 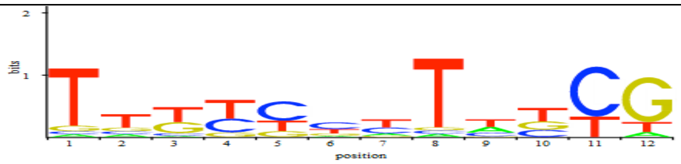 | 30 |

| | | | |
|---|---|---|---|
| Glyma10 g32340 | AUX-IAA-ARF | | |
| Glyma13 g34530 | C2H2 (Zn) | 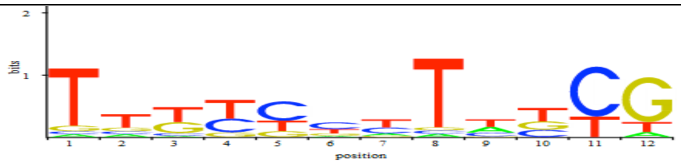  | 30 |
| Glyma15 g06680 | bHLH | | |
| Glyma19 g27480 | bHLH | | |
| Glyma12 g31490 | TPR | | |

Glyma01g31540 Glyma11g02770 Glyma06g12010 Glyma15g15680 Glyma04g04270 Glyma13g05240 Glyma09g02600 Glyma18g45260 Glyma01g42660 Glyma11g07490 Glyma06g15030 Glyma16g02570 Glyma04g06150 Glyma13g31590 Glyma09g05440 Glyma18g52250 Glyma02g18380 Glyma11g11530 Glyma06g41610 Glyma16g27880 Glyma04g08520 Glyma13g34520 Glyma09g40580 Glyma19g32650 Glyma02g42470 Glyma11g21260 Glyma06g45940 Glyma17g03350 Glyma04g19860 Glyma13g34530 Glyma09g40590 Glyma19g32700 Glyma03g29950 Glyma12g02250 Glyma07g34010 Glyma17g15690 Glyma04g27580 Glyma13g36910 Glyma09g41840 Glyma19g32880 Glyma03g30390 Glyma12g03680 Glyma08g18170 Glyma17g33370 Glyma04g39860 Glyma14g06400 Glyma10g29280 Glyma19g33320 Glyma03g30400 Glyma12g10790 Glyma08g37670 Glyma18g28830 Glyma05g05420 Glyma15g07710 Glyma10g33650 Glyma20g01670 Glyma03g39610 Glyma12g16570 Glyma08g37680 Glyma18g41910 Glyma06g03860 Glyma15g13500 Glyma10g42230 Glyma20g24810 Glyma04g03770 Glyma12g33550 Glyma08g45520 Glyma18g45250 Glyma20g27480 Glyma20g38000 Glyma20g38140

# Module 9

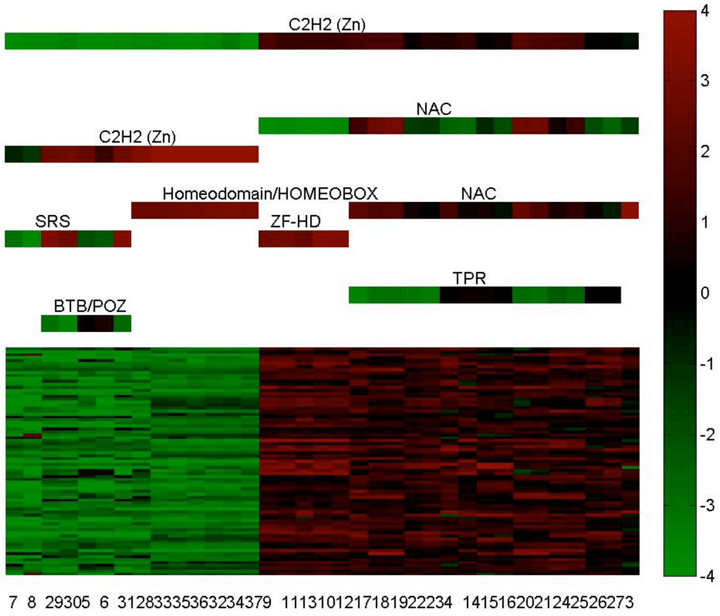


| GO_ACC | GO name | P value |
|---|---|---|
| GO:0006350 | P:transcription | 0.00000734 |
| GO:0006355 | P:regulation of transcription, DNA-dependent | 0.0053857 |
| GO:0045449 | P:regulation of transcription | 0.00017243 |

| TF | Family | Binding site | Gene number |
|---|---|---|---|
| Glyma02 g16840 | BTB/POZ | | |
| Glyma02 g44860 | SRS | | |
| Glyma04 g09910 | ZF-HD | | |
| Glyma08 g18470 | NAC | | |
| Glyma08 g46090 | TPR | | |

| | | | |
|---|---|---|---|
| Glyma10 g41870 | C2H2 (Zn) | 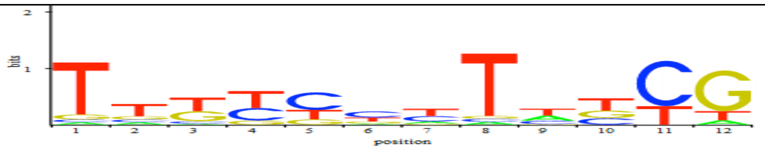 | 39 |
| Glyma12 g22880 | NAC | | |
| Glyma13 g40240 | C2H2 (Zn) | 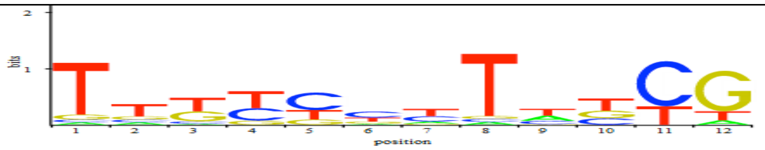 | 39 |
| Glyma14 g09310 | Homeodo main/HO MEOBOX | 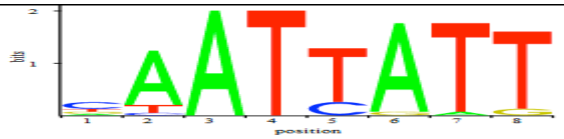 | 31 |

Glyma0048s00300 Glyma10g40120 Glyma06g12760 Glyma14g24220 Glyma03g01860
Glyma13g10360 Glyma08g19410 Glyma17g35530 Glyma01g05150 Glyma10g42660 Glyma06g18610
Glyma15g05580 Glyma03g29710 Glyma13g22500 Glyma09g07090 Glyma18g08960 Glyma01g38650
Glyma11g06640 Glyma06g35580 Glyma15g09370 Glyma03g31570 Glyma13g22620 Glyma09g08170
Glyma18g11990 Glyma01g44730 Glyma11g15950 Glyma06g43570 Glyma15g18380 Glyma03g39400
Glyma13g29690 Glyma09g11740 Glyma18g40690 Glyma02g02350 Glyma11g19400 Glyma06g43590
Glyma15g23480 Glyma04g09420 Glyma13g33920 Glyma09g16080 Glyma19g07410 Glyma02g09650
Glyma12g07510 Glyma07g05580 Glyma16g02110 Glyma04g19880 Glyma13g34690 Glyma09g25100
Glyma19g32570 Glyma02g16050 Glyma12g07890 Glyma07g06300 Glyma16g02940 Glyma04g42010
Glyma13g36560 Glyma09g27870 Glyma20g00850 Glyma02g23860 Glyma12g14360 Glyma07g08040
Glyma16g32690 Glyma04g42260 Glyma13g38430 Glyma10g29370 Glyma20g24370 Glyma02g25260
Glyma12g20870 Glyma07g08090 Glyma16g32740 Glyma05g04260 Glyma13g38750 Glyma10g29380
Glyma20g27290 Glyma02g28300 Glyma12g29370 Glyma07g08340 Glyma17g00590 Glyma05g24070
Glyma13g40240 Glyma10g33710 Glyma20g30070 Glyma02g43150 Glyma12g31670 Glyma07g19540
Glyma17g11310 Glyma05g25910 Glyma14g00700 Glyma10g35050 Glyma20g32500 Glyma02g43740
Glyma12g32050 Glyma07g30180 Glyma17g12200 Glyma06g09970 Glyma14g05160 Glyma10g35060
Glyma20g32510 Glyma02g43750 Glyma12g35720 Glyma08g06550 Glyma17g14710 Glyma06g12550
Glyma14g05170 Glyma10g37770 Glyma20g33880 Glyma20g36040

# Module 16

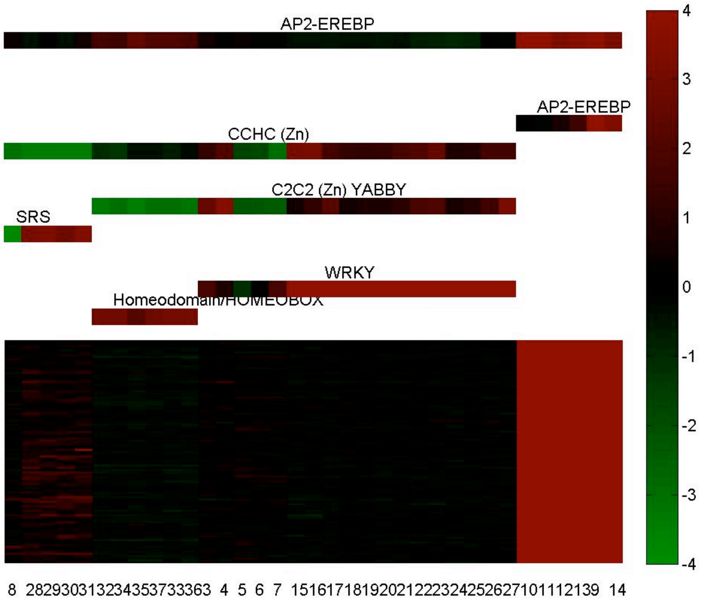


| GO_ACC | GO name | P value |
|---|---|---|
| GO:0006950 | P:response to stress | 0.02 |
| GO:0006869 | P:lipid transport | 0.0018016 |
| GO:0055085 | P:transmembrane transport | 0.0085966 |

| TF | Family | Binding site | Gene number |
|---|---|---|---|
| Glyma02g44860 | SRS | | |
| Glyma06g15220 | WRKY | | |
| Glyma09g08330 | AP2-EREBP | | |
| Glyma13g00950 | AP2-EREBP | | |
| Glyma17g14710 | C2C2 (Zn) YABBY | 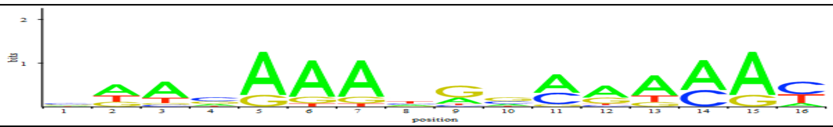 | 53 |

| | | | |
|---|---|---|---|
| Glyma19g41150 | CCHC (Zn) | 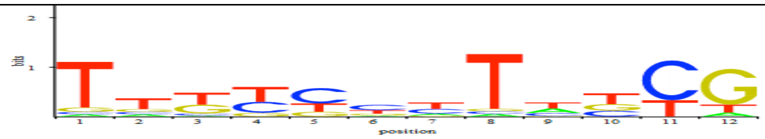 | 53 |
| Glyma19g41610 | Homeodomain /HOMEOBOX | 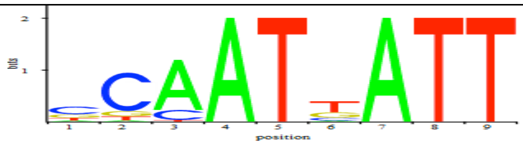 | 16 |

Glyma01g10900 Glyma11g07020 Glyma06g02160 Glyma15g12380 Glyma04g01190 Glyma13g33540 Glyma09g09930 Glyma18g38490 Glyma01g38340 Glyma11g07260 Glyma06g08290 Glyma15g15260 Glyma04g02060 Glyma13g36400 Glyma09g28700 Glyma18g43320 Glyma01g43780 Glyma11g15870 Glyma06g12730 Glyma15g21980 Glyma04g08220 Glyma13g42310 Glyma09g28710 Glyma18g45060 Glyma02g01590 Glyma11g16090 Glyma06g16380 Glyma16g00950 Glyma04g17670 Glyma13g42320 Glyma09g28720 Glyma18g47750 Glyma02g08400 Glyma11g19070 Glyma06g19000 Glyma16g07800 Glyma04g17680 Glyma13g44260 Glyma09g28730 Glyma19g41480 Glyma02g26860 Glyma11g36780 Glyma06g23340 Glyma16g33400 Glyma04g35950 Glyma14g03730 Glyma09g28930 Glyma19g41560 Glyma02g42000 Glyma12g13300 Glyma06g44440 Glyma16g33530 Glyma04g38620 Glyma14g05750 Glyma09g38570 Glyma19g42420 Glyma02g43670 Glyma12g34160 Glyma07g04260 Glyma17g13120 Glyma04g42060 Glyma14g06900 Glyma09g40750 Glyma19g43100 Glyma02g45050 Glyma13g09530 Glyma07g30410 Glyma17g14900 Glyma05g04450 Glyma14g09910 Glyma10g04280 Glyma20g24530 Glyma02g45070 Glyma13g17980 Glyma08g09310 Glyma17g34030 Glyma05g07880 Glyma14g11770 Glyma10g29630 Glyma20g28460 Glyma03g32020 Glyma13g18450 Glyma08g18400 Glyma17g35260 Glyma05g08880 Glyma14g15020 Glyma10g31750 Glyma20g28550 Glyma03g32030 Glyma13g20680 Glyma08g40260 Glyma17g37530 Glyma05g26400 Glyma14g24860 Glyma10g33760 Glyma20g28640 Glyma03g38900 Glyma13g23980 Glyma08g47240 Glyma18g00690 Glyma05g27840 Glyma14g26400 Glyma10g39160 Glyma20g33850 Glyma03g39860 Glyma13g26330 Glyma09g01480 Glyma18g17490 Glyma05g33200 Glyma14g26410 Glyma10g39170 Glyma20g35860 Glyma04g01180 Glyma13g26340 Glyma09g04250 Glyma18g32760 Glyma05g34030 Glyma15g01000 Glyma10g42470 Glyma20g36210 Glyma05g38250 Glyma15g03030 Glyma11g01730 Glyma20g37670

# Module 19

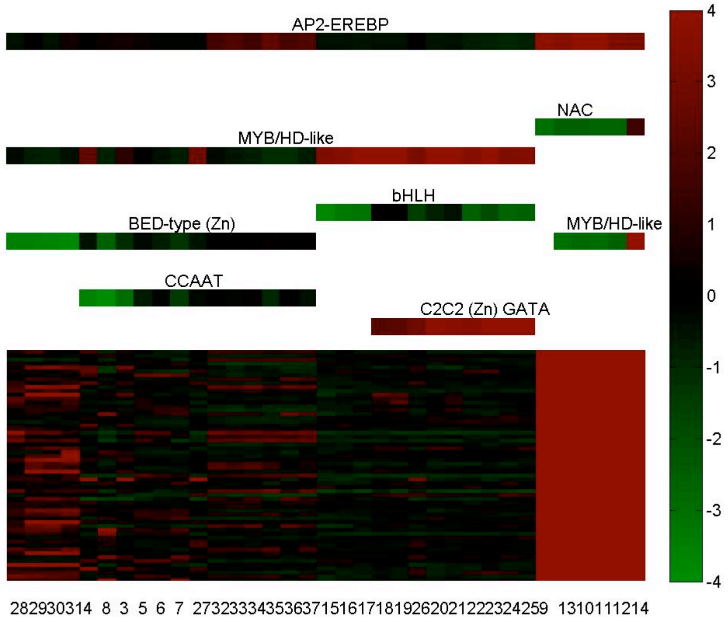


| GO_ACC | GO name | P value |
|---|---|---|
| GO:0006508 | P:proteolysis | 0.0038279 |

| TF | Family | Binding site | Gene number |
|---|---|---|---|
| Glyma01g02390 | bHLH | | |
| Glyma05g32850 | NAC | | |
| Glyma07g04050 | CCAAT | | |
| Glyma08g04670 | MYB/HD-like | | |
| Glyma09g15130 | BED-type (Zn) | | |

| | | | |
|---|---|---|---|
| Glyma13g00950 | AP2-EREBP | 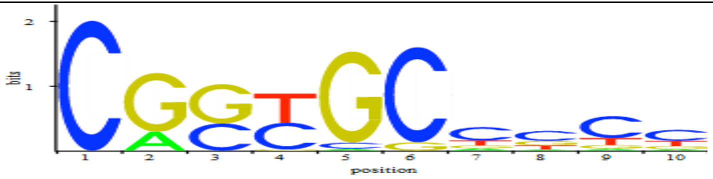 | 11 |
| Glyma17g06290 | C2C2 (Zn) GATA | | |
| Glyma19g29750 | MYB/HD-like | | |

Glyma01g06820 Glyma11g21480 Glyma07g38790 Glyma17g00950 Glyma04g01190 Glyma13g44260 Glyma09g38570 Glyma19g41480 Glyma01g43780 Glyma11g36840 Glyma07g39820 Glyma17g01920 Glyma04g37410 Glyma14g04790 Glyma10g03390 Glyma19g41560 Glyma02g16440 Glyma12g13300 Glyma08g01390 Glyma17g03040 Glyma05g04230 Glyma15g00450 Glyma10g08370 Glyma19g42490 Glyma03g30040 Glyma12g33320 Glyma08g12270 Glyma17g09390 Glyma05g28400 Glyma15g01000 Glyma10g25350 Glyma20g24530 Glyma03g34760 Glyma13g29550 Glyma08g19340 Glyma17g14680 Glyma05g33340 Glyma15g03030 Glyma10g31540 Glyma20g28650 Glyma03g37140 Glyma13g34460 Glyma08g45480 Glyma17g16620 Glyma06g16800 Glyma15g05650 Glyma10g35100 Glyma20g28660 Glyma03g38900 Glyma13g37110 Glyma08g45530 Glyma19g13060 Glyma06g44440 Glyma16g25910 Glyma10g39150 Glyma20g30460 Glyma03g39940 Glyma13g44020 Glyma08g46080 Glyma19g32920 Glyma06g45210 Glyma16g25920 Glyma10g42470 Glyma20g32460 Glyma11g01730 Glyma20g36040

## Module 20

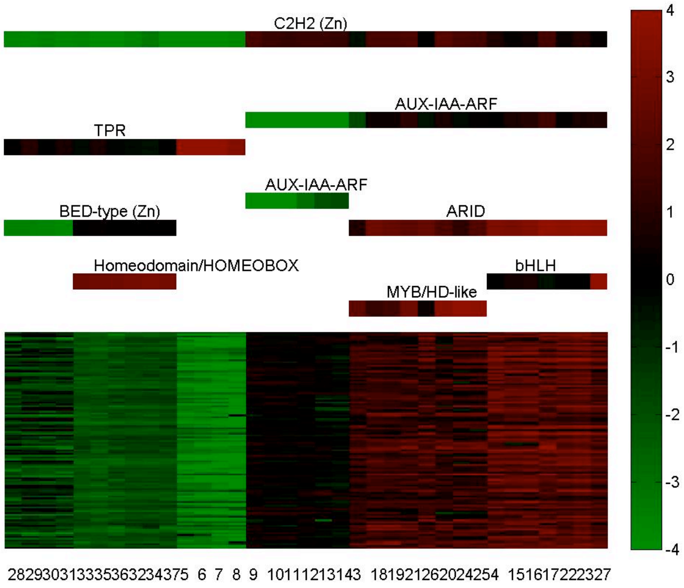

| GO_ACC | GO name | P value |
|---|---|---|
| GO:0022900 | P:electron transport chain | 0.02 |
| GO:0006414 | P:translational elongation | 0.00010901 |
| GO:0006260 | P:DNA replication | 0.01 |
| GO:0006412 | P:translation | 0.0057385 |
| GO:0006810 | P:transport | 0.0033568 |
| GO:0006811 | P:ion transport | 0.00055224 |
| GO:0006457 | P:protein folding | 0.0038928 |
| GO:0008033 | P:tRNA processing | 0.0002736 |
| GO:0015979 | P:photosynthesis | 0.00015389 |
| GO:0051301 | P:cell division | 0.03 |
| GO:0009408 | P:response to heat | 0.00088292 |

| TF | Family | Binding site | Gene number |
|---|---|---|---|
| Glyma03g28630 | bHLH | | |
| Glyma04g02210 | ARID | | |
| Glyma08g04670 | MYB/HD-like | | |
| Glyma09g15130 | BED-type (Zn) | | |
| Glyma10g32340 | AUX-IAA-ARF | | |
| Glyma13g40240 | C2H2 (Zn) | 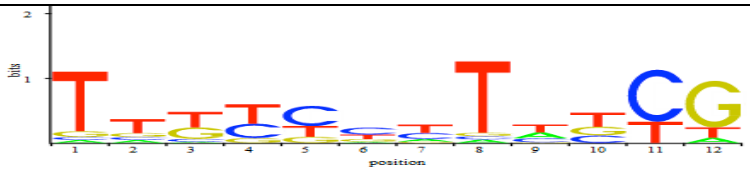 | 49 |
| Glyma14g09310 | Homeodomain/HOMEOBOX | | |
| Glyma15g02040 | AUX-IAA-ARF | | |

| | | | |
|---|---|---|---|
| Glyma12g31490 | TPR | | |

Glyma01g00590 Glyma09g15370 Glyma05g32170 Glyma13g43360 Glyma03g07880 Glyma11g18410 Glyma07g15500 Glyma17g37740 Glyma01g22490 Glyma09g24780 Glyma05g32530 Glyma14g06590 Glyma03g19030 Glyma11g19800 Glyma07g21150 Glyma18g05640 Glyma01g34700 Glyma09g29370 Glyma05g33510 Glyma14g17100 Glyma03g31470 Glyma11g20080 Glyma07g33630 Glyma18g08420 Glyma01g37900 Glyma09g32600 Glyma05g33640 Glyma14g23860 Glyma03g33430 Glyma11g31620 Glyma07g35510 Glyma18g11990 Glyma01g38940 Glyma09g35750 Glyma05g35470 Glyma14g37780 Glyma03g37800 Glyma11g31680 Glyma08g02800 Glyma18g19730 Glyma01g41400 Glyma09g36710 Glyma06g05810 Glyma14g40430 Glyma03g41420 Glyma12g00300 Glyma08g04260 Glyma18g22150 Glyma01g42290 Glyma09g39130 Glyma06g05850 Glyma14g40490 Glyma03g42050 Glyma12g01600 Glyma08g06220 Glyma18g26600 Glyma01g44220 Glyma10g02930 Glyma06g05880 Glyma15g05210 Glyma04g05800 Glyma12g03280 Glyma08g07880 Glyma18g47200 Glyma01g44850 Glyma10g08690 Glyma06g07110 Glyma15g10460 Glyma04g05860 Glyma12g05650 Glyma08g12060 Glyma18g49890 Glyma02g07350 Glyma10g12210 Glyma06g09420 Glyma15g12130 Glyma04g05890 Glyma12g08390 Glyma08g12070 Glyma19g08990 Glyma02g11110 Glyma10g20570 Glyma06g13140 Glyma15g17580 Glyma04g07010 Glyma12g09850 Glyma08g13790 Glyma19g34310 Glyma02g11390 Glyma10g27610 Glyma06g13600 Glyma15g29900 Glyma04g16410 Glyma12g22700 Glyma08g15460 Glyma19g35160 Glyma02g12680 Glyma10g29800 Glyma06g13770 Glyma15g36680 Glyma04g37960 Glyma12g27060 Glyma08g16100 Glyma19g36160 Glyma02g16850 Glyma10g29810 Glyma06g22920 Glyma15g42610 Glyma04g40650 Glyma12g33430 Glyma08g16370 Glyma19g44010 Glyma02g25260 Glyma11g00800 Glyma06g30340 Glyma16g02940 Glyma04g41070 Glyma13g02720 Glyma08g23450 Glyma19g44760 Glyma02g29500 Glyma11g04020 Glyma06g38470 Glyma16g13830 Glyma04g41080 Glyma13g03430 Glyma08g26020 Glyma20g00410 Glyma02g36230 Glyma11g07160 Glyma06g42760 Glyma16g26340 Glyma04g41250 Glyma13g11740 Glyma08g26340 Glyma20g01120 Glyma02g36370 Glyma11g07450 Glyma06g46570 Glyma17g05150 Glyma05g04210 Glyma13g17350 Glyma08g36390 Glyma20g03950 Glyma02g42300 Glyma11g10200 Glyma06g48340 Glyma17g08310 Glyma05g16360 Glyma13g19840 Glyma08g39280 Glyma20g21520 Glyma02g44000 Glyma11g10550 Glyma07g02570 Glyma17g12370 Glyma05g24660 Glyma13g23630 Glyma09g06300 Glyma20g37520 Glyma03g00440 Glyma11g11080 Glyma07g06300 Glyma17g14650 Glyma05g28900 Glyma13g28660 Glyma20g37530 Glyma03g01060 Glyma11g13650 Glyma07g07570 Glyma17g29660 Glyma05g30600 Glyma13g37010

# Module 21

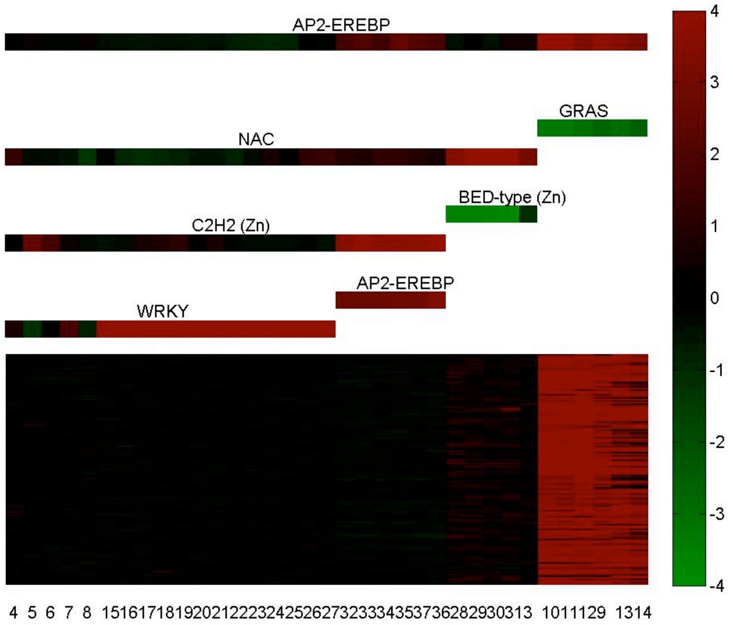


| GO_ACC | GO name | P value |
|---|---|---|
| GO:0016126 | P:sterol biosynthetic process | 0.0000474 |
| GO:0006355 | P:regulation of transcription, DNA-dependent | 0.01 |
| GO:0006508 | P:proteolysis | 0.04 |
| GO:0006694 | P:steroid biosynthetic process | 0.00000968 |
| GO:0007155 | P:cell adhesion | 0.0000867 |
| GO:0009651 | P:response to salt stress | 0.0098528 |

| TF | Family | Binding site | Gene number |
|---|---|---|---|
| Glyma01 g38360 | GRAS | 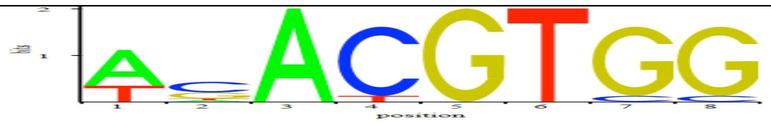 | 20 |
| Glyma06 g15220 | WRKY | | |

| | | | |
|---|---|---|---|
| Glyma09 g04630 | AP2-EREBP | 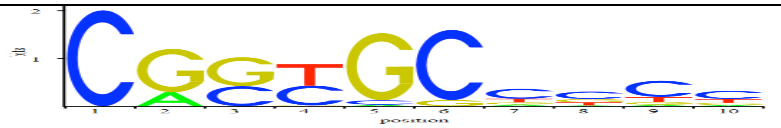 | 21 |
| Glyma09 g15130 | BED-type (Zn) | 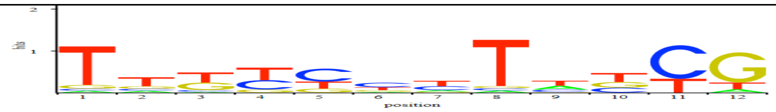 | 58 |
| Glyma13 g00950 | AP2-EREBP | 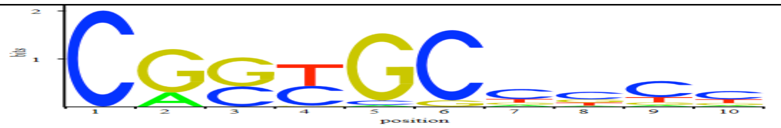 | 21 |
| Glyma14 g13360 | C2H2 (Zn) | 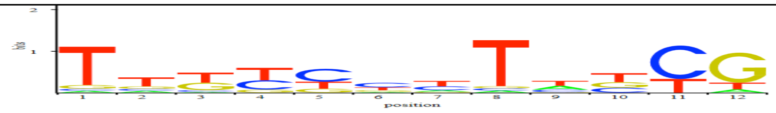 | 58 |
| Glyma19 g08510 | NAC | | |

Glyma01g21890 Glyma11g33940 Glyma07g23510 Glyma16g08160 Glyma04g27740 Glyma13g35610 Glyma09g06540 Glyma18g46650 Glyma01g29480 Glyma12g03570 Glyma07g26260 Glyma16g27460 Glyma04g42050 Glyma13g36780 Glyma09g06550 Glyma18g46680 Glyma01g35580 Glyma12g06970 Glyma07g28940 Glyma16g31330 Glyma04g43640 Glyma13g39340 Glyma09g06900 Glyma18g47060 Glyma01g38340 Glyma12g10390 Glyma07g37030 Glyma16g31580 Glyma0554s0022Glyma13g43220 Glyma09g11910 Glyma18g51290 Glyma01g40540 Glyma12g11770 Glyma07g39820 Glyma16g31640 Glyma05g07630 Glyma14g01700 Glyma09g25830 Glyma19g00400 Glyma01g42600 Glyma12g26170 Glyma08g02260 Glyma16g31690 Glyma05g08880 Glyma14g01710 Glyma09g35170 Glyma19g24600 Glyma02g08520 Glyma12g34960 Glyma08g08200 Glyma16g33120 Glyma05g25180 Glyma14g03660 Glyma09g36940 Glyma19g27340 Glyma02g09320 Glyma12g36580 Glyma08g11400 Glyma16g33140 Glyma05g37290 Glyma14g09710 Glyma09g40550 Glyma19g29220 Glyma02g12360 Glyma13g01680 Glyma08g17920 Glyma17g04550 Glyma06g02500 Glyma14g12110 Glyma09g41730 Glyma19g29660 Glyma02g31910 Glyma13g01700 Glyma08g23480 Glyma17g04620 Glyma06g05310 Glyma14g36090 Glyma10g07410 Glyma19g29880 Glyma02g37040 Glyma13g01710 Glyma08g24530 Glyma17g09110 Glyma06g07630 Glyma15g01000 Glyma10g15240 Glyma19g29920 Glyma02g38690 Glyma13g08880 Glyma08g24540 Glyma17g19810 Glyma06g16610 Glyma15g02120 Glyma10g35010 Glyma19g34770 Glyma02g42600 Glyma13g08890 Glyma08g38190 Glyma17g29170 Glyma06g42480 Glyma15g16670 Glyma10g38090 Glyma19g34780 Glyma02g46820 Glyma13g10590 Glyma08g43620 Glyma17g33790 Glyma06g42520 Glyma15g18190 Glyma10g39270 Glyma19g39840 Glyma02g47030 Glyma13g17880 Glyma08g43900 Glyma18g00400 Glyma06g42630 Glyma15g35880 Glyma10g41250 Glyma19g42490 Glyma03g00800 Glyma13g17980 Glyma08g43920 Glyma18g11820 Glyma06g42780 Glyma15g39880 Glyma10g42470 Glyma20g03080 Glyma03g01000 Glyma13g21280 Glyma08g45840 Glyma18g29400 Glyma06g47940 Glyma16g00970 Glyma11g04320 Glyma20g06750 Glyma03g07470 Glyma13g26160

Glyma08g47250 Glyma18g43250 Glyma06g48270 Glyma16g04180 Glyma11g11410 Glyma20g19620
Glyma03g10390 Glyma13g26180 Glyma09g03040 Glyma18g43940 Glyma07g04290 Glyma16g05480
Glyma11g26000 Glyma20g19630 Glyma03g34680 Glyma13g26510 Glyma09g05330 Glyma18g45060
Glyma07g09290 Glyma16g05650 Glyma11g26060 Glyma20g29730 Glyma04g12660 Glyma13g26520
Glyma09g06100 Glyma18g45070 Glyma07g16090 Glyma16g06600 Glyma11g26090 Glyma20g32550
Glyma04g14830 Glyma13g31120 Glyma09g06130 Glyma18g45280 Glyma11g27150 Glyma20g38650

## Module 24

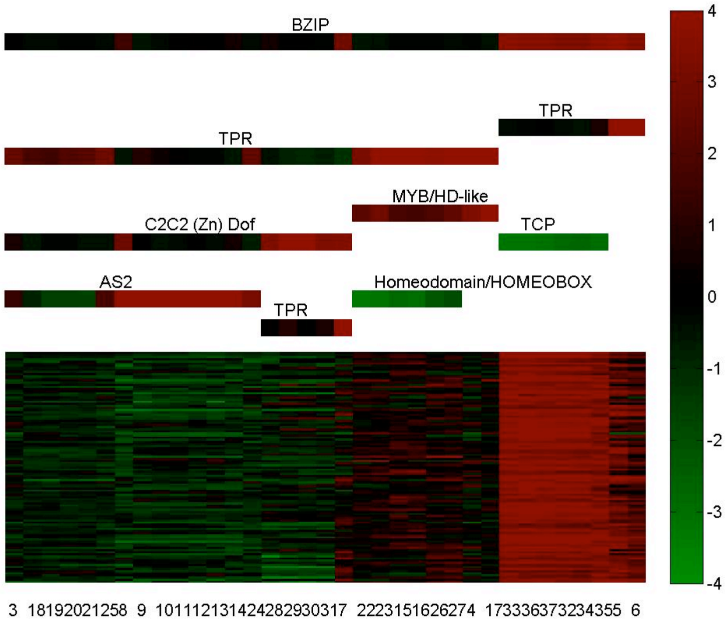


| GO_ACC | GO name | P value |
|---|---|---|
| GO:0009809 | P:lignin biosynthetic process | 0.00072254 |
| GO:0043086 | P:negative regulation of catalytic activity | 0.0020533 |
| GO:0042744 | P:hydrogen peroxide catabolic process | 0.03 |
| GO:0030245 | P:cellulose catabolic process | 0.0000568 |
| GO:0022900 | P:electron transport chain | 0.02 |
| GO:0009834 | P:secondary cell wall biogenesis | 4.24E-11 |
| GO:0055114 | P:oxidation reduction | 0.0018292 |
| GO:0008152 | P:metabolic process | 0.001507 |
| GO:0007275 | P:multicellular organismal development | 0.01 |
| GO:0007047 | P:cellular cell wall organization | 0.000000708 |
| GO:0006810 | P:transport | 0.04 |
| GO:0006284 | P:base-excision repair | 0.00000105 |
| GO:0005975 | P:carbohydrate metabolic process | 0.0021711 |
| GO:0030244 | P:cellulose biosynthetic process | 0.00035277 |

| | | |
|---|---|---|
| GO:0009058 | P:biosynthetic process | 0.0023039 |

| TF | Family | Binding site | Gene number |
|---|---|---|---|
| Glyma06g30000 | TPR | | |
| Glyma12g33430 | MYB/HD-like | 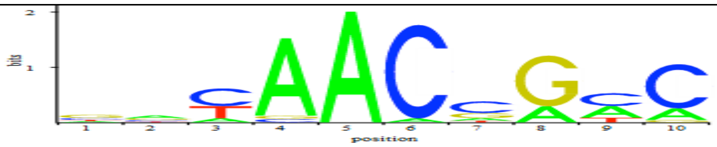 | 28 |
| Glyma13g07590 | AS2 | | |
| Glyma13g30330 | C2C2 (Zn) Dof | | |
| Glyma13g34690 | TCP | | |
| Glyma14g10430 | Homeodomain/HOMEOBOX | | |
| Glyma12g31490 | TPR | | |
| Glyma12g30990 | BZIP | | |

Glyma0022s00480 Glyma11g11840 Glyma05g36930 Glyma15g13080 Glyma03g14450 Glyma13g34700 Glyma08g22120 Glyma18g12210 Glyma0041s00240 Glyma11g13580 Glyma06g01940Glyma05g31460 Glyma15g05150 Glyma11g05660 Glyma20g32140 Glyma0335s00200 Glyma15g13540Glyma03g32360 Glyma13g36280 Glyma08g27570 Glyma18g20510 Glyma0041s00250 Glyma11g18160 Glyma06g03410 Glyma16g02200 Glyma03g36000 Glyma13g39730 Glyma08g39140 Glyma18g42970 Glyma01g01450 Glyma11g19490 Glyma06g03830 Glyma16g02760 Glyma03g37110 Glyma13g39740 Glyma09g01980 Glyma18g44810 Glyma01g20740 Glyma11g19890 Glyma06g04810 Glyma16g14690 Glyma03g38800 Glyma13g40000 Glyma09g02160 Glyma18g49020 Glyma01g23040 Glyma11g29010 Glyma06g06870 Glyma16g21070 Glyma03g40020 Glyma13g40600 Glyma09g02670 Glyma18g50770 Glyma01g23050

Glyma11g33330 Glyma06g12340 Glyma16g26630 Glyma03g41830 Glyma13g44950 Glyma09g08120
Glyma19g01100 Glyma01g27710 Glyma11g35030 Glyma06g14210 Glyma16g28340
Glyma0428s00200 Glyma14g04530 Glyma09g23330 Glyma19g01450 Glyma01g28760
Glyma11g35930 Glyma06g14220 Glyma16g29330 Glyma04g01830 Glyma14g04980 Glyma09g23600
Glyma19g03500 Glyma01g32450 Glyma11g36620 Glyma06g18370 Glyma16g29340 Glyma04g03750
Glyma14g06760 Glyma09g23610 Glyma19g04220 Glyma01g37720 Glyma12g04120 Glyma06g33130
Glyma16g29370 Glyma04g04730 Glyma14g07700 Glyma09g27390 Glyma19g35090 Glyma01g39350
Glyma12g05580 Glyma06g34340 Glyma16g32490 Glyma04g06780 Glyma14g07980 Glyma09g34320
Glyma19g38600 Glyma01g39590 Glyma12g08590 Glyma06g46530 Glyma17g06580 Glyma04g08290
Glyma14g09310 Glyma09g41000 Glyma19g39730 Glyma02g00280 Glyma12g10100 Glyma06g48360
Glyma17g08000 Glyma04g22530 Glyma14g12710 Glyma10g00260 Glyma19g42560 Glyma02g08920
Glyma12g10240 Glyma07g00840 Glyma17g10510 Glyma04g36520 Glyma14g35550 Glyma10g12440
Glyma19g44480 Glyma02g09110 Glyma12g29790 Glyma07g05660 Glyma17g13550 Glyma04g40580
Glyma14g38080 Glyma10g31020 Glyma20g12150 Glyma02g11530 Glyma12g30160 Glyma07g06150
Glyma17g33470 Glyma04g42460 Glyma14g38090 Glyma10g32120 Glyma20g12220 Glyma02g14390
Glyma12g34280 Glyma07g17820 Glyma17g35880 Glyma04g43540 Glyma14g38100 Glyma10g35380
Glyma20g12230 Glyma02g14400 Glyma12g35710 Glyma07g32440 Glyma17g37040 Glyma05g02890
Glyma14g38110 Glyma10g39040 Glyma20g24670 Glyma02g36720 Glyma13g00450 Glyma07g33780
Glyma17g37270 Glyma05g08530 Glyma15g00390 Glyma10g42390 Glyma20g28290 Glyma02g43910
Glyma13g01610 Glyma08g02610 Glyma17g37280 Glyma05g25780 Glyma15g04820 Glyma10g42400
Glyma20g28790 Glyma02g44030 Glyma13g03650 Glyma08g04370 Glyma18g02470 Glyma13g06050
Glyma08g08730 Glyma18g03330 Glyma05g33420 Glyma15g08660 Glyma11g05930 Glyma20g35500
Glyma03g04440 Glyma13g24140 Glyma08g14680 Glyma18g04900 Glyma05g35350 Glyma15g12900
Glyma11g07580 Glyma20g38570 Glyma03g04450 Glyma13g30590 Glyma08g19860 Glyma18g06670

**Module 25**

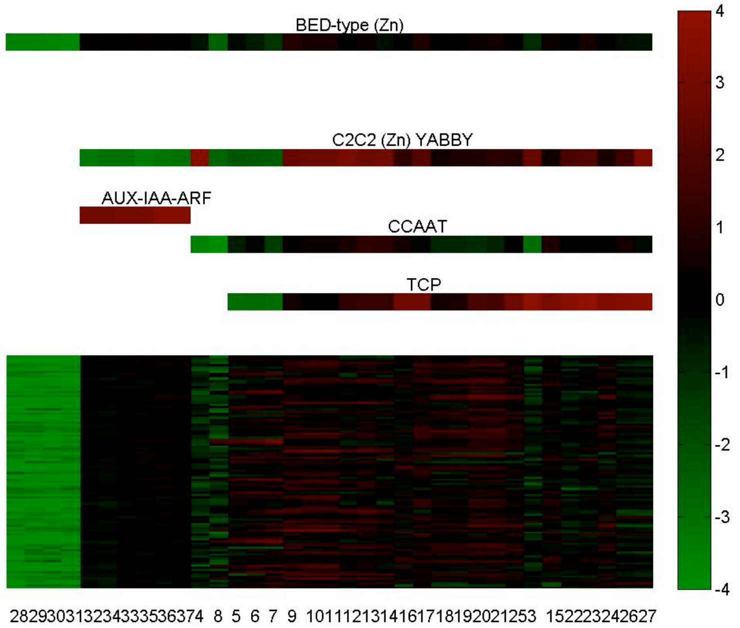


| | | |
|---|---|---|
| GO:0016055 | P:Wnt receptor signaling pathway | 0.00040323 |
| GO:0055085 | P:transmembrane transport | 0.00061307 |
| GO:0019509 | P:L-methionine salvage from methylthioadenosine | 0.0000522 |
| GO:0016332 | P:establishment or maintenance of polarity of... | 6.36E-09 |
| GO:0009062 | P:fatty acid catabolic process | 0.00000953 |
| GO:0006468 | P:protein amino acid phosphorylation | 0.00083103 |
| GO:0006413 | P:translational initiation | 0.0000718 |
| GO:0006096 | P:glycolysis | 0.02 |
| GO:0015031 | P:protein transport | 0.0021248 |
| GO:0016567 | P:protein ubiquitination | 0.0016804 |

| TF | Family | Binding site | Gene number |
|---|---|---|---|
| Glyma02 g38260 | AUX-IAA-ARF | | |
| Glyma07 g04050 | CCAAT | | |
| Glyma09 g15130 | BED-type (Zn) | 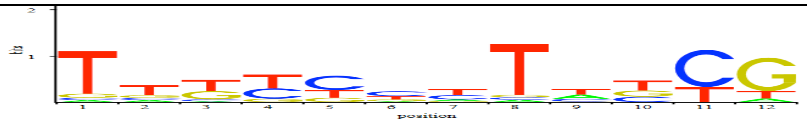 | 56 |

| | | | |
|---|---|---|---|
| Glyma17 g14710 | C2C2 (Zn) YABBY | 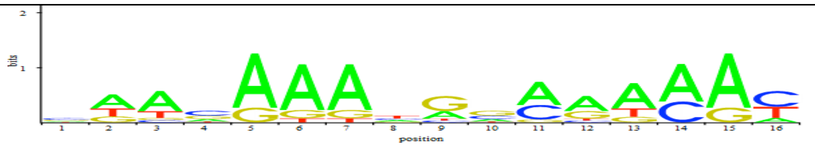 | 56 |
| Glyma19 g03810 | TCP | | |

Glyma0022s0042 Glyma09g30320 Glyma04g08310 Glyma15g28010 Glyma02g10710 Glyma11g31810 Glyma07g07210 Glyma18g50340 Glyma01g00760 Glyma09g34330 Glyma04g08530 Glyma15g38730 Glyma02g16010 Glyma11g34340 Glyma07g11890 Glyma18g52110 Glyma01g01060 Glyma09g39110 Glyma04g14270 Glyma15g38780 Glyma02g16030 Glyma11g34460 Glyma07g12190 Glyma18g52940 Glyma01g01070 Glyma10g01280 Glyma04g31830 Glyma15g39770 Glyma02g16840 Glyma12g03400 Glyma07g13570 Glyma19g00810 Glyma01g01440 Glyma10g02940 Glyma04g32680 Glyma16g03640 Glyma02g17390 Glyma12g07790 Glyma07g15300 Glyma19g27280 Glyma01g02250 Glyma10g03740 Glyma04g40150 Glyma16g04580 Glyma02g18320 Glyma12g11240 Glyma07g15890 Glyma19g28300 Glyma01g03130 Glyma10g05830 Glyma04g41350 Glyma16g04910 Glyma02g36360 Glyma12g12530 Glyma07g30150 Glyma19g31200 Glyma01g04570 Glyma10g06250 Glyma04g42250 Glyma16g05500 Glyma02g36660 Glyma12g15210 Glyma07g35980 Glyma19g32500 Glyma01g20780 Glyma10g10960 Glyma05g21220 Glyma16g16060 Glyma02g39160 Glyma12g29590 Glyma07g37570 Glyma19g35550 Glyma01g27120 Glyma10g30020 Glyma05g35680 Glyma16g17060 Glyma02g40050 Glyma13g20560 Glyma07g39550 Glyma19g37230 Glyma01g31460 Glyma10g34440 Glyma06g04760 Glyma17g00700 Glyma02g40900 Glyma13g29410 Glyma07g39870 Glyma19g44170 Glyma01g33220 Glyma10g38940 Glyma06g08430 Glyma17g00910 Glyma02g44340 Glyma13g40140 Glyma08g04000 Glyma19g44310 Glyma01g34780 Glyma10g39490 Glyma06g08640 Glyma17g01200 Glyma03g03800 Glyma13g42170 Glyma08g07160 Glyma19g45410 Glyma01g39000 Glyma10g41600 Glyma06g12570 Glyma17g01210 Glyma03g05120 Glyma13g43130 Glyma08g21530 Glyma20g12170 Glyma01g41230 Glyma10g42480 Glyma06g14690 Glyma17g03070 Glyma03g10370 Glyma14g04470 Glyma08g21720 Glyma20g23830 Glyma01g45290 Glyma10g43090 Glyma06g21790 Glyma17g03080 Glyma03g14780 Glyma14g37260 Glyma08g22620 Glyma20g24540 Glyma01g45300 Glyma10g43700 Glyma06g23200 Glyma17g08320 Glyma03g20850 Glyma14g38240 Glyma08g23370 Glyma20g25620 Glyma01g45360 Glyma11g00390 Glyma06g37390 Glyma17g18310 Glyma03g28460 Glyma14g39220 Glyma08g23680 Glyma20g28260 Glyma02g00220 Glyma11g00440 Glyma06g44810 Glyma18g03880 Glyma03g29660 Glyma15g02230 Glyma08g25410 Glyma20g28860 Glyma02g01220 Glyma11g04180 Glyma06g45610 Glyma18g03980 Glyma03g32840 Glyma15g03210 Glyma08g26230 Glyma20g28980 Glyma02g02990 Glyma11g09890 Glyma06g47540 Glyma18g05480 Glyma03g34540 Glyma15g06020 Glyma08g33830 Glyma20g33120 Glyma02g04440 Glyma11g11240 Glyma06g48180 Glyma18g06200 Glyma03g40290 Glyma15g09640 Glyma08g46830 Glyma20g37370 Glyma02g08390 Glyma11g13380 Glyma07g02040 Glyma18g39820 Glyma03g41570 Glyma15g12100 Glyma09g01270 Glyma20g38380 Glyma02g10020 Glyma11g15670 Glyma07g02340 Glyma18g42220 Glyma03g41700 Glyma15g18300 Glyma09g07030 Glyma02g10530 Glyma11g30020 Glyma07g07200 Glyma18g49830

## Module 26

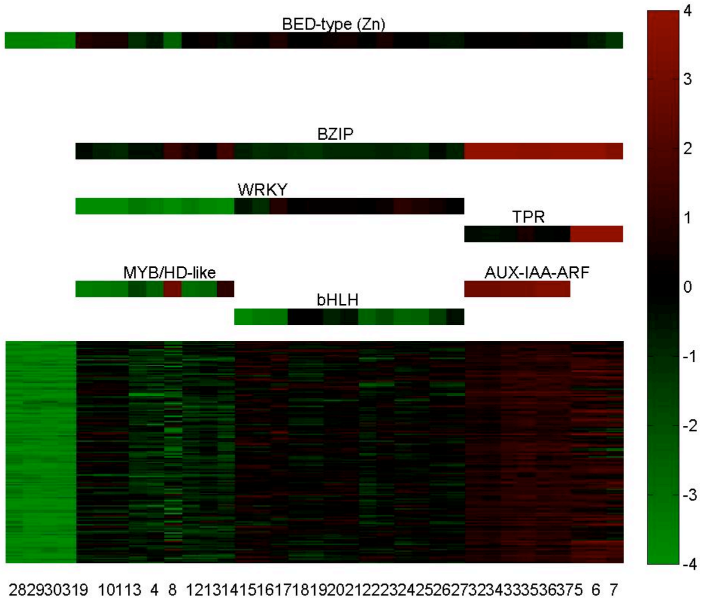


| GO_ACC | GO name | P value |
|---|---|---|
| GO:0048268 | P:clathrin coat assembly | 0.0000316 |
| GO:0009813 | P:flavonoid biosynthetic process | 0.05 |
| GO:0009873 | P:ethylene mediated signaling pathway | 0.03 |
| GO:0015031 | P:protein transport | 0.01 |
| GO:0015937 | P:coenzyme A biosynthetic process | 0.0000128 |
| GO:0015986 | P:ATP synthesis coupled proton transport | 0.00082863 |
| GO:0042742 | P:defense response to bacterium | 0.06 |
| GO:0046686 | P:response to cadmium ion | 0.01 |
| GO:0009651 | P:response to salt stress | 0.06 |
| GO:0046777 | P:protein amino acid autophosphorylation | 0.00082863 |
| GO:0044262 | P:cellular carbohydrate metabolic process | 2.06E-11 |
| GO:0006096 | P:glycolysis | 0.0016621 |
| GO:0006085 | P:acetyl-CoA biosynthetic process | 4.09E-09 |
| GO:0009073 | P:aromatic amino acid family biosynthetic pro... | 0.0000316 |
| GO:0006098 | P:pentose-phosphate shunt | 0.00017139 |
| GO:0006099 | P:tricarboxylic acid cycle | 0.0010432 |
| GO:0006101 | P:citrate metabolic process | 4.72E-08 |
| GO:0006397 | P:mRNA processing | 0.04 |
| GO:0006468 | P:protein amino acid phosphorylation | 0.0000413 |
| GO:0006633 | P:fatty acid biosynthetic process | 0.0002546 |
| GO:0006813 | P:potassium ion transport | 0.00028013 |
| GO:0006897 | P:endocytosis | 0.0010432 |
| GO:0006350 | P:transcription | 0.01 |

| TF | Family |
|---|---|
| Glyma01g02390 | bHLH |
| Glyma02g12830 | WRKY |
| Glyma02g38260 | AUX-IAA-ARF |
| Glyma09g15130 | BED-type (Zn) |
| Glyma13g39330 | BZIP |
| Glyma12g31490 | TPR |
| Glyma12g10790 | MYB/HD-like |

Glyma0048s00280 Glyma11g02030 Glyma06g07140 Glyma15g17060 Glyma03g32260 Glyma13g19500
Glyma09g02800 Glyma18g10260 Glyma01g01900 Glyma11g04960 Glyma06g10650 Glyma15g42080
Glyma03g32910 Glyma13g19860 Glyma09g04000 Glyma18g10270 Glyma01g01920 Glyma11g08350
Glyma06g13410 Glyma15g42300 Glyma03g33370 Glyma13g20790 Glyma09g05100 Glyma18g17240
Glyma01g02350 Glyma11g08550 Glyma06g17840 Glyma15g42690 Glyma03g37470 Glyma13g20810
Glyma09g05490 Glyma18g38610 Glyma01g02850 Glyma11g08560 Glyma06g19320 Glyma15g42900
Glyma03g41860 Glyma13g21030 Glyma09g14660 Glyma18g48170 Glyma01g10340 Glyma11g09350
Glyma06g19960 Glyma15g43040 Glyma04g01220 Glyma13g21360 Glyma09g15620 Glyma18g50950
Glyma01g29470 Glyma11g12580 Glyma06g20490 Glyma16g00590 Glyma04g03150 Glyma13g21550
Glyma09g23980 Glyma18g51260 Glyma01g34780 Glyma11g12630 Glyma06g45800 Glyma16g03350
Glyma04g03840 Glyma13g22970 Glyma09g24180 Glyma18g52040 Glyma01g35190 Glyma11g12920
Glyma07g03930 Glyma16g05470 Glyma04g03870 Glyma13g23960 Glyma09g24890 Glyma19g00900
Glyma01g35200 Glyma11g14360 Glyma07g06790 Glyma16g07400 Glyma04g05250 Glyma13g27250
Glyma09g32640 Glyma19g01400 Glyma01g36070 Glyma11g15680 Glyma07g12600 Glyma16g08080
Glyma04g05580 Glyma13g28120 Glyma09g33160 Glyma19g03030 Glyma01g36740 Glyma11g18290
Glyma07g19660 Glyma16g17580 Glyma04g07050 Glyma13g28710 Glyma09g33630 Glyma19g03040
Glyma01g36750 Glyma11g20040 Glyma07g28600 Glyma16g19780 Glyma04g10820 Glyma13g28880
Glyma09g34620 Glyma19g03090 Glyma01g36890 Glyma11g29360 Glyma07g30210 Glyma16g26210
Glyma04g19010 Glyma13g36540 Glyma09g36000 Glyma19g04850 Glyma01g40330 Glyma12g04790
Glyma07g35110 Glyma16g26220 Glyma04g33060 Glyma13g38480 Glyma09g38220 Glyma19g07140
Glyma01g43470 Glyma12g04830 Glyma07g35910 Glyma16g27350 Glyma04g37220 Glyma13g42420
Glyma10g01480 Glyma19g27360 Glyma02g01450 Glyma12g05050 Glyma07g36840 Glyma16g29450
Glyma04g41440 Glyma14g00320 Glyma10g02040 Glyma19g31050 Glyma02g01920 Glyma12g06280
Glyma07g38070 Glyma16g32070 Glyma04g41850 Glyma14g02380 Glyma10g02740 Glyma19g32260
Glyma02g01930 Glyma12g07780 Glyma0839s00200 Glyma17g02630 Glyma04g43540 Glyma14g03610
Glyma10g02760 Glyma19g35010 Glyma02g07250 Glyma12g08430 Glyma08g00680 Glyma17g03700

| | | | | | |
|---|---|---|---|---|---|
| Glyma05g02740 | Glyma14g04520 | Glyma10g05130 | Glyma19g35620 | Glyma02g07260 | Glyma12g09990 |
| Glyma08g01100 | Glyma17g05290 | Glyma05g03310 | Glyma14g06450 | Glyma10g05500 | Glyma19g36090 |
| Glyma02g08260 | Glyma12g11420 | Glyma08g04770 | Glyma17g05520 | Glyma05g08190 | Glyma14g07710 |
| Glyma10g06590 | Glyma19g40090 | Glyma02g10780 | Glyma12g12740 | Glyma08g07110 | Glyma17g05780 |
| Glyma05g09400 | Glyma14g10510 | Glyma10g06610 | Glyma20g00200 | Glyma02g15070 | Glyma12g16860 |
| Glyma08g08310 | Glyma17g06140 | Glyma05g20150 | Glyma14g11710 | Glyma10g07480 | Glyma20g02980 |
| Glyma02g15400 | Glyma12g20580 | Glyma08g10140 | Glyma17g08120 | Glyma05g24230 | Glyma14g12570 |
| Glyma10g12840 | Glyma20g08040 | Glyma02g16740 | Glyma12g29160 | Glyma08g12200 | Glyma17g09700 |
| Glyma05g25300 | Glyma14g14410 | Glyma10g27660 | Glyma20g12250 | Glyma02g17040 | Glyma12g30660 |
| Glyma08g16200 | Glyma17g11910 | Glyma05g29050 | Glyma14g36410 | Glyma10g30440 | Glyma20g19480 |
| Glyma02g34840 | Glyma12g31970 | Glyma08g16420 | Glyma17g12780 | Glyma05g33050 | Glyma14g36540 |
| Glyma10g32390 | Glyma20g23570 | Glyma02g36560 | Glyma12g32000 | Glyma08g16770 | Glyma17g13440 |
| Glyma05g34490 | Glyma14g40580 | Glyma10g35560 | Glyma20g24100 | Glyma02g38070 | Glyma12g33990 |
| Glyma08g17120 | Glyma17g13900 | Glyma05g34960 | Glyma15g02970 | Glyma10g36160 | Glyma20g25760 |
| Glyma02g42430 | Glyma12g36570 | Glyma08g20060 | Glyma17g16030 | Glyma05g37170 | Glyma15g06590 |
| Glyma10g40490 | Glyma20g26280 | Glyma02g45140 | Glyma13g01870 | Glyma08g23970 | Glyma17g29190 |
| Glyma05g38540 | Glyma15g10160 | Glyma10g40730 | Glyma20g26580 | Glyma02g46380 | Glyma13g04390 |
| Glyma08g27270 | Glyma17g31810 | Glyma06g00200 | Glyma15g10380 | Glyma10g41000 | Glyma20g26840 |
| Glyma02g48160 | Glyma13g05680 | Glyma08g27730 | Glyma17g31940 | Glyma06g00990 | Glyma15g10940 |
| Glyma10g41480 | Glyma20g31460 | Glyma03g07460 | Glyma13g06010 | Glyma08g28230 | Glyma17g33510 |
| Glyma06g01260 | Glyma15g12880 | Glyma10g42540 | Glyma20g31980 | Glyma03g15850 | Glyma13g06230 |
| Glyma08g40380 | Glyma17g34070 | Glyma06g03940 | Glyma15g13680 | Glyma10g42910 | Glyma20g33250 |
| Glyma03g24250 | Glyma13g16540 | Glyma08g43310 | Glyma17g35000 | Glyma06g03970 | Glyma15g15010 |
| Glyma10g43260 | Glyma20g35220 | Glyma03g28320 | Glyma13g16940 | Glyma08g47310 | Glyma17g37260 |
| Glyma06g05350 | Glyma15g15020 | Glyma03g29450 | Glyma13g17220 | Glyma09g01950 | Glyma17g37510 |
| Glyma06g05580 | Glyma15g16810 | | | | |

# Module 30

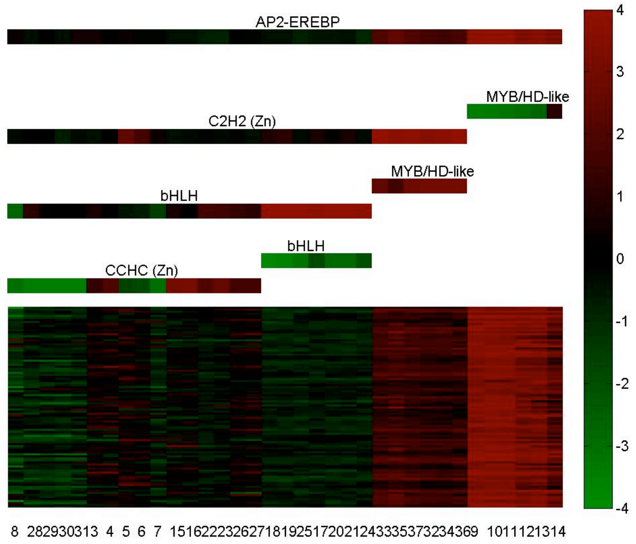


| GO_ACC | GO name | P value |
|---|---|---|
| GO:0009405 | P:pathogenesis | 0.001216 |
| GO:0080001 | P:mucilage extrusion from seed coat | 0.0000154 |
| GO:0051301 | P:cell division | 0.02 |
| GO:0048359 | P:mucilage metabolic process involved seed co... | 0.0000154 |
| GO:0016567 | P:protein ubiquitination | 0.0013804 |
| GO:0008152 | P:metabolic process | 0.02 |
| GO:0006508 | P:proteolysis | 0.02 |
| GO:0006468 | P:protein amino acid phosphorylation | 0.02 |
| GO:0006355 | P:regulation of transcription, DNA-dependent | 0.05 |
| GO:0006350 | P:transcription | 0.0016088 |
| GO:0006089 | P:lactate metabolic process | 0.000000105 |
| GO:0043086 | P:negative regulation of catalytic activity | 0.0000774 |

| TF | Family | Binding site | Gene number |
|---|---|---|---|
| Glyma03g25280 | bHLH | | |
| Glyma11g03300 | MYB/HD-like | | |
| Glyma13g00950 | AP2-EREBP | | |

| | | | |
|---|---|---|---|
| Glyma14g03600 | bHLH | | |
| Glyma14g13360 | C2H2 (Zn) | 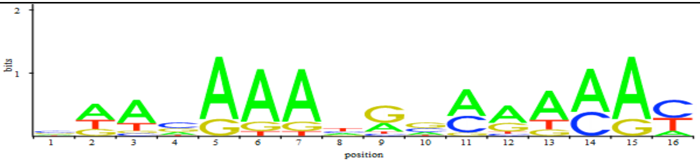 | 40 |
| Glyma19g41150 | CCHC (Zn) | 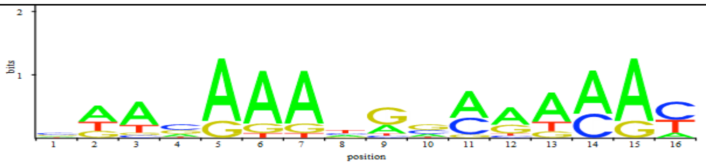 | 40 |
| Glyma12g10790 | MYB/HD-like | | |

Glyma01g02270 Glyma09g24440 Glyma06g04580 Glyma14g11760 Glyma04g04200 Glyma11g23900
Glyma08g06660 Glyma18g11390 Glyma01g02760 Glyma09g26060 Glyma06g07810 Glyma14g25920
Glyma04g04460 Glyma12g14850 Glyma08g07950 Glyma18g31330 Glyma01g05470 Glyma09g28190
Glyma06g08570 Glyma14g33340 Glyma04g07720 Glyma12g17660 Glyma08g10230 Glyma18g31780
Glyma01g30670 Glyma09g32850 Glyma06g09910 Glyma14g35450 Glyma04g09840 Glyma12g26500
Glyma08g10250 Glyma18g45270 Glyma01g44670 Glyma09g33240 Glyma06g11860 Glyma14g36330
Glyma04g12450 Glyma12g26510 Glyma08g10290 Glyma18g49130 Glyma02g01560 Glyma09g33710
Glyma06g17050 Glyma15g11720 Glyma04g35060 Glyma12g30280 Glyma08g12020 Glyma18g49740
Glyma02g11430 Glyma09g36950 Glyma06g17730 Glyma15g11820 Glyma04g37360 Glyma12g32040
Glyma08g14620 Glyma19g31250 Glyma02g11820 Glyma09g37490 Glyma06g19680 Glyma15g11840
Glyma04g38010 Glyma12g36760 Glyma08g20330 Glyma19g39830 Glyma02g37170 Glyma09g40560
Glyma06g35680 Glyma15g16850 Glyma05g02460 Glyma13g00950 Glyma08g24050 Glyma19g40940
Glyma02g44260 Glyma10g01590 Glyma06g48060 Glyma15g29880 Glyma05g03100 Glyma13g02650
Glyma08g24610 Glyma19g43610 Glyma02g45940 Glyma10g04580 Glyma07g00400 Glyma16g01780
Glyma05g06050 Glyma13g03280 Glyma08g28530 Glyma19g45190 Glyma03g01940 Glyma10g05090
Glyma07g00980 Glyma16g02240 Glyma05g24830 Glyma13g05980 Glyma08g37070 Glyma20g12070
Glyma03g06780 Glyma10g05240 Glyma07g05250 Glyma16g05240 Glyma05g27290 Glyma13g07780
Glyma08g45980 Glyma20g21400 Glyma03g20420 Glyma10g07780 Glyma07g05680 Glyma16g31520
Glyma05g27300 Glyma13g18850 Glyma08g45990 Glyma20g29100 Glyma03g20630 Glyma10g12890
Glyma07g08550 Glyma16g31530 Glyma05g28870 Glyma13g19450 Glyma09g00610 Glyma20g30320
Glyma03g24500 Glyma10g32090 Glyma07g13260 Glyma16g31910 Glyma05g31400 Glyma13g19620
Glyma09g00970 Glyma20g30530 Glyma03g28430 Glyma10g35760 Glyma07g30630 Glyma16g32660
Glyma05g33860 Glyma13g21580 Glyma09g00990 Glyma20g30790 Glyma03g28510 Glyma10g36810
Glyma07g33690 Glyma17g01730 Glyma05g35950 Glyma13g37520 Glyma09g05590 Glyma20g31120
Glyma03g37210 Glyma10g37110 Glyma07g38460 Glyma17g07010 Glyma06g00220 Glyma13g38440
Glyma09g18880 Glyma20g31780 Glyma03g38350 Glyma10g37420 Glyma07g39010 Glyma17g09450
Glyma06g00550 Glyma14g04510 Glyma09g22240 Glyma20g35520 Glyma03g40960 Glyma10g38650

Glyma08g03670 Glyma17g13740 Glyma06g04390 Glyma14g08250 Glyma03g42440 Glyma11g00920 Glyma08g05820 Glyma17g36800

# Module 33

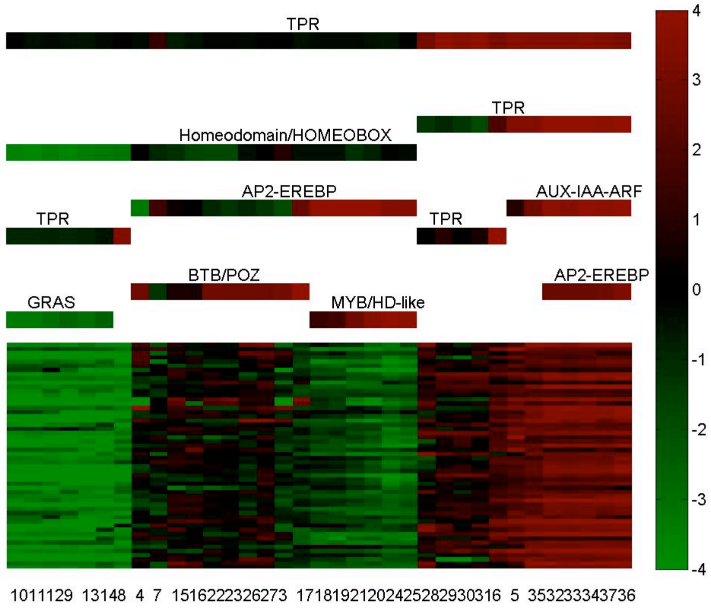


| GO_ACC | GO name | P value |
|---|---|---|
| GO:0006284 | P:base-excision repair | 0.000000124 |
| GO:0006508 | P:proteolysis | 0.00033267 |
| GO:0007047 | P:cellular cell wall organization | 0.0040009 |
| GO:0010623 | P:developmental programmed cell death | 0.000000124 |
| GO:0043086 | P:negative regulation of catalytic activity | 0.00026422 |
| GO:0055114 | P:oxidation reduction | 0.03 |

| TF | Family |
|---|---|
| Glyma01g38360 | GRAS |
| Glyma02g40360 | BTB/POZ |
| Glyma04g04310 | Homeodomain/HOMEOBOX |
| Glyma04g04350 | AP2-EREBP |
| Glyma08g04670 | MYB/HD-like |
| Glyma09g04630 | AP2-EREBP |

| | |
|---|---|
| Glyma13g40310 | AUX-IAA-ARF |
| Glyma14g34640 | TPR |
| Glyma19g01100 | TPR |
| Glyma12g31490 | TPR |

Glyma0022s00480 Glyma10g28010 Glyma06g01730 Glyma14g25480 Glyma03g31310 Glyma13g06050 Glyma08g08550 Glyma18g48490 Glyma01g20740 Glyma10g28020 Glyma06g03410 Glyma14g37440 Glyma03g33720 Glyma13g09420 Glyma08g09350 Glyma18g48530 Glyma01g27710 Glyma10g39470 Glyma06g06870 Glyma15g03040 Glyma04g01630 Glyma13g09430 Glyma08g21410 Glyma18g51480 Glyma01g36780 Glyma10g39690 Glyma06g46530 Glyma15g15360 Glyma04g01640 Glyma13g36930 Glyma08g21730 Glyma19g01100 Glyma01g45440 Glyma11g00280 Glyma07g00540 Glyma15g16900 Glyma04g06780 Glyma13g40000 Glyma08g23860 Glyma19g03500 Glyma02g01090 Glyma11g08520 Glyma07g00900 Glyma16g01650 Glyma04g08830 Glyma13g40210 Glyma08g24750 Glyma19g32450 Glyma02g07960 Glyma11g36620 Glyma07g00910 Glyma16g04770 Glyma04g43510 Glyma13g40960 Glyma08g28580 Glyma19g36470 Glyma02g39320 Glyma12g07400 Glyma07g02060 Glyma16g26630 Glyma05g00830 Glyma13g42330 Glyma09g04330 Glyma20g19200 Glyma02g41950 Glyma12g10240 Glyma07g05150 Glyma16g26970 Glyma05g08530 Glyma13g42340 Glyma09g05630 Glyma20g28070 Glyma03g14450 Glyma12g29670 Glyma07g39540 Glyma17g11090 Glyma05g14800 Glyma14g06970 Glyma10g20470 Glyma20g28290 Glyma03g29620 Glyma12g29790 Glyma08g08540 Glyma18g01730 Glyma05g26440 Glyma14g25340

## Module 34

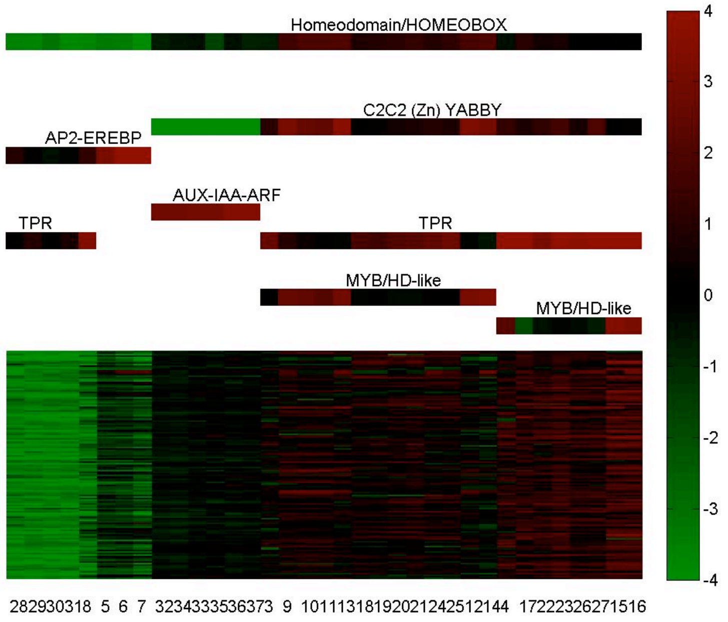

| GO_ACC | GO name | P value |
|---|---|---|
| GO:0006457 | P:protein folding | 0.0013794 |
| GO:0010304 | P:PSII associated light-harvesting complex II... | 0.0000418 |
| GO:0007275 | P:multicellular organismal development | 0.03 |
| GO:0006508 | P:proteolysis | 0.00067788 |
| GO:0006633 | P:fatty acid biosynthetic process | 0.0075904 |

| TF | Family | Binding site | Gene number |
|---|---|---|---|
| Glyma02g38260 | AUX-IAA-ARF | | |
| Glyma06g30000 | TPR | | |
| Glyma06g47000 | MYB/HD-like | | |
| Glyma09g04630 | AP2-EREBP | | |
| Glyma11g06640 | Homeodomain /HOMEOBOX | | |
| Glyma13g22620 | C2C2 (Zn) YABBY | 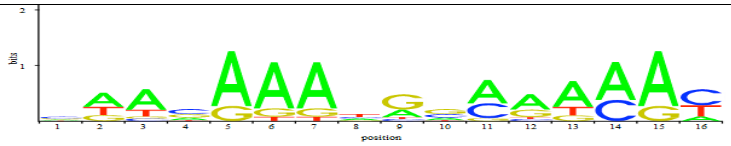 | 63 |
| Glyma17g13780 | MYB/HD-like | | |
| Glyma12g31490 | TPR | | |

Glyma03g30070 Glyma17g37540 Glyma11g04660 Glyma0057s0022 Glyma14g29810 Glyma08g28950 Glyma06g06300Glyma03g31460 Glyma18g00770 Glyma11g06640 Glyma01g06600 Glyma14g38140 Glyma08g36720 Glyma06g06310 Glyma03g32430 Glyma18g01740 Glyma11g15190 Glyma01g11180 Glyma14g40570 Glyma08g45240 Glyma06g12020 Glyma03g35020 Glyma18g05220 Glyma11g20180 Glyma01g12740 Glyma15g01610 Glyma09g01080 Glyma06g13140Glyma03g36910 Glyma18g07380 Glyma11g32010 Glyma01g38650 Glyma15g03840 Glyma09g01980 Glyma06g17360 Glyma03g37810 Glyma18g30900 Glyma11g32880 Glyma01g40650 Glyma15g04840 Glyma09g02450 Glyma06g18400 Glyma03g39570 Glyma18g47900 Glyma11g36870 Glyma01g41230 Glyma15g05240 Glyma09g05820 Glyma06g18510Glyma03g41110 Glyma18g49210 Glyma11g37820 Glyma01g44450 Glyma15g06190

Glyma09g14360 Glyma06g38430 Glyma03g41230 Glyma19g02690 Glyma12g01010 Glyma01g45650
Glyma15g06690 Glyma09g15720 Glyma06g48280 Glyma04g00420 Glyma19g14000 Glyma12g08310
Glyma02g06730 Glyma15g11910 Glyma09g20990 Glyma07g00850 Glyma04g00480 Glyma19g14200
Glyma12g28900 Glyma02g08550 Glyma15g13350 Glyma09g21330 Glyma07g02730 Glyma04g02000
Glyma19g29310 Glyma12g34580 Glyma02g08610 Glyma15g17070 Glyma09g27310 Glyma07g05550
Glyma04g05690 Glyma19g29850 Glyma12g36130 Glyma02g12520 Glyma15g23200 Glyma09g29600
Glyma07g10070 Glyma04g06250 Glyma19g31030 Glyma12g36530 Glyma02g15640 Glyma15g39770
Glyma09g36340 Glyma07g12660 Glyma04g13640 Glyma19g31120 Glyma13g05440 Glyma02g15680
Glyma15g40810 Glyma09g37020 Glyma07g22950 Glyma04g32810 Glyma19g32930 Glyma13g07920
Glyma02g16100 Glyma15g42530 Glyma09g37450 Glyma07g32760 Glyma04g36390 Glyma19g32940
Glyma13g08160 Glyma02g16210 Glyma15g43210 Glyma10g02240 Glyma07g32790 Glyma04g36460
Glyma19g34300 Glyma13g16460 Glyma02g26820 Glyma16g00410 Glyma10g03590 Glyma07g35310
Glyma04g37740 Glyma19g35160 Glyma13g17290 Glyma02g35780 Glyma16g02090 Glyma10g04980
Glyma07g38340 Glyma04g40550 Glyma19g37750 Glyma13g19320 Glyma02g39990 Glyma16g32360
Glyma10g09470 Glyma07g38710 Glyma04g42720 Glyma19g39560 Glyma13g23260 Glyma02g45880
Glyma16g34180 Glyma10g09550 Glyma07g40020 Glyma05g01090 Glyma19g40410 Glyma13g27220
Glyma02g47460 Glyma17g00790 Glyma10g11680 Glyma08g00380 Glyma05g02840 Glyma19g42180
Glyma13g32640 Glyma03g00840 Glyma17g01990 Glyma10g20450 Glyma08g01510 Glyma05g07670
Glyma19g43750 Glyma13g33210 Glyma03g01990 Glyma17g02420 Glyma10g28100 Glyma08g04590
Glyma05g25810 Glyma19g43850 Glyma13g35950 Glyma03g02000 Glyma17g05200 Glyma10g30400
Glyma08g08770 Glyma05g25870 Glyma20g03310 Glyma13g40560 Glyma03g02540 Glyma17g05980
Glyma10g31370 Glyma08g10730 Glyma05g27760 Glyma20g13780 Glyma13g41560 Glyma03g24280
Glyma17g06200 Glyma10g35610 Glyma08g13100 Glyma05g29990 Glyma20g22120 Glyma13g43730
Glyma03g27770 Glyma17g09160 Glyma10g40390 Glyma08g18150 Glyma05g32720 Glyma20g26270
Glyma13g44550 Glyma03g27780 Glyma17g10810 Glyma10g41010 Glyma08g19780 Glyma05g35120
Glyma20g26950 Glyma14g01290 Glyma03g28290 Glyma17g11570 Glyma10g44560 Glyma08g22130
Glyma05g38060 Glyma20g31940 Glyma14g02880 Glyma03g28410 Glyma17g12840 Glyma11g01080
Glyma08g23250 Glyma06g00510 Glyma20g34880 Glyma14g07690 Glyma03g29720 Glyma17g13510
Glyma11g04180 Glyma08g23800 Glyma06g00580 Glyma20g36140 Glyma14g11210 Glyma06g01780
Glyma20g36740 Glyma14g11730 Glyma06g02100 Glyma20g39340 Glyma14g24600

# Module 37

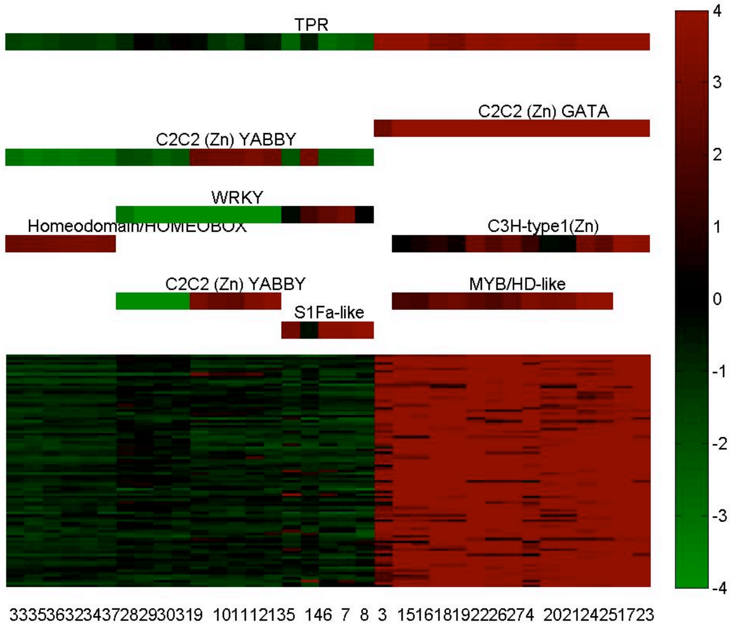


| GO_ACC | GO name | P value |
|---|---|---|
| GO:0015979 | P:photosynthesis | 0.02 |
| GO:0045454 | P:cell redox homeostasis | 0.0055152 |
| GO:0045449 | P:regulation of transcription | 0.0086677 |
| GO:0009409 | P:response to cold | 0.0000476 |
| GO:0006350 | P:transcription | 0.0066199 |
| GO:0055114 | P:oxidation reduction | 0.01 |
| GO:0009909 | P:regulation of flower development | 0.000000563 |

| TF | Family | Binding site | Gene number |
|---|---|---|---|
| Glyma04g24430 | TPR | | |
| Glyma09g41050 | WRKY | | |
| Glyma10g05790 | S1Fa-like | | |
| Glyma12 | MYB/HD-like | | |

| | | | |
|---|---|---|---|
| g33430 | | | |
| Glyma13 g22620 | C2C2 (Zn) YABBY | 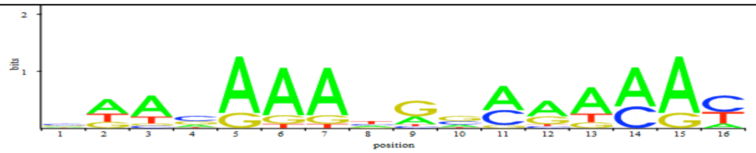 | 39 |
| Glyma14 g09310 | Homeodomain /HOMEOBOX | | |
| Glyma14 g10830 | C2C2 (Zn) GATA | | |
| Glyma17 g14710 | C2C2 (Zn) YABBY | 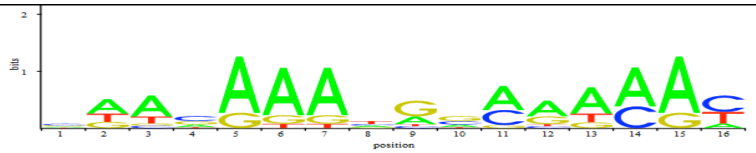 | 39 |
| Glyma18 g09020 | C3H-type1(Zn) | | |

Glyma01g09930 Glyma08g28370 Glyma04g42380 Glyma14g05560 Glyma03g26540 Glyma11g17120
Glyma06g18720 Glyma18g14490 Glyma01g11640 Glyma08g35480 Glyma05g08590 Glyma14g05660
Glyma03g27320 Glyma11g29880 Glyma06g19720 Glyma18g19050 Glyma01g11870 Glyma08g37270
Glyma05g09060 Glyma14g34080 Glyma03g28360 Glyma11g37450 Glyma06g42180 Glyma18g27710
Glyma01g15930 Glyma09g35030 Glyma05g09070 Glyma14g38640 Glyma03g38490 Glyma12g05840
Glyma0737s0021 Glyma18g40100 Glyma01g35460 Glyma09g36640 Glyma05g27080 Glyma15g02080
Glyma03g39020 Glyma12g13020 Glyma07g02610 Glyma18g51320 Glyma01g43030 Glyma10g03200
Glyma05g38490 Glyma15g06270 Glyma04g03430 Glyma12g16250 Glyma07g03360 Glyma19g00580
Glyma02g16620 Glyma10g36990 Glyma06g03520 Glyma15g15170 Glyma04g03580 Glyma13g07030
Glyma07g08150 Glyma19g00590 Glyma02g37990 Glyma10g40700 Glyma06g06900 Glyma15g17340
Glyma04g06970 Glyma13g15560 Glyma07g15190 Glyma19g01050 Glyma02g40360 Glyma10g42100
Glyma06g12420 Glyma16g10880 Glyma04g33610 Glyma13g42960 Glyma07g23800 Glyma19g30290
Glyma02g43340 Glyma10g43690 Glyma06g12710 Glyma16g15790 Glyma04g35030 Glyma13g43260
Glyma07g38820 Glyma19g41090 Glyma02g43350 Glyma11g02460 Glyma06g12720 Glyma16g29640
Glyma04g39650 Glyma14g01470 Glyma08g01180 Glyma19g41590 Glyma02g45450 Glyma11g06490
Glyma06g14880 Glyma17g01900 Glyma04g39970 Glyma14g01670 Glyma08g07310 Glyma20g23100
Glyma02g47280 Glyma11g12960 Glyma06g15220 Glyma17g02670 Glyma04g42070 Glyma14g03300
Glyma08g10060 Glyma20g26560 Glyma03g01720 Glyma11g13870 Glyma06g17520 Glyma17g16510
Glyma04g42080 Glyma14g03360 Glyma08g22740 Glyma20g26640 Glyma08g23400 Glyma20g38370

# Module 38

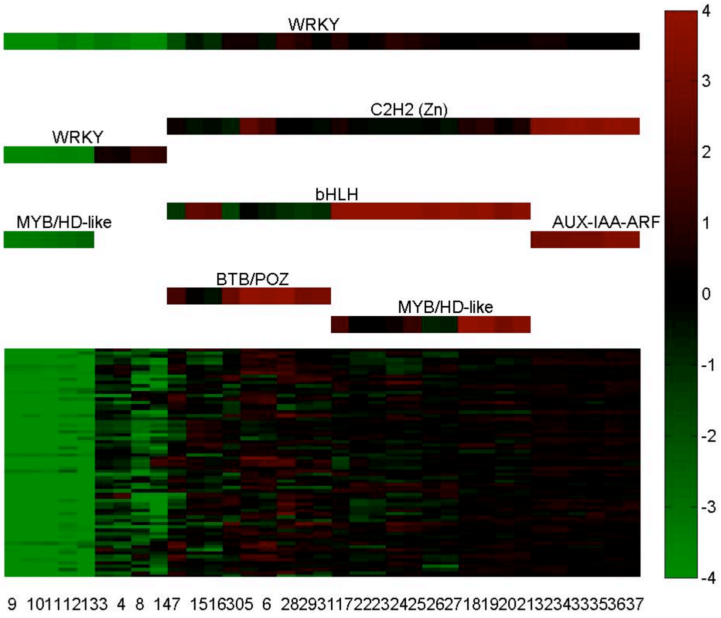


| GO_ACC | GO name | P value |
|---|---|---|
| GO:0006508 | P:proteolysis | 0.0073806 |
| GO:0009813 | P:flavonoid biosynthetic process | 0.0068524 |
| GO:0045449 | P:regulation of transcription | 0.0098571 |

| TF | Family | Binding site | Gene number |
|---|---|---|---|
| Glyma02 g12830 | WRKY | | |
| Glyma02 g38260 | AUX-IAA-ARF | | |
| Glyma09 g41050 | WRKY | | |
| Glyma11 g14490 | MYB/HD -like | 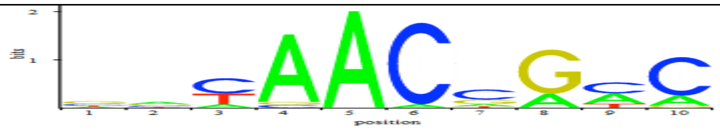 | 10 |

| | | | |
|---|---|---|---|
| Glyma11g17120 | bHLH | | |
| Glyma14g13360 | C2H2 (Zn) | 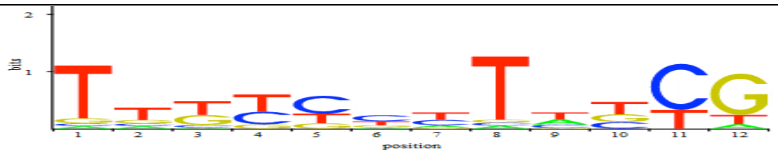 | 32 |
| Glyma18g06680 | BTB/POZ | | |
| Glyma12g10790 | MYB/HD-like | 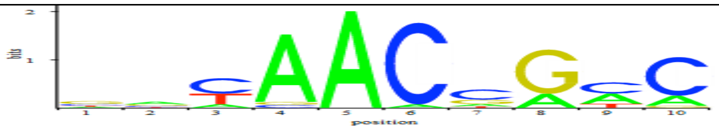 | 10 |

Glyma01g06870 Glyma11g03690 Glyma06g04350 Glyma17g14930 Glyma03g24300 Glyma13g44170
Glyma08g26150 Glyma18g07980 Glyma01g31750 Glyma11g08530 Glyma06g09470 Glyma17g17540
Glyma03g32130 Glyma14g01330 Glyma08g27720 Glyma18g08000 Glyma01g36770 Glyma11g33280
Glyma06g09520 Glyma17g18800 Glyma04g02750 Glyma14g37050 Glyma08g40620 Glyma18g50940
Glyma02g12830 Glyma11g33560 Glyma06g17500 Glyma17g34570 Glyma04g04170 Glyma15g09750
Glyma08g43330 Glyma19g00980 Glyma02g13730 Glyma12g30310 Glyma06g19920 Glyma17g34590
Glyma04g09310 Glyma15g19580 Glyma08g44930 Glyma19g34890 Glyma02g26980
Glyma1337s00200 Glyma06g26370 Glyma17g34870Glyma04g09380 Glyma15g32800 Glyma09g08100
Glyma20g01580 Glyma02g35630 Glyma13g28090 Glyma07g04080 Glyma17g35720 Glyma04g40810
Glyma16g01060 Glyma10g13700 Glyma20g16630 Glyma02g39000 Glyma13g29320 Glyma07g04470
Glyma17g36400 Glyma05g08650 Glyma16g07750 Glyma10g28560 Glyma20g26400 Glyma02g40290
Glyma13g39580 Glyma07g04500 Glyma18g04660 Glyma05g22120 Glyma16g17190 Glyma10g36950
Glyma20g30650 Glyma02g47420 Glyma13g42330 Glyma07g36150 Glyma18g07680 Glyma06g02770
Glyma17g05670 Glyma10g40910

# Module 41

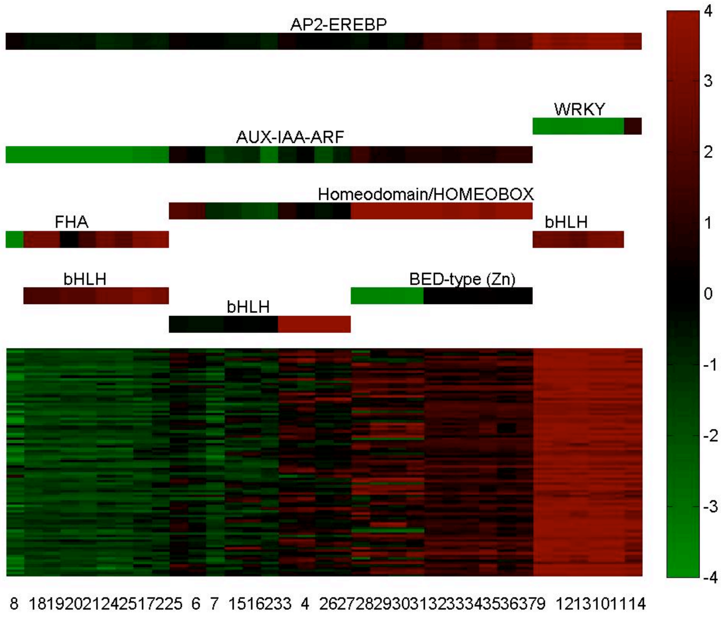


| GO_ACC | GO name | P value |
|---|---|---|
| GO:0006952 | P:defense response | 0.0042377 |
| GO:0009873 | P:ethylene mediated signaling pathway | 0.0091965 |
| GO:0055114 | P:oxidation reduction | 0.0000865 |
| GO:0006950 | P:response to stress | 0.07 |
| GO:0006355 | P:regulation of transcription, DNA-dependent | 0.01 |
| GO:0005975 | P:carbohydrate metabolic process | 0.000016 |
| GO:0006350 | P:transcription | 0.0083308 |

| TF | Family |
|---|---|
| Glyma02g42780 | bHLH |
| Glyma03g32740 | bHLH |
| Glyma04g04310 | Homeodomain/HOMEOBOX |
| Glyma08g37070 | AUX-IAA-ARF |
| Glyma09g15130 | BED-type (Zn) |
| Glyma09g41050 | WRKY |
| Glyma13g00950 | AP2-EREBP |

| | |
|---|---|
| Glyma17g20020 | FHA |
| Glyma19g27480 | bHLH |

Glyma01g02760 Glyma09g24700 Glyma05g37330 Glyma14g23560 Glyma03g39140 Glyma12g07960 Glyma07g39010 Glyma17g10730 Glyma01g35960 Glyma09g26060 Glyma06g02240 Glyma14g34070 Glyma03g42440 Glyma12g09120 Glyma07g39140 Glyma17g13790 Glyma02g01560 Glyma09g30930 Glyma06g04390 Glyma14g36860 Glyma04g02140 Glyma12g11150 Glyma07g39960 Glyma17g16330 Glyma02g06800 Glyma09g33240 Glyma06g04530 Glyma14g37350 Glyma04g02150 Glyma12g26510 Glyma08g03260 Glyma17g18880 Glyma02g08410 Glyma09g40990 Glyma06g06670 Glyma14g39510 Glyma04g04200 Glyma12g36540 Glyma08g03670 Glyma17g36490 Glyma02g17140 Glyma10g01030 Glyma06g11860 Glyma15g06290 Glyma04g06590 Glyma13g00950 Glyma08g04810 Glyma18g10080 Glyma02g29340 Glyma10g01050 Glyma06g35680 Glyma15g14330 Glyma04g07000 Glyma13g03200 Glyma08g08970 Glyma18g15380 Glyma02g38750 Glyma10g01590 Glyma06g36730 Glyma16g02240 Glyma05g01160 Glyma13g06080 Glyma08g10640 Glyma18g44820 Glyma02g39230 Glyma10g02650 Glyma06g41520 Glyma16g02440 Glyma05g03190 Glyma13g27230 Glyma08g10930 Glyma19g03530 Glyma02g41120 Glyma10g29550 Glyma06g45680 Glyma16g17830 Glyma05g03210 Glyma13g33070 Glyma08g11240 Glyma19g29670 Glyma02g41190 Glyma10g30650 Glyma07g00400 Glyma16g23180 Glyma05g14700 Glyma13g38130 Glyma08g12020 Glyma19g36630 Glyma02g42020 Glyma10g33410 Glyma07g05680 Glyma16g25830 Glyma05g27650 Glyma13g44870 Glyma08g14750 Glyma19g41700 Glyma02g44260 Glyma10g39760 Glyma07g07680 Glyma16g31910 Glyma05g28260 Glyma14g01110 Glyma08g17470 Glyma19g42010 Glyma02g45940 Glyma10g41500 Glyma07g11230 Glyma16g34640 Glyma05g28870 Glyma14g02180 Glyma08g24050 Glyma19g45190 Glyma02g46450 Glyma11g06330 Glyma07g11300 Glyma17g01600 Glyma05g31520 Glyma14g02200 Glyma08g42490 Glyma20g08190 Glyma02g46480 Glyma11g09470 Glyma07g13020 Glyma17g01730 Glyma05g34900 Glyma14g04510 Glyma08g43460 Glyma20g21980 Glyma02g47570 Glyma11g14590 Glyma07g14620 Glyma17g03050 Glyma05g35950 Glyma14g06880 Glyma09g03400 Glyma20g27970 Glyma03g00980 Glyma11g14970 Glyma07g37200 Glyma17g05350 Glyma05g36340 Glyma14g08630 Glyma09g14140 Glyma20g36960 Glyma03g01130 Glyma11g15490 Glyma07g37600 Glyma17g07010 Glyma20g37760 Glyma03g33910 Glyma12g06950 Glyma07g38460 Glyma17g08020

# Module 48

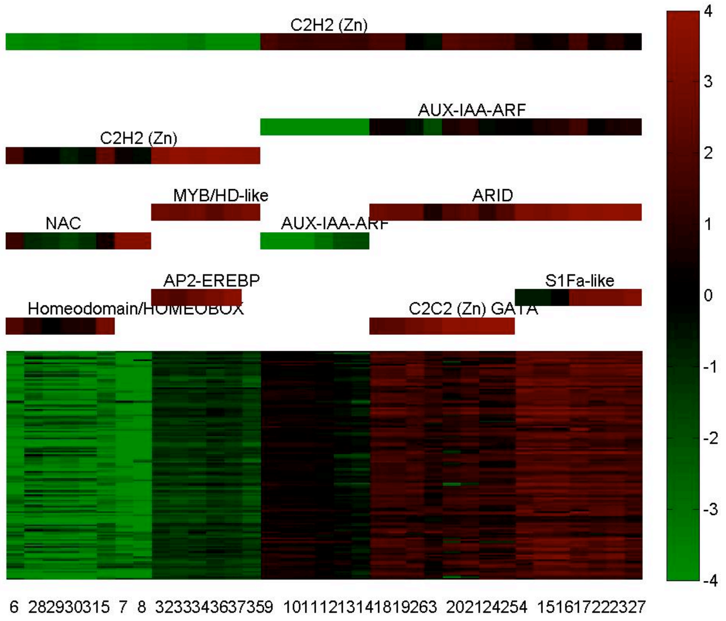


| GO_ACC | GO name | P value |
|---|---|---|
| GO:0015995 | P:chlorophyll biosynthetic process | 0.00068924 |
| GO:0009765 | P:photosynthesis, light harvesting | 1.22E-11 |
| GO:0022900 | P:electron transport chain | 0.0074011 |
| GO:0019853 | P:L-ascorbic acid biosynthetic process | 0.00011224 |
| GO:0019253 | P:reductive pentose-phosphate cycle | 9.53E-12 |
| GO:0018298 | P:protein-chromophore linkage | 2.16E-11 |
| GO:0006412 | P:translation | 0.00000685 |
| GO:0009773 | P:photosynthetic electron transport in photos... | 0.0000708 |
| GO:0006810 | P:transport | 0.00151 |
| GO:0009853 | P:photorespiration | 0.0000409 |
| GO:0015979 | P:photosynthesis | 1.62E-11 |

| TF | Family | Binding site | Gene number |
|---|---|---|---|
| Glyma03 g00980 | MYB/HD-like | 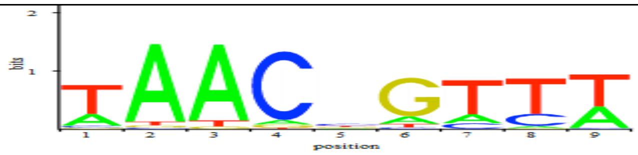 | 10 |
| Glyma04 g02210 | ARID | | |

| | | | |
|---|---|---|---|
| Glyma07 g39350 | Homeodomain /HOM | | |
| Glyma10 g05790 | S1Fa-like | | |
| Glyma10 g32340 | AUX-IAA-ARF | | |
| Glyma13 g35550 | NAC | | |
| Glyma13 g40240 | C2H2 (Zn) | 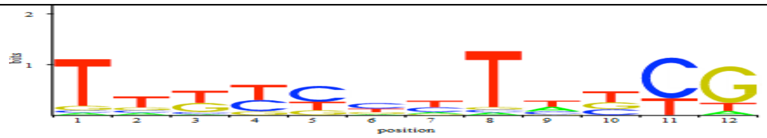 | 25 |
| Glyma14 g13360 | C2H2 (Zn) | 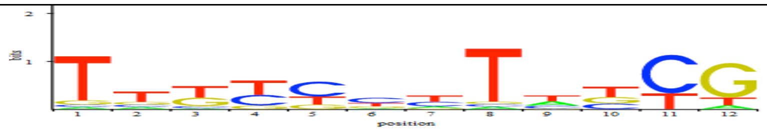 | 25 |
| Glyma15 g02040 | AUX-IAA-ARF | | |
| Glyma17 g06290 | C2C2 (Zn) GATA | | |
| Glyma19 g29000 | AP2-EREBP | | |

Glyma0066s00240 Glyma09g38470 Glyma04g39370 Glyma15g22780 Glyma02g45190
Glyma12g13030 Glyma07g05320 Glyma17g23060 Glyma01g01480 Glyma09g38610 Glyma04g39800
Glyma15g24680 Glyma02g47560 Glyma12g32580 Glyma07g06660 Glyma18g04080 Glyma01g01520
Glyma10g02930 Glyma04g40680 Glyma15g39780 Glyma02g47960 Glyma12g34400 Glyma07g14340
Glyma18g05600 Glyma01g06740 Glyma10g03750 Glyma04g42840 Glyma15g40450 Glyma03g08280
Glyma12g34770 Glyma07g29000 Glyma18g05620 Glyma01g08550 Glyma10g04790 Glyma05g00620
Glyma15g41540 Glyma03g08290 Glyma12g35050 Glyma07g31830 Glyma18g43800 Glyma01g28400
Glyma10g29240 Glyma05g01000 Glyma16g01870 Glyma03g26740 Glyma12g36130 Glyma08g00490
Glyma18g47710 Glyma01g28810 Glyma10g32080 Glyma05g03730 Glyma16g03230 Glyma03g27380
Glyma13g07610 Glyma08g07880 Glyma18g47850 Glyma01g34700 Glyma10g35950 Glyma05g09270
Glyma16g04940 Glyma03g27510 Glyma13g10340 Glyma08g08770 Glyma19g00820 Glyma01g37050
Glyma10g39460 Glyma05g23690 Glyma16g14720 Glyma03g29330 Glyma13g20830 Glyma08g13360
Glyma19g06340 Glyma01g37900 Glyma10g40900 Glyma05g24660 Glyma16g24870 Glyma03g32430

Glyma13g24640 Glyma08g13790 Glyma19g06370 Glyma01g38750 Glyma11g00300 Glyma05g28210
Glyma16g25770 Glyma03g37800 Glyma13g30380 Glyma08g14350 Glyma19g06420 Glyma01g41400
Glyma11g04020 Glyma05g30600 Glyma16g25860 Glyma03g38550 Glyma13g35800 Glyma08g15090
Glyma19g28240 Glyma01g45430 Glyma11g06510 Glyma05g31160 Glyma16g26130 Glyma03g40720
Glyma13g36150 Glyma08g17610 Glyma19g30350 Glyma02g06100 Glyma11g08230 Glyma06g02300
Glyma16g26620 Glyma03g41420 Glyma13g37880 Glyma08g18510 Glyma19g32070 Glyma02g06730
Glyma11g12000 Glyma06g02710 Glyma16g27990 Glyma03g42310 Glyma13g43370 Glyma08g19210
Glyma19g35160 Glyma02g06830 Glyma11g17480 Glyma06g05810 Glyma16g28030 Glyma04g02240
Glyma14g00640 Glyma08g21900 Glyma19g40400 Glyma02g07180 Glyma11g18640 Glyma06g07110
Glyma16g28070 Glyma04g02680 Glyma14g01130 Glyma08g23450 Glyma19g41150 Glyma02g08910
Glyma11g19400 Glyma06g11920 Glyma16g28310 Glyma04g05800 Glyma14g03560 Glyma08g28740
Glyma19g43400 Glyma02g09680 Glyma11g31660 Glyma06g14120 Glyma16g33020 Glyma04g07010
Glyma14g07270 Glyma09g07310 Glyma19g43410 Glyma02g12680 Glyma11g31680 Glyma06g15070
Glyma16g33030 Glyma04g11680 Glyma14g15810 Glyma09g11460 Glyma19g44010 Glyma02g16000
Glyma11g32450 Glyma06g15540 Glyma17g08310 Glyma04g11910 Glyma15g01940 Glyma09g13220
Glyma20g28300 Glyma02g16850 Glyma11g34230 Glyma06g20960 Glyma17g10890 Glyma04g12510
Glyma15g05790 Glyma09g28200 Glyma20g31640 Glyma02g34810 Glyma11g37440 Glyma06g48030
Glyma17g14240 Glyma04g33360 Glyma15g08820 Glyma09g32600 Glyma20g35530 Glyma02g36370
Glyma12g09100 Glyma07g02240 Glyma17g16610 Glyma04g34230 Glyma15g12130 Glyma09g34280
Glyma20g38040 Glyma02g41700 Glyma12g09730 Glyma07g02570 Glyma17g17040

## Module 50

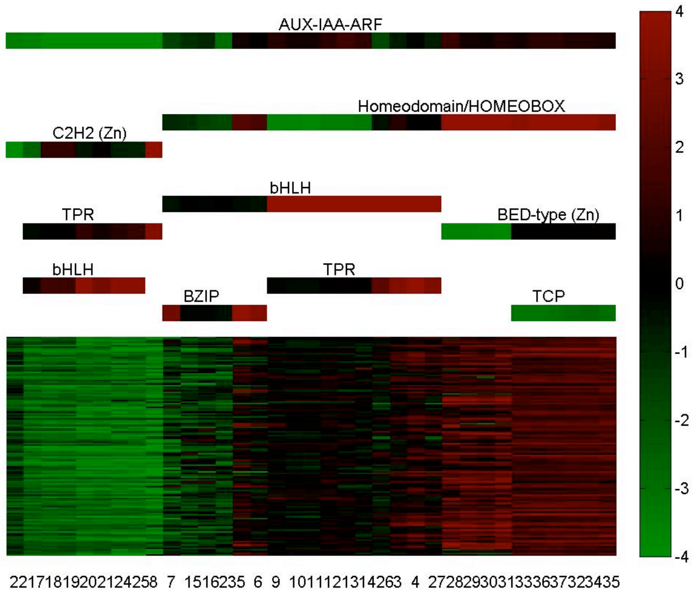


| GO_ACC | GO name | P value |
|---|---|---|
| GO:0006979 | P:response to oxidative stress | 0.0007061 |

| | | |
|---|---|---|
| GO:0055085 | P:transmembrane transport | 0.03 |
| GO:0051258 | P:protein polymerization | 0.00093421 |
| GO:0046274 | P:lignin catabolic process | 0.0027726 |
| GO:0009809 | P:lignin biosynthetic process | 0.0014746 |
| GO:0016042 | P:lipid catabolic process | 0.05 |
| GO:0055114 | P:oxidation reduction | 0.02 |
| GO:0009926 | P:auxin polar transport | 0.0000366 |
| GO:0007586 | P:digestion | 0.0015731 |
| GO:0007049 | P:cell cycle | 0.03 |
| GO:0007018 | P:microtubule-based movement | 0.0035362 |
| GO:0006869 | P:lipid transport | 0.03 |
| GO:0006334 | P:nucleosome assembly | 0.00049032 |
| GO:0006270 | P:DNA replication initiation | 0.000000113 |
| GO:0009734 | P:auxin mediated signaling pathway | 0.06 |
| GO:0007047 | P:cellular cell wall organization | 0.0010631 |

| TF | Family | Binding site | Gene number |
|---|---|---|---|
| Glyma02 g42780 | bHLH | | |
| Glyma04 g04310 | Homeodomain /HOMEOBOX | | |
| Glyma06 g30000 | TPR | | |
| Glyma08 g37070 | AUX-IAA-ARF | | |
| Glyma09 g15130 | BED-type (Zn) | 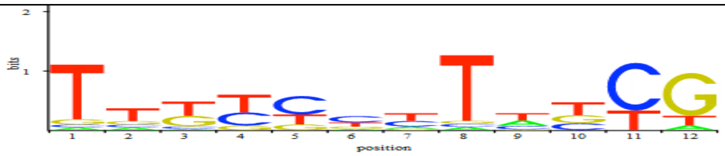 | 50 |
| Glyma13 g34530 | C2H2 (Zn) | 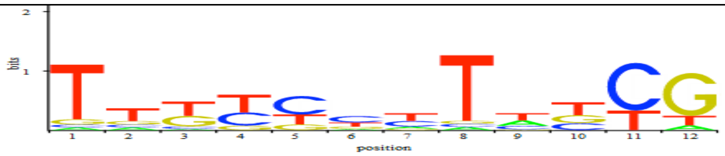 | 50 |
| Glyma13 | TCP | | |

| | | | |
|---|---|---|---|
| g34690 | | | |
| Glyma15 g18580 | bHLH | | |
| Glyma12 g30990 | BZIP | 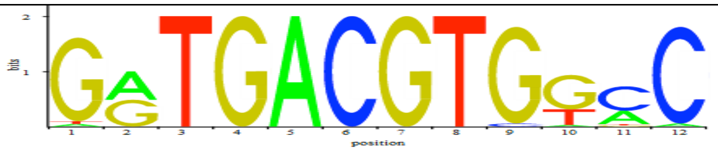 | 17 |
| Glyma12 g31490 | TPR | | |

Glyma01g00540 Glyma09g38320 Glyma05g28470 Glyma14g08190 Glyma03g31880 Glyma11g19490 Glyma07g15580 Glyma17g17970 Glyma01g01310 Glyma10g00250 Glyma05g32190 Glyma14g08200 Glyma03g34080 Glyma12g00890 Glyma07g17490 Glyma17g34020 Glyma01g03880 Glyma10g03640 Glyma05g32200 Glyma14g11780 Glyma03g34750 Glyma12g05910 Glyma07g40150 Glyma17g38120 Glyma01g37430 Glyma10g06220 Glyma06g02650 Glyma14g36030 Glyma03g35510 Glyma12g07690 Glyma08g07890 Glyma17g38210 Glyma01g38980 Glyma10g11620 Glyma06g02760 Glyma14g39760 Glyma03g40280 Glyma12g08990 Glyma08g13110 Glyma18g07330 Glyma01g39090 Glyma10g24630 Glyma06g04660 Glyma14g39880 Glyma03g41880 Glyma12g18230 Glyma08g17010 Glyma18g48030 Glyma01g39720 Glyma10g24660 Glyma06g05470 Glyma15g05480 Glyma03g41990 Glyma12g35030 Glyma08g18080 Glyma18g49400 Glyma01g39810 Glyma10g27960 Glyma06g06200 Glyma15g11890 Glyma0404s00200Glyma12g35820Glyma08g19570 Glyma19g02150 Glyma01g39990 Glyma10g32160 Glyma06g06230 Glyma15g13640 Glyma04g02740 Glyma13g05810 Glyma08g21390 Glyma19g29720 Glyma01g42450 Glyma10g32860 Glyma06g06250 Glyma15g16570 Glyma04g05420 Glyma13g22420 Glyma08g22470 Glyma19g32210 Glyma01g42950 Glyma10g38900 Glyma06g06280 Glyma15g22810 Glyma04g06750 Glyma13g23200 Glyma08g46090 Glyma19g33360 Glyma02g00930 Glyma10g40150 Glyma06g06840 Glyma15g34870 Glyma04g08100 Glyma13g26990 Glyma08g48310 Glyma19g33920 Glyma02g03810 Glyma10g43420 Glyma06g08180 Glyma15g38040 Glyma04g09350 Glyma13g33380 Glyma09g01050 Glyma19g34090 Glyma02g05480 Glyma11g02530 Glyma06g10980 Glyma15g42140 Glyma04g11240 Glyma13g33480 Glyma09g02750 Glyma19g34620 Glyma02g12460 Glyma11g02950 Glyma06g16420 Glyma15g43040 Glyma04g32130 Glyma13g33620 Glyma09g03300 Glyma19g36820 Glyma02g12800 Glyma11g05300 Glyma06g19480 Glyma15g43180 Glyma04g34150 Glyma13g33650 Glyma09g05240 Glyma19g42890 Glyma02g37800 Glyma11g05470 Glyma06g22410 Glyma16g02380 Glyma04g35360 Glyma13g34580 Glyma09g08120 Glyma19g44540 Glyma02g41590 Glyma11g05560 Glyma06g22930 Glyma16g04730 Glyma04g38590 Glyma13g35050 Glyma09g15620 Glyma19g44680 Glyma02g47790 Glyma11g06170 Glyma06g26810 Glyma16g13610 Glyma04g40580 Glyma13g35510 Glyma09g17800 Glyma20g23400 Glyma03g05440 Glyma11g06290 Glyma06g32860 Glyma16g23900 Glyma04g40590 Glyma14g00830 Glyma09g26460 Glyma20g25240 Glyma03g22620 Glyma11g07850 Glyma07g03620 Glyma16g28080 Glyma05g00370 Glyma14g05250 Glyma09g30700 Glyma20g27280

Glyma03g29760 Glyma11g09920 Glyma07g10810 Glyma17g11220 Glyma05g09290 Glyma14g05270 Glyma09g31260 Glyma20g29840 Glyma03g31060 Glyma11g13940 Glyma07g10820 Glyma17g11630 Glyma05g21820 Glyma14g05290 Glyma09g37290 Glyma20g34780 Glyma03g31240 Glyma11g15780 Glyma07g11550 Glyma17g15990 Glyma05g24730 Glyma14g07360

# Module 56

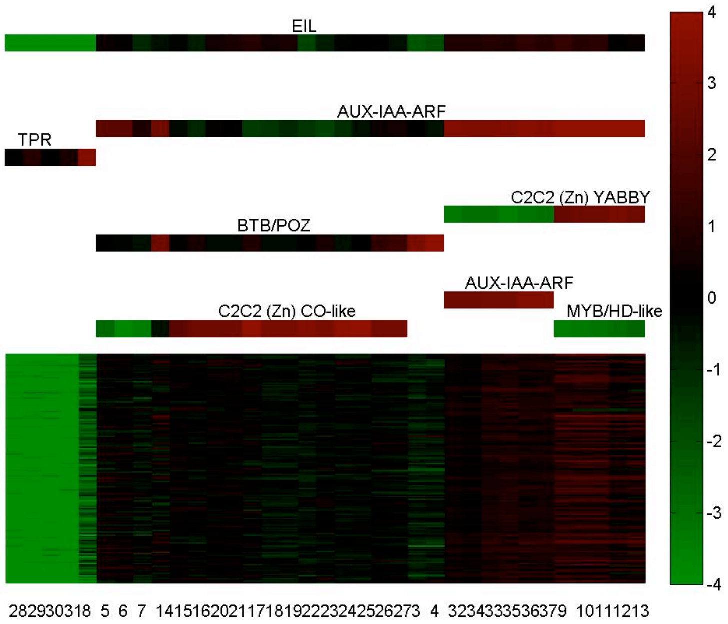


| GO_ACC | GO name | P value |
|---|---|---|
| GO:0006511 | P:ubiquitin-dependent protein catabolic process | 0.000072 |
| GO:0001666 | P:response to hypoxia | 0.000038 |
| GO:0006096 | P:glycolysis | 0.01 |
| GO:0006289 | P:nucleotide-excision repair | 0.000038 |
| GO:0006397 | P:mRNA processing | 7.74E-10 |
| GO:0006412 | P:translation | 0.0000352 |
| GO:0006414 | P:translational elongation | 0.000025 |
| GO:0051028 | P:mRNA transport | 0.000012 |
| GO:0006417 | P:regulation of translation | 3.06E-08 |
| GO:0051246 | P:regulation of protein metabolic process | 0.00012997 |
| GO:0048510 | P:regulation of timing of transition from veg... | 1.92E-08 |
| GO:0046686 | P:response to cadmium ion | 0.01 |
| GO:0043161 | P:proteasomal ubiquitin-dependent protein cat... | 0.0000751 |
| GO:0031396 | P:regulation of protein ubiquitination | 0.00000389 |
| GO:0030163 | P:protein catabolic process | 0.0000198 |
| GO:0030154 | P:cell differentiation | 0.00000215 |

| | | |
|---|---|---|
| GO:0016192 | P:vesicle-mediated transport | 0.00000052 |
| GO:0015986 | P:ATP synthesis coupled proton transport | 0.00099022 |
| GO:0015031 | P:protein transport | 0.00058116 |
| GO:0010321 | P:regulation of vegetative phase change | 9.81E-10 |
| GO:0008152 | P:metabolic process | 0.04 |
| GO:0055114 | P:oxidation reduction | 0.99 |
| GO:0006730 | P:one-carbon metabolic process | 0.0000271 |
| GO:0006457 | P:protein folding | 0.06 |
| GO:0010228 | P:vegetative to reproductive phase transition... | 4.31E-11 |
| GO:0006915 | P:apoptosis | 0.02 |
| GO:0008380 | P:RNA splicing | 0.0012019 |
| GO:0009611 | P:response to wounding | 0.000616 |
| GO:0009651 | P:response to salt stress | 0.07 |
| GO:0009908 | P:flower development | 0.000000112 |
| GO:0009910 | P:negative regulation of flower development | 5.57E-09 |
| GO:0010048 | P:vernalization response | 5.57E-09 |
| GO:0006886 | P:intracellular protein transport | 0.000000204 |

| TF | Family | Binding site | Gene number |
|---|---|---|---|
| Glyma02g38260 | AUX-IAA-ARF | | |
| Glyma02g44220 | EIL | | |
| Glyma13g43050 | AUX-IAA-ARF | | |
| Glyma15g03400 | C2C2 (Zn) CO-like | | |
| Glyma17g14710 | C2C2 (Zn) YABBY | 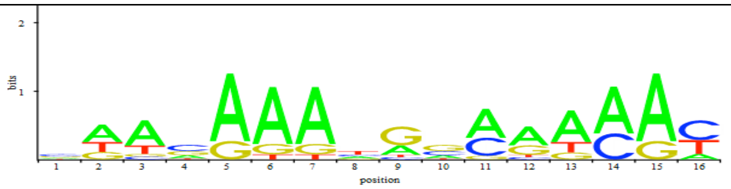 | 58 |
| Glyma17g17440 | BTB/POZ | | |
| Glyma12g31490 | TPR | | |

| | | | |
|---|---|---|---|
| Glyma12g10790 | MYB/HD-like | | |

Glyma01g01460 Glyma10g01210 Glyma05g28790 Glyma14g17230 Glyma03g01920 Glyma12g06020 Glyma08g05030 Glyma18g01870 Glyma01g03540 Glyma10g01820 Glyma05g30020 Glyma14g23570 Glyma03g04500 Glyma12g06430 Glyma08g06010 Glyma18g02590 Glyma01g03650 Glyma10g04390 Glyma05g32030 Glyma14g35410 Glyma03g06350 Glyma12g06450 Glyma08g09270 Glyma18g02820 Glyma01g04570 Glyma10g04960 Glyma05g34630 Glyma15g02560 Glyma03g21710 Glyma12g10150 Glyma08g09640 Glyma18g03360 Glyma01g20670 Glyma10g05520 Glyma05g37470 Glyma15g02640 Glyma03g23740 Glyma12g15470 Glyma08g09740 Glyma18g10060 Glyma01g21770 Glyma10g05870 Glyma06g01790 Glyma15g02730 Glyma03g23760 Glyma12g17950 Glyma08g11940 Glyma18g10170 Glyma01g23500 Glyma10g06590 Glyma06g02880 Glyma15g05570 Glyma03g27030 Glyma12g27340 Glyma08g13130 Glyma18g10340 Glyma01g31360 Glyma10g15850 Glyma06g03230 Glyma15g06560 Glyma03g27290 Glyma12g28730 Glyma08g15340 Glyma18g12340 Glyma01g36410 Glyma10g29000 Glyma06g03560 Glyma15g08920 Glyma03g34840 Glyma12g30590 Glyma08g17460 Glyma18g46130 Glyma01g36990 Glyma10g29250 Glyma06g04980 Glyma15g11380 Glyma03g38830 Glyma12g34360 Glyma08g18110 Glyma18g48280 Glyma01g38320 Glyma10g30140 Glyma06g07420 Glyma15g13220 Glyma03g39210 Glyma12g35150 Glyma08g18240 Glyma18g52600 Glyma01g38410 Glyma10g30970 Glyma06g08260 Glyma15g18450 Glyma03g41810 Glyma12g35340 Glyma08g19420 Glyma18g53870 Glyma01g41660 Glyma10g36770 Glyma06g08380 Glyma15g20950 Glyma03g42450 Glyma12g36350 Glyma08g20830 Glyma18g53880 Glyma01g43110 Glyma10g38400 Glyma06g11850 Glyma15g40750 Glyma03g42540 Glyma12g36910 Glyma08g21030 Glyma19g07240 Glyma01g43150 Glyma10g39330 Glyma06g13410 Glyma15g40860 Glyma0430s0020Glyma1337s00200Glyma08g21040 Glyma19g27920 Glyma01g43680 Glyma10g39780 Glyma06g14030 Glyma15g41680 Glyma04g01690 Glyma13g03210 Glyma08g21050 Glyma19g29420 Glyma01g45380 Glyma10g40340 Glyma06g16940 Glyma15g42900 Glyma04g02860 Glyma13g10330 Glyma08g21080 Glyma19g30250 Glyma02g00540 Glyma10g42180 Glyma06g17930 Glyma16g00400 Glyma04g03180 Glyma13g17200 Glyma08g23370 Glyma19g35550 Glyma02g01160 Glyma10g42250 Glyma06g18110 Glyma16g01230 Glyma04g03490 Glyma13g18640 Glyma08g23900 Glyma19g37530 Glyma02g01750 Glyma10g42490 Glyma06g18120 Glyma16g03170 Glyma04g04880 Glyma13g19310 Glyma08g24210 Glyma19g40810 Glyma02g02990 Glyma10g42940 Glyma06g20310 Glyma16g04060 Glyma04g07370 Glyma13g19870 Glyma08g32090 Glyma19g41400 Glyma02g04740 Glyma11g00370 Glyma06g22050 Glyma16g04080 Glyma04g08200 Glyma13g20220 Glyma08g40910 Glyma19g44460 Glyma02g04980 Glyma11g01800 Glyma06g32680 Glyma16g06260 Glyma04g08280 Glyma13g20790 Glyma08g42410 Glyma19g45320 Glyma02g06710 Glyma11g02340 Glyma06g33840 Glyma16g06270 Glyma04g12950 Glyma13g27570 Glyma08g43250 Glyma20g03020 Glyma02g08870 Glyma11g02410 Glyma06g36150 Glyma16g08200 Glyma04g18930 Glyma13g32750 Glyma08g43390 Glyma20g05050 Glyma02g09690 Glyma11g03700 Glyma06g38270 Glyma16g08590 Glyma04g32460 Glyma13g33910 Glyma08g43480 Glyma20g10030 Glyma02g17990 Glyma11g06900 Glyma06g42840 Glyma16g10700 Glyma04g33520 Glyma13g35170 Glyma08g45420 Glyma20g10810 Glyma02g22290 Glyma11g06910 Glyma06g47830 Glyma16g13410 Glyma04g34170 Glyma13g35400

Glyma08g45430 Glyma20g10820 Glyma02g22300 Glyma11g08290 Glyma07g00280 Glyma16g22530 Glyma04g36860 Glyma13g35580 Glyma08g47600 Glyma20g24070 Glyma02g26650 Glyma11g08990 Glyma07g00520 Glyma16g23010 Glyma04g36870 Glyma13g35590 Glyma08g47610 Glyma20g24550 Glyma02g32980 Glyma11g10120 Glyma07g01440 Glyma16g25450 Glyma04g37120 Glyma13g42800 Glyma09g00250 Glyma20g24790 Glyma02g37120 Glyma11g12230 Glyma07g01570 Glyma16g25740 Glyma04g38120 Glyma13g42880 Glyma09g02310 Glyma20g24850 Glyma02g38540 Glyma11g14050 Glyma07g02640 Glyma16g26330 Glyma04g40760 Glyma14g02130 Glyma09g04950 Glyma20g27000 Glyma02g41770 Glyma11g14500 Glyma07g04640 Glyma16g27970 Glyma04g41440 Glyma14g03970 Glyma09g07120 Glyma20g28440 Glyma02g42820 Glyma11g14530 Glyma07g06580 Glyma17g01800 Glyma04g42900 Glyma14g04550 Glyma09g08870 Glyma20g29460 Glyma02g43050 Glyma11g14550 Glyma07g06590 Glyma17g04330 Glyma05g03510 Glyma14g05920 Glyma09g15900 Glyma20g30820 Glyma02g43060 Glyma11g17930 Glyma07g08370 Glyma17g04340 Glyma05g07580 Glyma14g07190 Glyma09g23690 Glyma20g32400 Glyma02g43070 Glyma11g34980 Glyma07g13690 Glyma17g05530 Glyma05g14280 Glyma14g07960 Glyma09g29030 Glyma20g36510 Glyma02g43080 Glyma11g35600 Glyma07g27460 Glyma17g09060 Glyma05g21330 Glyma14g09880 Glyma09g34310 Glyma20g37280 Glyma02g44220 Glyma11g35820 Glyma07g35160 Glyma17g11440 Glyma05g24110 Glyma14g10920 Glyma09g38110 Glyma20g38030 Glyma02g44760 Glyma11g37960 Glyma07g35790 Glyma17g14070 Glyma05g26360 Glyma14g10950 Glyma09g40070 Glyma20g38320 Glyma02g44770 Glyma11g37970 Glyma07g36150 Glyma17g24650 Glyma05g26630 Glyma14g10960 Glyma10g00890 Glyma20g38990 Glyma02g46510 Glyma12g02440 Glyma07g38940 Glyma17g34610 Glyma05g26750 Glyma14g16200 Glyma03g01880 Glyma12g04430 Glyma08g02070 Glyma17g37050

## Module 59

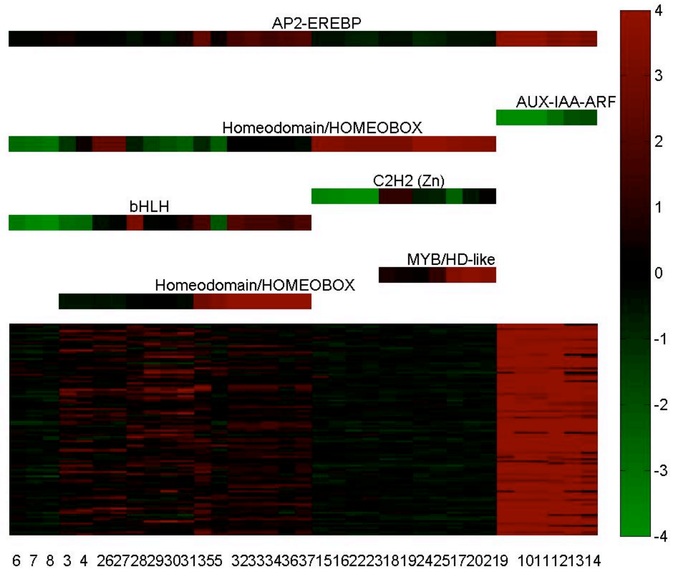

| GO_ACC | GO name | P value |
|---|---|---|
| GO:0006457 | P:protein folding | 0.05 |
| GO:0055114 | P:oxidation reduction | 0.01 |
| GO:0006869 | P:lipid transport | 0.0017356 |

| TF | Family | Binding site | Gene number |
|---|---|---|---|
| Glyma03 g25280 | bHLH | | |
| Glyma11 g02450 | Homeodomain /HOMEOBOX | | |
| Glyma13 g00950 | AP2-EREBP | | |
| Glyma13 g34530 | C2H2 (Zn) | 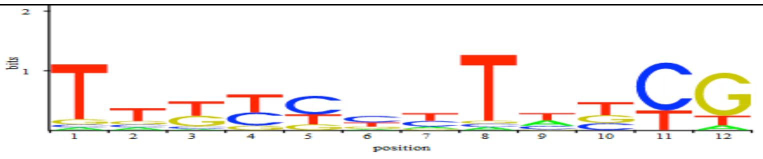 | 45 |
| Glyma14 g10430 | Homeodomain /HOMEOBOX | | |
| Glyma15 g02040 | AUX-IAA-ARF | | |
| Glyma18 g05050 | MYB/HD-like | | |

Glyma01g34650 Glyma11g21310 Glyma07g08130 Glyma15g13750 Glyma04g05440 Glyma13g25630 Glyma08g42290 Glyma18g04490 Glyma01g35650 Glyma11g29060 Glyma07g08950 Glyma15g13760 Glyma04g08990 Glyma13g25710 Glyma08g42490 Glyma18g06610 Glyma01g37760 Glyma11g29070 Glyma07g28520 Glyma15g13770 Glyma04g10900 Glyma13g30890 Glyma08g43620 Glyma18g06660 Glyma01g41310 Glyma11g29310 Glyma07g37600 Glyma15g14330 Glyma04g33390 Glyma13g31410 Glyma08g43650 Glyma18g11820 Glyma01g43000 Glyma11g36490 Glyma07g39960 Glyma15g18720 Glyma05g24420 Glyma13g35610 Glyma09g03400 Glyma18g49130 Glyma02g02550 Glyma12g12300 Glyma07g39970 Glyma15g19460 Glyma05g27650 Glyma13g35890 Glyma09g07990 Glyma19g00700 Glyma02g17330 Glyma12g12800 Glyma08g02260 Glyma15g23790 Glyma05g27810 Glyma14g04790 Glyma09g32780 Glyma19g29330 Glyma02g43470 Glyma12g26170 Glyma08g07670 Glyma16g03470 Glyma05g30760 Glyma14g04800 Glyma10g02460 Glyma19g29670 Glyma02g44100 Glyma12g34610 Glyma08g10640 Glyma16g33120 Glyma05g37290 Glyma14g04810 Glyma10g05450 Glyma19g30820

Glyma03g01700 Glyma12g34740 Glyma08g10800 Glyma16g33140 Glyma06g02900 Glyma14g07880 Glyma10g07140 Glyma19g33840 Glyma03g02260 Glyma12g34960 Glyma08g13950 Glyma17g03050 Glyma06g03100 Glyma14g09710 Glyma10g31540 Glyma19g37910 Glyma03g02500 Glyma13g00440 Glyma08g17100 Glyma17g06560 Glyma06g04530 Glyma14g10090 Glyma10g31640 Glyma19g38400 Glyma03g02980 Glyma13g06550 Glyma08g19580 Glyma17g13380 Glyma06g08800 Glyma14g10100 Glyma10g33350 Glyma20g26250 Glyma03g24830 Glyma13g19800 Glyma08g21430 Glyma17g29170 Glyma06g09080 Glyma14g16710 Glyma10g36930 Glyma20g26730 Glyma03g28060 Glyma13g25520 Glyma08g22380 Glyma17g29320 Glyma06g10750 Glyma14g23710 Glyma10g37150 Glyma20g33090 Glyma03g34760 Glyma13g25540 Glyma08g24340 Glyma17g35090 Glyma06g11860 Glyma15g05470 Glyma10g41030 Glyma20g34300 Glyma04g02880 Glyma13g25590 Glyma08g25650 Glyma17g35100 Glyma06g35630 Glyma15g07930 Glyma11g02490 Glyma20g35960 Glyma04g04390 Glyma13g25600 Glyma08g34500 Glyma17g35460 Glyma07g01760 Glyma15g08420 Glyma11g07550 Glyma20g36040 Glyma07g06960 Glyma15g13740 Glyma11g15490

# Module 63

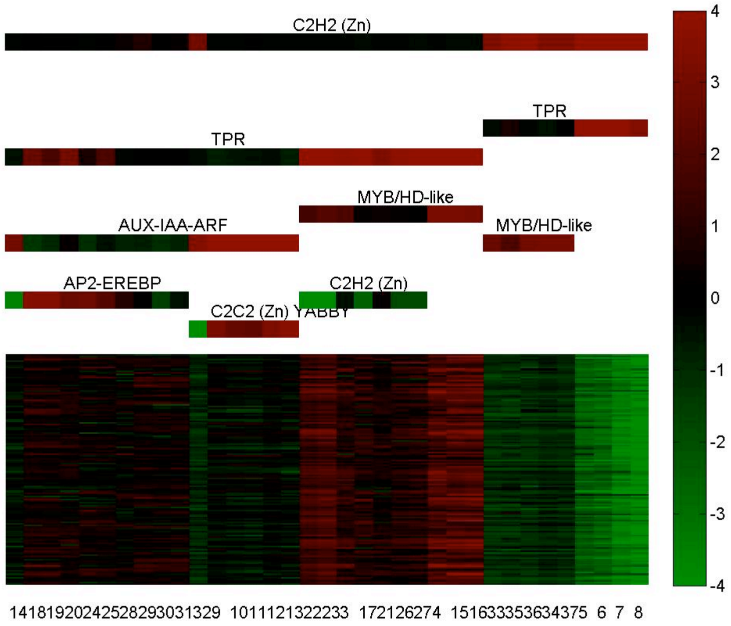


| GO_ACC | GO name | P value |
|---|---|---|
| GO:0006412 | P:translation | 9.5E-10 |
| GO:0045454 | P:cell redox homeostasis | 0.0025249 |
| GO:0016117 | P:carotenoid biosynthetic process | 0.00000206 |
| GO:0006633 | P:fatty acid biosynthetic process | 0.04 |
| GO:0006353 | P:transcription termination | 0.00000928 |
| GO:0009416 | P:response to light stimulus | 0.0034089 |

| TF | Family | Binding site | Gene number |
|---|---|---|---|
| Glyma01 g04310 | C2H2 (Zn) | 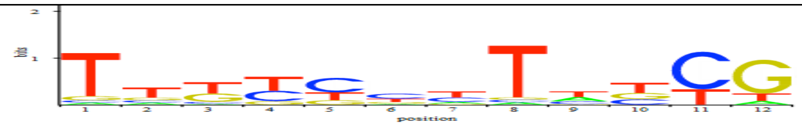 | 41 |
| Glyma03 g27750 | TPR | | |
| Glyma06 g17980 | MYB/HD-like | | |
| Glyma07 g37410 | AP2-EREBP | 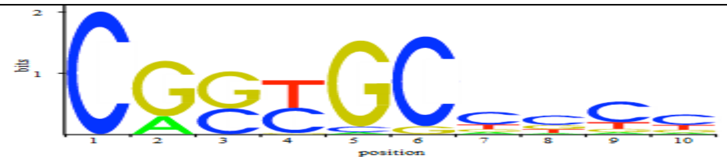 | 8 |
| Glyma13 g22620 | C2C2 (Zn) YABBY | 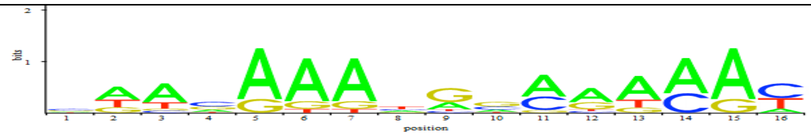 | 42 |
| Glyma13 g34530 | C2H2 (Zn) | 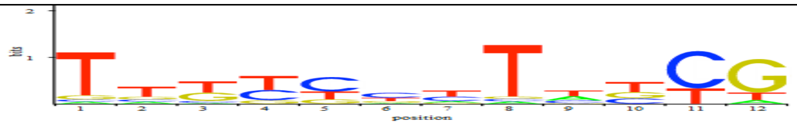 | 41 |
| Glyma13 g43050 | AUX-IAA-ARF | | |
| Glyma15 g03920 | MYB/HD-like | | |
| Glyma12 g31490 | TPR | | |

Glyma01g01910 Glyma09g02060 Glyma05g15170 Glyma13g42500 Glyma03g28470 Glyma11g07790 Glyma07g00410 Glyma18g02950 Glyma01g07560 Glyma09g03440 Glyma05g22370 Glyma13g45030 Glyma03g34050 Glyma11g10310 Glyma07g02210 Glyma18g05600 Glyma01g09310 Glyma09g03770 Glyma05g22380 Glyma14g01130 Glyma03g35470 Glyma11g10550 Glyma07g02580 Glyma18g20220 Glyma01g16380 Glyma09g09110 Glyma05g29040 Glyma14g01290 Glyma03g37450 Glyma11g12070 Glyma07g06240 Glyma18g44620 Glyma01g26990 Glyma09g09190 Glyma05g32720 Glyma14g02110

Glyma03g38550 Glyma11g14460 Glyma07g06420 Glyma18g53310 Glyma01g32790 Glyma09g15720 Glyma05g32900 Glyma14g10110 Glyma03g38960 Glyma11g20920 Glyma07g30110 Glyma18g53760 Glyma01g37500 Glyma09g15730 Glyma05g34500 Glyma14g26450 Glyma03g41230 Glyma11g21160 Glyma07g31830 Glyma19g03440 Glyma01g39960 Glyma09g35270 Glyma05g35240 Glyma14g26460 Glyma04g00330 Glyma11g25540 Glyma07g35080 Glyma19g31030 Glyma01g40770 Glyma09g41150 Glyma05g35480 Glyma14g31370 Glyma04g02000 Glyma11g26510 Glyma07g36470 Glyma19g31220 Glyma01g41840 Glyma10g01510 Glyma06g00390 Glyma15g12560 Glyma04g02680 Glyma11g30140 Glyma08g00380 Glyma19g38110 Glyma01g43270 Glyma10g01550 Glyma06g01760 Glyma15g14360 Glyma04g02690 Glyma11g35470 Glyma08g00520 Glyma19g40050 Glyma02g00780 Glyma10g04630 Glyma06g02100 Glyma15g14710 Glyma04g04130 Glyma11g35500 Glyma08g04250 Glyma19g41150 Glyma02g01350 Glyma10g06500 Glyma06g02710 Glyma15g20680 Glyma04g04160 Glyma11g35910 Glyma08g04480 Glyma19g41510 Glyma02g01510 Glyma10g06620 Glyma06g04300 Glyma15g41980 Glyma04g08750 Glyma11g36810 Glyma08g07200 Glyma19g43510 Glyma02g08050 Glyma10g29900 Glyma06g04340 Glyma16g02880 Glyma04g09640 Glyma12g03760 Glyma08g07880 Glyma19g43850 Glyma02g08550 Glyma10g30720 Glyma06g08850 Glyma16g05050 Glyma04g17310 Glyma12g06380 Glyma08g12190 Glyma20g02940 Glyma02g26820 Glyma10g30920 Glyma06g09740 Glyma16g09190 Glyma04g24360 Glyma12g11030 Glyma08g17220 Glyma20g23250 Glyma02g40720 Glyma10g35840 Glyma06g12930 Glyma16g25560 Glyma04g24960 Glyma12g11620 Glyma08g18360 Glyma20g29640 Glyma02g42300 Glyma10g36070 Glyma06g14530 Glyma17g04130 Glyma04g26220 Glyma12g18970 Glyma08g21880 Glyma20g31530 Glyma02g46530 Glyma10g38200 Glyma06g14920 Glyma17g10240 Glyma04g37020 Glyma12g35880 Glyma08g23440 Glyma20g31750 Glyma02g47460 Glyma10g38950 Glyma06g14930 Glyma17g10420 Glyma04g40250 Glyma12g36260 Glyma08g24010 Glyma20g31940 Glyma02g47960 Glyma10g40970 Glyma06g17980 Glyma17g17380 Glyma04g41870 Glyma13g05920 Glyma08g25420 Glyma20g34740 Glyma03g01980 Glyma10g43550 Glyma06g18560 Glyma17g17470 Glyma05g00260 Glyma13g18930 Glyma08g35830 Glyma20g36540 Glyma03g04350 Glyma10g44560 Glyma06g19210 Glyma18g00720 Glyma05g01460 Glyma13g20700 Glyma08g47730 Glyma20g37020 Glyma03g14960 Glyma11g04560 Glyma06g30020 Glyma18g02490 Glyma05g01480 Glyma13g29310 Glyma08g48210 Glyma20g37440 Glyma03g24210 Glyma11g05330 Glyma06g45440 Glyma18g02800 Glyma05g01650 Glyma13g31570 Glyma09g01630 Glyma20g39340 Glyma03g28290 Glyma11g06470 Glyma06g45770 Glyma18g02910 Glyma05g06470 Glyma13g34500

## Module 66

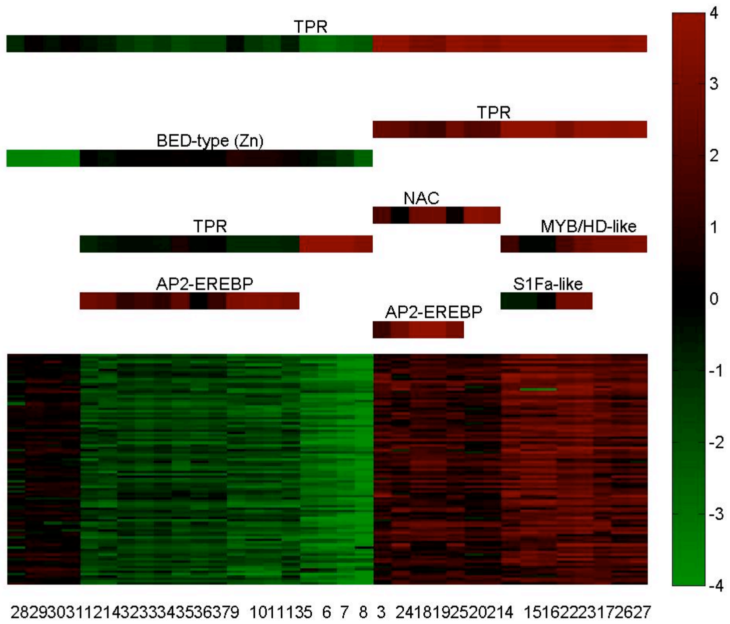


| GO_ACC | GO name | P value |
|---|---|---|
| GO:0016055 | P:Wnt receptor signaling pathway | 0.00029203 |
| GO:0055114 | P:oxidation reduction | 0.0099734 |
| GO:0006915 | P:apoptosis | 0.02 |
| GO:0030178 | P:negative regulation of Wnt receptor signali... | 0.0000136 |
| GO:0006917 | P:induction of apoptosis | 0.00010869 |
| GO:0006508 | P:proteolysis | 0.0062271 |
| GO:0048705 | P:skeletal system morphogenesis | 0.00000685 |
| GO:0006457 | P:protein folding | 0.0099339 |
| GO:0006260 | P:DNA replication | 0.00056814 |
| GO:0001649 | P:osteoblast differentiation | 0.00000685 |
| GO:0006810 | P:transport | 0.02 |

| TF | Family | Binding site | Gene number |
|---|---|---|---|
| Glyma02g08840 | AP2-EREBP | 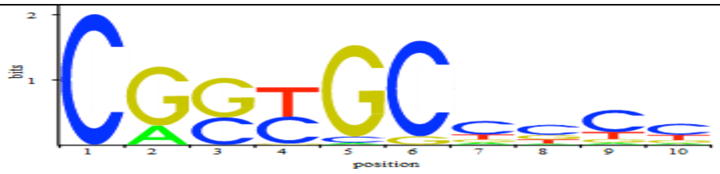 | 6 |
| Glyma04g24430 | TPR | | |

| | | | |
|---|---|---|---|
| Glyma06 g30000 | TPR | | |
| Glyma09 g15130 | BED-type (Zn) | 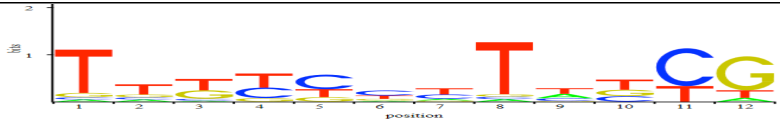 | 48 |
| Glyma09 g33240 | AP2-EREBP | 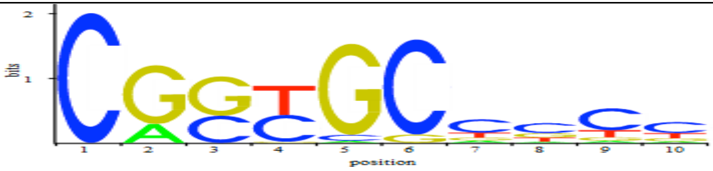 | 6 |
| Glyma10 g05790 | S1Fa-like | | |
| Glyma13 g35550 | NAC | | |
| Glyma16 g01980 | MYB/HD-like | 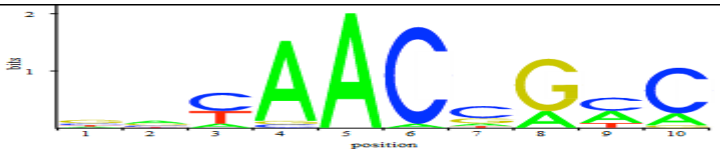 | 5 |
| Glyma12 g31490 | TPR | | |

Glyma01g00640 Glyma11g03990 Glyma06g15070 Glyma14g39620 Glyma03g23870 Glyma12g30260 Glyma09g05600 Glyma18g00310 Glyma01g15420 Glyma11g04530 Glyma06g15200 Glyma15g16870 Glyma03g24780 Glyma12g30830 Glyma09g26700 Glyma18g03970 Glyma01g35170 Glyma11g06060 Glyma06g16570 Glyma15g24680 Glyma03g28240 Glyma13g00500 Glyma09g29840 Glyma18g32900 Glyma01g38940 Glyma11g10710 Glyma06g19710 Glyma16g02080 Glyma03g30220 Glyma13g00520 Glyma09g34590 Glyma18g47200 Glyma01g39200 Glyma11g11750 Glyma06g23560 Glyma16g03030 Glyma04g03820 Glyma13g04720 Glyma09g36480 Glyma19g00910 Glyma01g41430 Glyma11g13320 Glyma06g35690 Glyma16g06130 Glyma04g07170 Glyma13g04730 Glyma10g05010 Glyma19g01260 Glyma02g06170 Glyma11g34350 Glyma06g46300 Glyma16g25200 Glyma04g07870 Glyma13g16610 Glyma10g05560 Glyma19g25820 Glyma02g09640 Glyma11g36830 Glyma07g00240 Glyma16g34360 Glyma04g10170 Glyma13g18000 Glyma10g06210 Glyma19g29940 Glyma02g10920 Glyma11g36840 Glyma07g02350 Glyma17g04510 Glyma04g10550 Glyma13g19370 Glyma10g08680 Glyma19g32770 Glyma02g15120 Glyma12g01680 Glyma07g05540 Glyma17g06660 Glyma04g10650 Glyma13g20520 Glyma10g24540 Glyma19g33130 Glyma02g15130 Glyma12g01690 Glyma07g15440 Glyma17g12380 Glyma04g35040 Glyma13g20700 Glyma10g27980 Glyma19g36130 Glyma02g17130 Glyma12g02850 Glyma08g07260 Glyma17g14950 Glyma04g39670 Glyma13g23640 Glyma10g27990 Glyma20g25940

Glyma02g36240 Glyma12g03020 Glyma08g16100 Glyma17g18010 Glyma05g03340 Glyma13g23850 Glyma10g35180 Glyma20g30080 Glyma02g41250 Glyma12g04100 Glyma08g23670 Glyma17g29740 Glyma05g21780 Glyma13g39470 Glyma10g37750 Glyma20g31140 Glyma02g47350 Glyma12g04110 Glyma08g24280 Glyma17g33600 Glyma05g29820 Glyma14g01410 Glyma10g37760 Glyma20g37170 Glyma03g00780 Glyma12g05280 Glyma08g26000 Glyma17g34480 Glyma05g32600 Glyma14g10820 Glyma10g41310 Glyma20g37770 Glyma03g00790 Glyma12g26520 Glyma08g46150 Glyma17g34690 Glyma06g07270 Glyma14g17300 Glyma10g44020 Glyma20g38820 Glyma06g10160 Glyma14g34170 Glyma10g44120

# Module 67

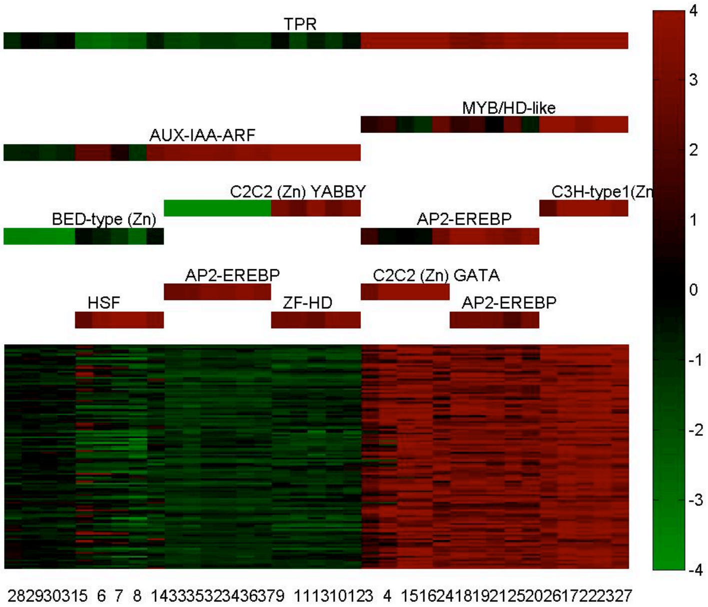


| aaaaGO_ACC | GO name | P value |
|---|---|---|
| GO:0006350 | P:transcription | 0.0023192 |
| GO:0009734 | P:auxin mediated signaling pathway | 0.0081016 |
| GO:0015979 | P:photosynthesis | 0.0084998 |
| GO:0045449 | P:regulation of transcription | 0.01 |

| TF | Family | Binding site | Gene number |
|---|---|---|---|
| Glyma02 g08840 | AP2- EREBP | 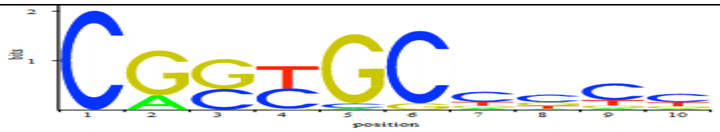 | 10 |

| | | | |
|---|---|---|---|
| Glyma04g09910 | ZF-HD | | |
| Glyma04g24430 | TPR | | |
| Glyma08g43790 | C3H-type1(Zn) | | |
| Glyma09g15130 | BED-type (Zn) | 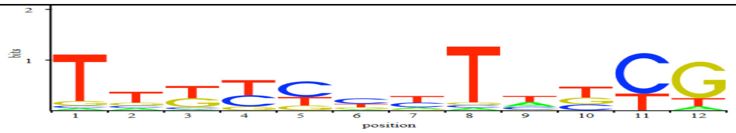 | 70 |
| Glyma10g34760 | AP2-EREBP | 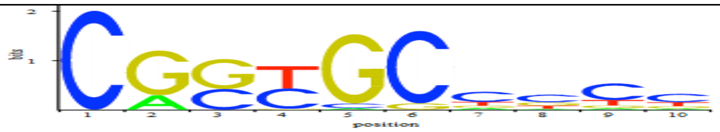 | 10 |
| Glyma13g22620 | C2C2 (Zn) YABBY | 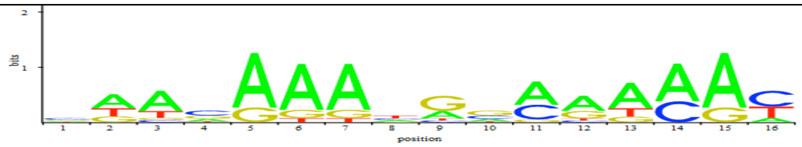 | 70 |
| Glyma13g43050 | AUX-IAA-ARF | | |
| Glyma14g10830 | C2C2 (Zn) GATA | | |
| Glyma19g36200 | AP2-EREBP | 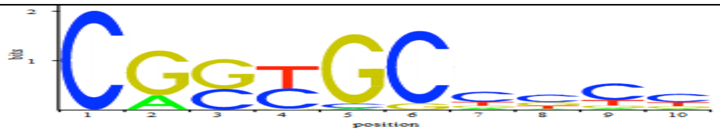 | 10 |
| Glyma19g45030 | MYB/HD-like | 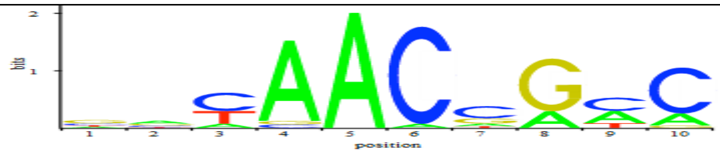 | 70 |

Glyma01g15930 Glyma11g34350 Glyma07g09840 Glyma15g06730 Glyma05g03340 Glyma13g15560 Glyma09g27230 Glyma17g10010 Glyma01g35910 Glyma12g00300 Glyma07g10840 Glyma15g06800 Glyma05g03580 Glyma13g19390 Glyma09g31970 Glyma17g10990 Glyma01g39970 Glyma12g04890 Glyma07g15500 Glyma15g07110 Glyma05g07680 Glyma13g23380 Glyma09g39840 Glyma17g11450

Glyma02g00380 Glyma12g08010 Glyma07g38390 Glyma15g08770 Glyma05g09180 Glyma13g29490 Glyma09g39850 Glyma17g13690 Glyma02g01840 Glyma12g08400 Glyma07g39180 Glyma15g09560

Glyma05g28530 Glyma13g30450 Glyma10g00440 Glyma17g13930 Glyma02g05760 Glyma12g15560 Glyma07g39370 Glyma15g09570 Glyma05g33920 Glyma13g32200 Glyma10g03200 Glyma17g14130 Glyma02g16620 Glyma12g17280 Glyma07g40140 Glyma15g10870 Glyma05g38290 Glyma13g32600 Glyma10g05030 Glyma17g14140 Glyma02g43360 Glyma12g29810 Glyma08g01360 Glyma15g43150 Glyma06g02040 Glyma13g33950 Glyma10g06830 Glyma17g31330 Glyma02g47990 Glyma12g31770 Glyma08g05730 Glyma16g02050 Glyma06g03670 Glyma13g34750 Glyma10g11580 Glyma18g03970 Glyma03g04950 Glyma12g33430 Glyma08g11560 Glyma16g04600 Glyma06g03920 Glyma13g35560 Glyma10g13720 Glyma18g06920 Glyma03g06640 Glyma12g34230 Glyma08g18170 Glyma16g15790 Glyma06g04280 Glyma13g35620 Glyma10g27910 Glyma18g17190 Glyma03g17360 Glyma12g34950 Glyma08g18200 Glyma16g24420 Glyma06g06530 Glyma13g36320 Glyma11g01690 Glyma18g19730 Glyma03g38260 Glyma12g35670 Glyma08g26340 Glyma16g29940 Glyma06g12470 Glyma13g39980 Glyma11g05320 Glyma18g20870 Glyma04g01920 Glyma13g01560 Glyma08g27150 Glyma16g32400 Glyma06g15220 Glyma13g44740 Glyma11g06510 Glyma18g46350 Glyma04g02210 Glyma13g09700 Glyma08g35480 Glyma17g00650 Glyma06g17760 Glyma14g01950 Glyma11g10440 Glyma18g51490 Glyma04g03580 Glyma13g09730 Glyma08g38840 Glyma17g01560 Glyma06g42800 Glyma14g07680 Glyma11g12860 Glyma18g53710 Glyma04g04110 Glyma13g09740 Glyma08g45280 Glyma17g02370 Glyma06g43240 Glyma14g11960 Glyma11g12870 Glyma19g01220 Glyma04g06480 Glyma13g09760 Glyma08g45790 Glyma17g02610 Glyma06g43290 Glyma14g15120 Glyma11g15920 Glyma19g28700 Glyma04g37320 Glyma13g09780 Glyma09g02210 Glyma17g06660 Glyma06g43380 Glyma15g00560 Glyma11g16260 Glyma20g05700 Glyma04g39650 Glyma13g09790 Glyma09g03190 Glyma17g07670 Glyma06g43520 Glyma15g02200 Glyma11g20070 Glyma20g30480 Glyma05g02050 Glyma13g09820 Glyma09g03200 Glyma17g09170 Glyma07g08030 Glyma15g05980 Glyma11g27280 Glyma05g03030 Glyma13g09870 Glyma09g03230 Glyma17g09870

## Module 69

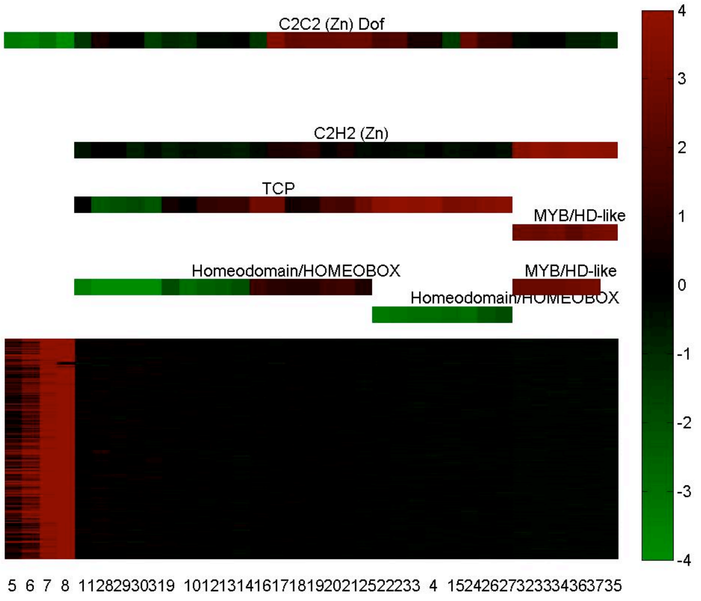

| GO_ACC | GO name | P value |
|---|---|---|
| GO:0051480 | P:cytosolic calcium ion homeostasis | 0.000000122 |
| GO:0051592 | P:response to calcium ion | 0.00000409 |
| GO:0051281 | P:positive regulation of release of sequester... | 0.000000122 |
| GO:0006816 | P:calcium ion transport | 0.00037923 |
| GO:0055114 | P:oxidation reduction | 0.99 |

| TF | Family |
|---|---|
| Glyma03g00980 | MYB/HD-like |
| Glyma14g10430 | Homeodomain/HOMEOBOX |
| Glyma14g13360 | C2H2 (Zn) |
| Glyma17g10920 | C2C2 (Zn) Dof |
| Glyma17g11330 | Homeodomain/HOMEOBOX |
| Glyma19g03810 | TCP |
| Glyma20g29710 | MYB/HD-like |

Glyma09g38550 Glyma18g03680 Glyma18g03640 Glyma18g03710

Glyma17g01760 Glyma18g03690 Glyma18g03660 Glyma18g03720

Glyma18g03630 Glyma18g03700 Glyma18g03670 Glyma18g47770

# Module 71

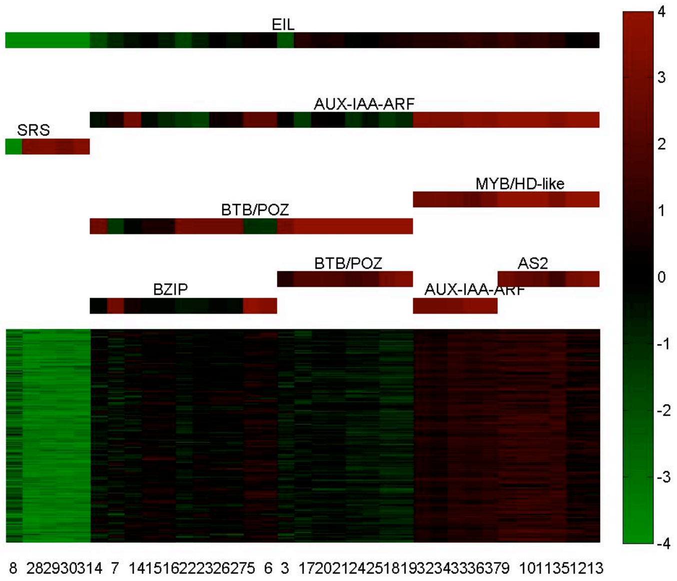


| GO_ACC | GO name | P value |
|---|---|---|
| GO:0006886 | P:intracellular protein transport | 0.000000802 |
| GO:0007264 | P:small GTPase mediated signal transduction | 0.0000548 |
| GO:0055085 | P:transmembrane transport | 0.01 |
| GO:0051301 | P:cell division | 0.007621 |
| GO:0042254 | P:ribosome biogenesis | 0.0020853 |
| GO:0042026 | P:protein refolding | 0.00051609 |
| GO:0030154 | P:cell differentiation | 0.02 |
| GO:0018401 | P:peptidyl-proline hydroxylation to 4-hydroxy... | 0.00012816 |
| GO:0016192 | P:vesicle-mediated transport | 0.000000157 |
| GO:0015780 | P:nucleotide-sugar transport | 0.00072701 |
| GO:0015031 | P:protein transport | 1.43E-10 |
| GO:0008283 | P:cell proliferation | 0.00010751 |
| GO:0006096 | P:glycolysis | 0.02 |
| GO:0000902 | P:cell morphogenesis | 0.00051609 |
| GO:0007047 | P:cellular cell wall organization | 0.03 |
| GO:0001525 | P:angiogenesis | 0.000065 |
| GO:0007067 | P:mitosis | 0.0000711 |
| GO:0006350 | P:transcription | 0.99 |
| GO:0006412 | P:translation | 0.000141 |
| GO:0006457 | P:protein folding | 0.03 |
| GO:0006468 | P:protein amino acid phosphorylation | 0.01 |
| GO:0006511 | P:ubiquitin-dependent protein catabolic process | 0.00000595 |
| GO:0006914 | P:autophagy | 0.00024447 |
| GO:0006414 | P:translational elongation | 0.000059 |

| TF | Family | Binding site | Gene number |
|---|---|---|---|
| Glyma02 g38260 | AUX-IAA-ARF | | |
| Glyma02 g40360 | BTB/POZ | | |
| Glyma02 g44220 | EIL | | |
| Glyma02 g44860 | SRS | | |
| Glyma03 g00980 | MYB/HD-like | | |
| Glyma13 g43050 | AUX-IAA-ARF | | |
| Glyma14 g39820 | BTB/POZ | | |
| Glyma20 g31780 | AS2 | | |
| Glyma12 g30990 | BZIP | 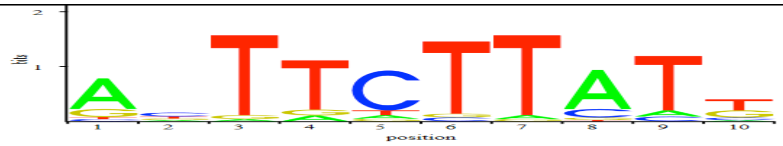 | 58 |

Glyma0060s00220 Glyma10g31600 Glyma06g07000 Glyma14g39740 Glyma03g40710
Glyma13g05140 Glyma08g15380 Glyma18g20720 Glyma01g01460 Glyma10g32500 Glyma06g07290
Glyma15g00900 Glyma03g41250 Glyma13g05310 Glyma08g15680 Glyma18g27720 Glyma01g01960
Glyma10g35450 Glyma06g08200 Glyma15g01520 Glyma03g41950 Glyma13g05760 Glyma08g17000
Glyma18g32830 Glyma01g02640 Glyma10g35830 Glyma06g08670 Glyma15g05490 Glyma04g00200
Glyma13g07800 Glyma08g19520 Glyma18g44980 Glyma01g03330 Glyma10g40340 Glyma06g08810
Glyma15g06530 Glyma04g01100 Glyma13g10530 Glyma08g20100 Glyma18g47190 Glyma01g03930
Glyma10g43590 Glyma06g13280 Glyma15g07760 Glyma04g01460 Glyma13g11390 Glyma08g20300
Glyma18g47590 Glyma01g04320 Glyma10g43840 Glyma06g13850 Glyma15g11630 Glyma04g01620

Glyma13g16070 Glyma08g24120 Glyma18g47910 Glyma01g21680 Glyma10g44250 Glyma06g14080
Glyma15g14420 Glyma04g03810 Glyma13g16100 Glyma08g26900 Glyma18g50150 Glyma01g24430
Glyma10g44510 Glyma06g16130 Glyma15g18600 Glyma04g05340 Glyma13g18640 Glyma08g28320
Glyma18g51310 Glyma01g33240 Glyma11g03580 Glyma06g16940 Glyma15g27510 Glyma04g05620
Glyma13g18690 Glyma08g29030 Glyma18g51900 Glyma01g36870 Glyma11g03590 Glyma06g28570
Glyma15g37580 Glyma04g06580 Glyma13g18730 Glyma08g35490 Glyma18g52430 Glyma01g41790
Glyma11g08390 Glyma06g30880 Glyma15g40840 Glyma04g06920 Glyma13g18950 Glyma08g39080
Glyma18g52860 Glyma01g41800 Glyma11g09990 Glyma06g34190 Glyma15g40850 Glyma04g07190
Glyma13g19640 Glyma08g42240 Glyma19g00500 Glyma01g41810 Glyma11g10800 Glyma06g39430
Glyma15g40860 Glyma04g08130 Glyma13g21870 Glyma08g44410 Glyma19g02110 Glyma02g03370
Glyma11g10860 Glyma06g39710 Glyma15g42150 Glyma04g08560 Glyma13g22720 Glyma08g44960
Glyma19g02540 Glyma02g03750 Glyma11g11510 Glyma06g39770 Glyma16g00540 Glyma04g08710
Glyma13g23670 Glyma08g45000 Glyma19g03950 Glyma02g04280 Glyma11g11610 Glyma06g42990
Glyma16g02670 Glyma04g12370 Glyma13g24850 Glyma08g46090 Glyma19g03960 Glyma02g05370
Glyma11g11950 Glyma06g44340 Glyma16g04590 Glyma04g16190 Glyma13g24950 Glyma09g00460
Glyma19g06930 Glyma02g05540 Glyma11g12200 Glyma06g46490 Glyma16g23730 Glyma04g23560
Glyma13g25100 Glyma09g00770 Glyma19g07790 Glyma02g08920 Glyma11g12600 Glyma06g47400
Glyma16g24120 Glyma04g32380 Glyma13g25110 Glyma09g03480 Glyma19g28710 Glyma02g09390
Glyma11g19220 Glyma06g48070 Glyma16g24270 Glyma04g38120 Glyma13g31550 Glyma09g07410
Glyma19g34900 Glyma02g10470 Glyma11g19660 Glyma07g00250 Glyma16g25440 Glyma04g38770
Glyma13g32790 Glyma09g23980 Glyma19g34930 Glyma02g10880 Glyma11g30430 Glyma07g00350
Glyma16g29450 Glyma04g40720 Glyma13g33960 Glyma09g26720 Glyma19g35140 Glyma02g37700
Glyma11g33380 Glyma07g00950 Glyma16g33380 Glyma04g40990 Glyma13g36640 Glyma09g28650
Glyma19g35280 Glyma02g38460 Glyma11g33480 Glyma07g06070 Glyma16g34510 Glyma04g41000
Glyma13g37610 Glyma09g29970 Glyma19g35460 Glyma02g41470 Glyma11g36150 Glyma07g07920
Glyma17g02430 Glyma04g41540 Glyma13g40920 Glyma09g31100 Glyma19g37330 Glyma02g42260
Glyma11g37760 Glyma07g07950 Glyma17g03140 Glyma05g05860 Glyma13g43830 Glyma09g32430
Glyma19g37520 Glyma02g43080 Glyma12g02330 Glyma07g09270 Glyma17g08880 Glyma05g06390
Glyma13g44380 Glyma09g32510 Glyma19g39800 Glyma02g43930 Glyma12g03080 Glyma07g09370
Glyma17g11800 Glyma05g23230 Glyma14g00360 Glyma09g33290 Glyma19g40690 Glyma02g44090
Glyma12g03140 Glyma07g23230 Glyma17g12110 Glyma05g26250 Glyma14g00460 Glyma09g33950
Glyma19g40930 Glyma02g47210 Glyma12g03660 Glyma07g26540 Glyma17g12410 Glyma05g28680
Glyma14g01530 Glyma09g34310 Glyma19g41400 Glyma02g48070 Glyma12g04210 Glyma07g30090
Glyma17g16200 Glyma05g30210 Glyma14g04780 Glyma09g38430 Glyma19g41420 Glyma03g01500
Glyma12g04400 Glyma07g30290 Glyma17g19940 Glyma05g30530 Glyma14g04890 Glyma09g38740
Glyma19g42070 Glyma03g01530 Glyma12g04810 Glyma07g31470 Glyma17g22160 Glyma05g30770
Glyma14g06170 Glyma09g39150 Glyma19g43390 Glyma03g03050 Glyma12g05510 Glyma07g31570
Glyma17g22200 Glyma05g32100 Glyma14g07480 Glyma09g40820 Glyma19g43870 Glyma03g03120
Glyma12g08330 Glyma07g38330 Glyma17g31990 Glyma05g32800 Glyma14g07750 Glyma10g04380
Glyma19g44630 Glyma03g03780 Glyma12g08840 Glyma07g38840 Glyma17g35700 Glyma05g33560
Glyma14g08690 Glyma10g04390 Glyma20g08690 Glyma03g12460 Glyma12g09250 Glyma08g00450
Glyma17g35890 Glyma05g34320 Glyma14g09300 Glyma10g04430 Glyma20g11330 Glyma03g25740

Glyma12g10270 Glyma08g02930 Glyma17g36440 Glyma05g35010 Glyma14g09450 Glyma10g04480 Glyma20g16170 Glyma03g32140 Glyma12g13410 Glyma08g03690 Glyma17g37220 Glyma05g35260 Glyma14g12870 Glyma10g05260 Glyma20g18440 Glyma03g32170 Glyma12g19520 Glyma08g04110 Glyma17g38230 Glyma05g35610 Glyma14g13080 Glyma10g05870 Glyma20g19000 Glyma03g32410 Glyma12g22240 Glyma08g04460 Glyma18g02300 Glyma05g36630 Glyma14g13970 Glyma10g08040 Glyma20g22320 Glyma03g32710 Glyma12g22340 Glyma08g04700 Glyma18g04730 Glyma06g00230 Glyma14g14400 Glyma10g16010 Glyma20g27000 Glyma03g34830 Glyma12g28910 Glyma08g05350 Glyma18g04850 Glyma06g01120 Glyma14g24410 Glyma10g19040 Glyma20g31760 Glyma03g37190 Glyma12g29120 Glyma08g06950 Glyma18g07920 Glyma06g01510 Glyma14g24870 Glyma10g21320 Glyma20g32070 Glyma03g37470 Glyma12g32850 Glyma08g09180 Glyma18g07950 Glyma06g01700 Glyma14g25220 Glyma10g24350 Glyma20g35090 Glyma03g38340 Glyma12g33860 Glyma08g11800 Glyma18g08220 Glyma06g03910 Glyma14g25810 Glyma10g24620 Glyma20g35760 Glyma03g38830 Glyma12g36290 Glyma08g13370 Glyma18g11380 Glyma06g05410 Glyma14g32500 Glyma10g24760 Glyma20g35990 Glyma03g38850 Glyma13g03480 Glyma08g13680 Glyma18g12720 Glyma06g05650 Glyma14g32920 Glyma10g28520 Glyma20g36170 Glyma03g39460 Glyma13g04940 Glyma08g13960 Glyma18g14600 Glyma06g05660 Glyma14g35990 Glyma10g29580 Glyma20g36330 Glyma06g06660 Glyma14g36610 Glyma10g31190 Glyma20g37730 Glyma10g31340 Glyma20g38530

## Module 74

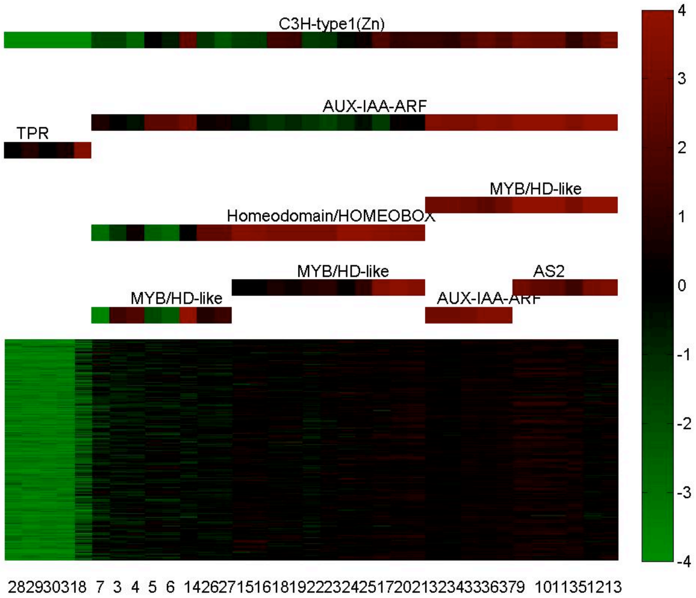


| GO_ACC | GO name | P value |
|---|---|---|
| GO:0042742 | P:defense response to bacterium | 0.06 |
| GO:0007067 | P:mitosis | 0.01 |
| GO:0007165 | P:signal transduction | 0.05 |
| GO:0008380 | P:RNA splicing | 0.00000511 |

| | | |
|---|---|---|
| GO:0007049 | P:cell cycle | 0.04 |
| GO:0016192 | P:vesicle-mediated transport | 0.0045605 |
| GO:0008219 | P:cell death | 0.0019143 |
| GO:0051028 | P:mRNA transport | 0.0000854 |
| GO:0051301 | P:cell division | 0.0000214 |
| GO:0055085 | P:transmembrane transport | 0.0019695 |
| GO:0015031 | P:protein transport | 0.02 |
| GO:0006364 | P:rRNA processing | 0.00000179 |
| GO:0009826 | P:unidimensional cell growth | 0.001417 |
| GO:0006886 | P:intracellular protein transport | 0.0000451 |
| GO:0000184 | P:nuclear-transcribed mRNA catabolic process,... | 3.35E-08 |
| GO:0006281 | P:DNA repair | 0.01 |
| GO:0006397 | P:mRNA processing | 1.47E-11 |
| GO:0006417 | P:regulation of translation | 0.00013946 |
| GO:0006470 | P:protein amino acid dephosphorylation | 0.0000124 |
| GO:0006511 | P:ubiquitin-dependent protein catabolic process | 0.0049194 |
| GO:0006813 | P:potassium ion transport | 0.0016416 |
| GO:0006814 | P:sodium ion transport | 0.0059492 |
| GO:0006816 | P:calcium ion transport | 0.00092005 |
| GO:0000398 | P:nuclear mRNA splicing, via spliceosome | 0.00000455 |

| TF | Family | Binding site | Gene number |
|---|---|---|---|
| Glyma02g38260 | AUX-IAA-ARF | | |
| Glyma03g00980 | MYB/HD-like | 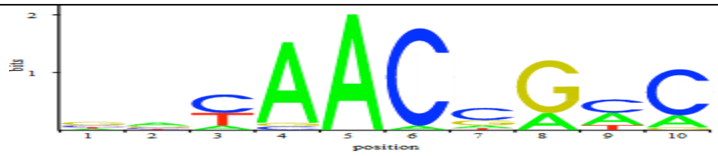 | 52 |
| Glyma07g15850 | MYB/HD-like | 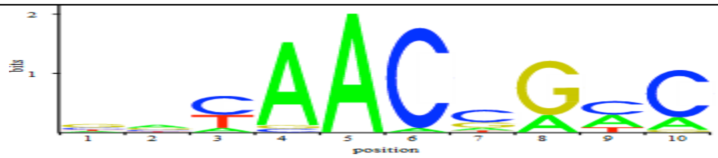 | 52 |
| Glyma08g03540 | C3H-type1(Zn) | | |
| Glyma11g02450 | Homeodomain/HOMEOBOX | | |

| | | | |
|---|---|---|---|
| Glyma13g43050 | AUX-IAA-ARF | | |
| Glyma18g05050 | MYB/HD-like | 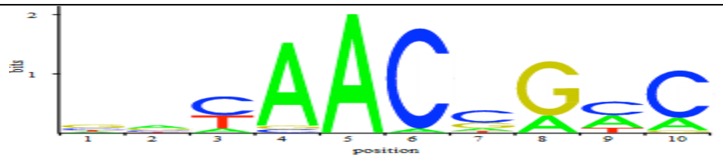 | 52 |
| Glyma20g31780 | AS2 | | |
| Glyma12g31490 | TPR | | |

Glyma0060s00360 Glyma10g31660 Glyma06g14500 Glyma15g13210 Glyma03g42370
Glyma13g04440 Glyma08g10340 Glyma18g02420 Glyma01g07800 Glyma10g32310 Glyma06g17280
Glyma15g13700 Glyma04g00370 Glyma13g04790 Glyma08g12240 Glyma18g05480 Glyma01g10320
Glyma10g35490 Glyma06g17780 Glyma15g15530 Glyma04g02570 Glyma13g05310 Glyma08g14040
Glyma18g14390 Glyma01g16390 Glyma10g36940 Glyma06g36900 Glyma15g15930 Glyma04g07180
Glyma13g07570 Glyma08g14830 Glyma18g14400 Glyma01g27030 Glyma10g39780 Glyma06g37030
Glyma15g15960 Glyma04g07960 Glyma13g18490 Glyma08g18570 Glyma18g17350 Glyma01g27240
Glyma10g41050 Glyma06g37170 Glyma15g18790 Glyma04g08930 Glyma13g19490 Glyma08g18720
Glyma18g36750 Glyma01g33750 Glyma10g42050 Glyma06g37270 Glyma15g21990 Glyma04g10580
Glyma13g19870 Glyma08g20050 Glyma18g36930 Glyma01g34420 Glyma10g42510 Glyma06g37280
Glyma15g23180 Glyma04g12890 Glyma13g21500 Glyma08g20670 Glyma18g45360 Glyma01g34630
Glyma10g42810 Glyma06g38840 Glyma15g35760 Glyma04g14500 Glyma13g22760 Glyma08g22210
Glyma18g45480 Glyma01g34780 Glyma10g43470 Glyma06g38910 Glyma15g35870 Glyma04g14530
Glyma13g24750 Glyma08g22950 Glyma18g47220 Glyma01g34820 Glyma11g01030 Glyma06g38960
Glyma15g36170 Glyma04g16890 Glyma13g25110 Glyma08g23370 Glyma18g47760 Glyma01g35360
Glyma11g01330 Glyma06g38990 Glyma15g39350 Glyma04g25800 Glyma13g25690 Glyma08g23680
Glyma18g48020 Glyma01g37160 Glyma11g02560 Glyma06g39060 Glyma15g39810 Glyma04g32140
Glyma13g25860 Glyma08g23900 Glyma18g52300 Glyma01g37400 Glyma11g02830 Glyma06g39110
Glyma15g40170 Glyma04g34830 Glyma13g28720 Glyma08g34280 Glyma18g52990 Glyma01g38640
Glyma11g06650 Glyma06g39170 Glyma15g40380 Glyma04g34850 Glyma13g29420 Glyma08g36060
Glyma18g53610 Glyma01g42630 Glyma11g07000 Glyma06g39240 Glyma16g00310 Glyma04g37290
Glyma13g30200 Glyma08g41670 Glyma18g53880 Glyma01g42920 Glyma11g07890 Glyma06g39300
Glyma16g00900 Glyma04g40290 Glyma13g31020 Glyma08g41710 Glyma1902s00200
Glyma01g43260 Glyma11g08020 Glyma06g39310 Glyma16g01100 Glyma04g40740 Glyma13g31550
Glyma08g46830 Glyma19g00530 Glyma01g43860 Glyma11g08110 Glyma06g43650 Glyma16g01470
Glyma04g40900 Glyma13g32440 Glyma08g46900 Glyma19g00550 Glyma01g44500 Glyma11g08760
Glyma06g46630 Glyma16g01840 Glyma04g41610 Glyma13g33280 Glyma08g47600 Glyma19g01490

Glyma02g01920 Glyma11g09350 Glyma06g46650 Glyma16g02310 Glyma04g43080 Glyma13g33600
Glyma08g47630 Glyma19g01500 Glyma02g01930 Glyma11g09980 Glyma06g47850 Glyma16g05670
Glyma04g43440 Glyma13g34300 Glyma08g47900 Glyma19g01530 Glyma02g05520 Glyma11g10390
Glyma07g00520 Glyma16g06170 Glyma05g01740 Glyma13g35740 Glyma09g02290 Glyma19g01920

Glyma02g08540 Glyma11g10860 Glyma07g01260 Glyma16g06700 Glyma05g02180 Glyma13g36820
Glyma09g02830 Glyma19g02540 Glyma02g09030 Glyma11g13680 Glyma07g02340 Glyma16g07600
Glyma05g02540 Glyma13g36920 Glyma09g04480 Glyma19g03150 Glyma02g09990 Glyma11g13750
Glyma07g02640 Glyma16g08320 Glyma05g02590 Glyma13g37030 Glyma09g04910 Glyma19g10120
Glyma02g13280 Glyma11g14050 Glyma07g03160 Glyma16g08590 Glyma05g03480 Glyma13g37650
Glyma09g04930 Glyma19g18830 Glyma02g16550 Glyma11g14450 Glyma07g03680 Glyma16g15810
Glyma05g05010 Glyma13g40930 Glyma09g06720 Glyma19g24620 Glyma02g19410 Glyma11g15040
Glyma07g03820 Glyma16g18450 Glyma05g08160 Glyma13g41820 Glyma09g07570 Glyma19g32500
Glyma02g26620 Glyma11g16210 Glyma07g04190 Glyma16g20260 Glyma05g08200 Glyma13g42300
Glyma09g09720 Glyma19g33430 Glyma02g29190 Glyma11g19670 Glyma07g04520 Glyma16g21560
Glyma05g09040 Glyma13g44430 Glyma09g09770 Glyma19g33590 Glyma02g29990 Glyma11g31810
Glyma07g04900 Glyma16g23960 Glyma05g10140 Glyma14g01390 Glyma09g09940 Glyma19g35430
Glyma02g38500 Glyma11g32270 Glyma07g05290 Glyma16g28200 Glyma05g10610 Glyma14g01600
Glyma09g10550 Glyma19g36330 Glyma02g40890 Glyma11g36020 Glyma07g05720 Glyma16g32990
Glyma05g21230 Glyma14g02700 Glyma09g15130 Glyma19g37000 Glyma02g42220 Glyma11g37750
Glyma07g05940 Glyma16g33890 Glyma05g24430 Glyma14g02960 Glyma09g15980 Glyma19g39210
Glyma02g43770 Glyma11g37770 Glyma07g08320 Glyma17g00710 Glyma05g26620 Glyma14g03320
Glyma09g28120 Glyma19g39420 Glyma02g44250 Glyma12g01430 Glyma07g11670 Glyma17g00740
Glyma05g26750 Glyma14g04480 Glyma09g28170 Glyma19g40130 Glyma02g44330 Glyma12g02230
Glyma07g11880 Glyma17g02640 Glyma05g27360 Glyma14g04520 Glyma09g29480 Glyma19g40600
Glyma02g45820 Glyma12g02320 Glyma07g17890 Glyma17g03120 Glyma05g27430 Glyma14g05140
Glyma09g30440 Glyma19g41280 Glyma02g46040 Glyma12g02680 Glyma07g28990 Glyma17g04180
Glyma05g29100 Glyma14g06680 Glyma09g32290 Glyma19g42410 Glyma02g46350 Glyma12g02950
Glyma07g30910 Glyma17g06310 Glyma05g30830 Glyma14g07600 Glyma09g32640 Glyma19g43160
Glyma02g47150 Glyma12g03140 Glyma07g31030 Glyma17g09270 Glyma05g31610 Glyma14g07790
Glyma09g32660 Glyma19g43990 Glyma02g47360 Glyma12g05740 Glyma07g31350 Glyma17g09320
Glyma05g32480 Glyma14g09460 Glyma09g32900 Glyma19g45140 Glyma03g01850 Glyma12g06020
Glyma07g31850 Glyma17g09750 Glyma05g32640 Glyma14g12570 Glyma09g35900 Glyma20g01360
Glyma03g04500 Glyma12g06440 Glyma07g36390 Glyma17g10150 Glyma05g32890 Glyma14g15170
Glyma09g38310 Glyma20g10260 Glyma03g06700 Glyma12g07010 Glyma07g37530 Glyma17g10780
Glyma05g34120 Glyma14g21210 Glyma09g38560 Glyma20g11980 Glyma03g14700 Glyma12g08830
Glyma07g40060 Glyma17g11800 Glyma05g34280 Glyma14g23650 Glyma09g38800 Glyma20g18440
Glyma03g23760 Glyma12g13060 Glyma07g40090 Glyma17g12070 Glyma05g34580 Glyma14g35280
Glyma09g39120 Glyma20g19970 Glyma03g24510 Glyma12g14310 Glyma0859s00200
Glyma17g12730 Glyma05g36490 Glyma14g36580 Glyma09g40400 Glyma20g23310 Glyma03g27290
Glyma12g28640 Glyma08g00300 Glyma17g12770 Glyma05g37240 Glyma1555s00200Glyma09g40480
Glyma20g24190 Glyma03g27730 Glyma12g29170 Glyma08g00510 Glyma17g14020 Glyma05g37370

Glyma15g00840 Glyma10g02040 Glyma20g26220 Glyma03g30510 Glyma12g31620 Glyma08g02180 Glyma17g14040 Glyma06g00440 Glyma15g03070 Glyma10g03290 Glyma20g27950 Glyma03g32680 Glyma12g32820 Glyma08g02310 Glyma17g15400 Glyma06g02610 Glyma15g03600 Glyma10g03700 Glyma20g30660 Glyma03g33620 Glyma12g33400 Glyma08g03080 Glyma17g23450 Glyma06g04160 Glyma15g04060 Glyma10g05520 Glyma20g32030 Glyma03g36030 Glyma12g33540 Glyma08g05080 Glyma17g31280 Glyma06g07280 Glyma15g06710 Glyma10g07630 Glyma20g33390 Glyma03g36550 Glyma12g33670 Glyma08g05390 Glyma17g33510 Glyma06g08010 Glyma15g06890 Glyma10g24460 Glyma20g35300 Glyma03g36770 Glyma12g34840 Glyma08g05570 Glyma17g34910 Glyma06g09020 Glyma15g07760 Glyma10g25620 Glyma20g35940 Glyma03g37510 Glyma13g00230 Glyma08g06280 Glyma17g35690 Glyma06g11630 Glyma15g08000 Glyma10g28030 Glyma20g36190 Glyma03g37980 Glyma13g03270 Glyma08g06390 Glyma17g37190 Glyma06g11640 Glyma15g08350 Glyma10g29080 Glyma20g36510 Glyma03g39850 Glyma13g03760 Glyma08g07680 Glyma18g01160 Glyma06g13200 Glyma15g08940 Glyma10g29610 Glyma20g37690 Glyma03g40500 Glyma13g03930 Glyma08g09630 Glyma18g01680 Glyma06g13930 Glyma15g09630 Glyma10g30970 Glyma20g38250 Glyma03g41400 Glyma13g04420 Glyma08g09740 Glyma18g01710 Glyma06g14060 Glyma15g10370 Glyma10g31320 Glyma20g39260

# Module 76

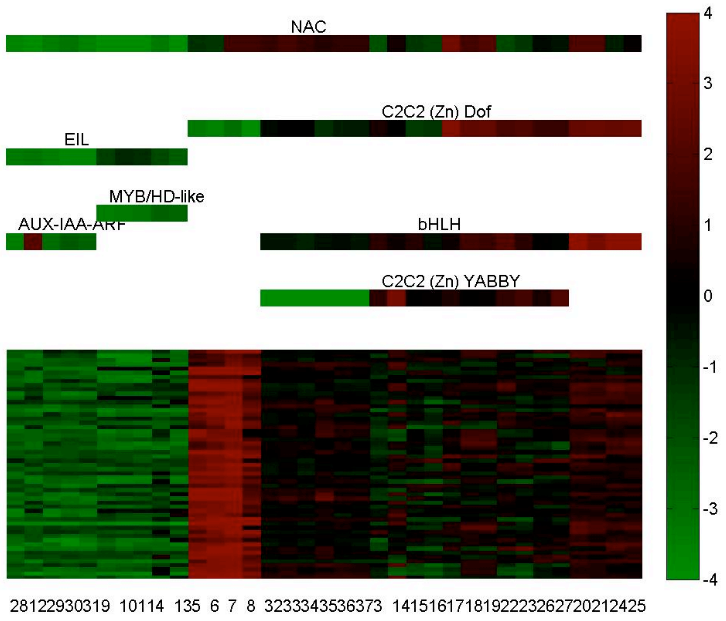


| GO_ACC | GO name | P value |
|---|---|---|
| GO:0009611 | P:response to wounding | 0.0014988 |
| GO:0006810 | P:transport | 0.03 |
| GO:0008152 | P:metabolic process | 0.0000355 |

| | | |
|---|---|---|
| GO:0006952 | P:defense response | 0.02 |

| TF | Family | Binding site | Gene number |
|---|---|---|---|
| Glyma02 g12220 | NAC | | |
| Glyma02 g38260 | AUX-IAA-ARF | | |
| Glyma13 g03700 | EIL | | |
| Glyma13 g22620 | C2C2 (Zn) YABBY | 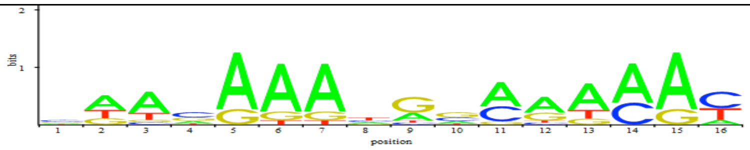 | 21 |
| Glyma15 g18580 | bHLH | | |
| Glyma17 g10920 | C2C2 (Zn) Dof | 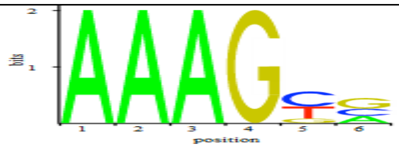 | 7 |
| Glyma12 g10790 | MYB/HD-like | 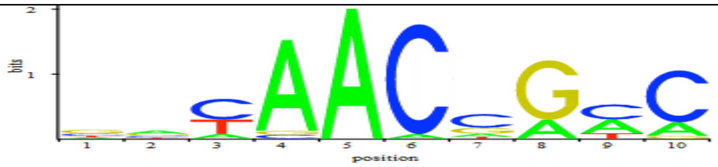 | 7 |

Glyma0023s00410 Glyma09g25470 Glyma05g32850 Glyma15g13560 Glyma02g09850
Glyma12g05320 Glyma07g36160 Glyma17g36360 Glyma01g02120 Glyma09g27780 Glyma05g37690
Glyma15g18640 Glyma03g05010 Glyma12g07770 Glyma07g37280 Glyma18g06360 Glyma01g32040
Glyma09g27850 Glyma06g05590 Glyma15g42260 Glyma03g16600 Glyma12g08870 Glyma07g39630
Glyma18g53070 Glyma01g32070 Glyma09g33820 Glyma06g09220 Glyma16g17680 Glyma03g26900
Glyma12g30430 Glyma08g01900 Glyma19g04390 Glyma01g42420 Glyma10g29750 Glyma06g19890
Glyma16g26100 Glyma03g28490 Glyma13g09240 Glyma08g07900 Glyma19g05380 Glyma01g42430
Glyma10g42820 Glyma06g47750 Glyma16g32560 Glyma04g05320 Glyma13g43870 Glyma08g16300
Glyma19g05880 Glyma01g44270 Glyma11g00650 Glyma06g47760 Glyma17g01170 Glyma04g05570

| | | | | | |
|---|---|---|---|---|---|
| Glyma14g08810 | Glyma08g16810 | Glyma19g09320 | Glyma01g44600 | Glyma11g00710 | Glyma07g02180 |
| Glyma17g03330 | Glyma04g09110 | Glyma14g26700 | Glyma08g21840 | Glyma19g26690 | Glyma01g44930 |
| Glyma11g01240 | Glyma07g14510 | Glyma17g04350 | Glyma05g07730 | Glyma15g01470 | Glyma08g23380 |
| Glyma20g24180 | Glyma01g45020 | Glyma11g15700 | Glyma07g14530 | Glyma17g05490 | Glyma05g24740 |
| Glyma15g01490 | Glyma09g00450 | Glyma20g37560 | Glyma02g07110 | Glyma11g29720 | Glyma07g30880 |
| Glyma17g13280 | Glyma09g07470 | | | | |

# Module 77

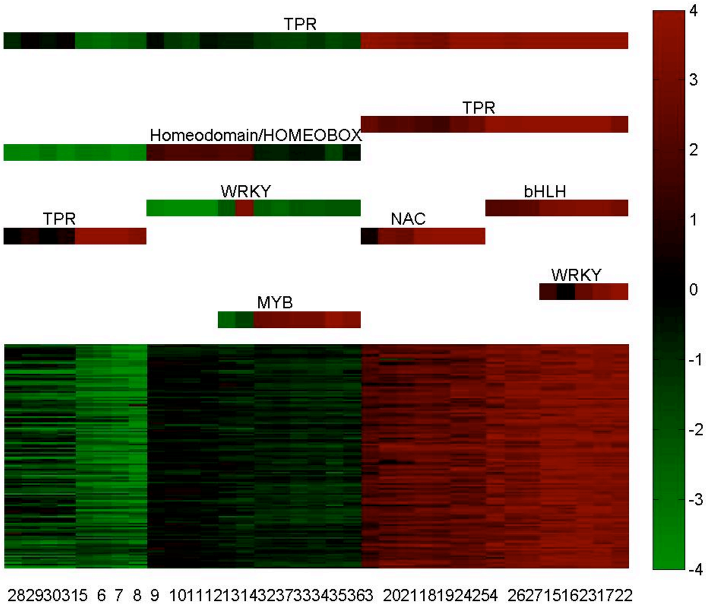


| GO_ACC | GO name | P value |
|---|---|---|
| GO:0022900 | P:electron transport chain | 0.00011682 |
| GO:0055114 | P:oxidation reduction | 0.0004343 |
| GO:0019253 | P:reductive pentose-phosphate cycle | 1.88E-08 |
| GO:0015986 | P:ATP synthesis coupled proton transport | 0.0005467 |
| GO:0015979 | P:photosynthesis | 1.66E-11 |
| GO:0007165 | P:signal transduction | 0.02 |
| GO:0006783 | P:heme biosynthetic process | 0.0000206 |
| GO:0006414 | P:translational elongation | 0.0030806 |
| GO:0006412 | P:translation | 0.01 |
| GO:0000160 | P:two-component signal transduction system (p... | 0.0039262 |
| GO:0006096 | P:glycolysis | 0.03 |
| GO:0006810 | P:transport | 0.00028574 |

| TF | Family |
|---|---|
| Glyma03g32740 | bHLH |
| Glyma04g24430 | TPR |
| Glyma06g15220 | WRKY |
| Glyma06g30000 | TPR |
| Glyma11g06640 | Homeodomain/HOMEOBOX |
| Glyma13g35560 | NAC |
| Glyma12g31490 | TPR |
| Glyma05g31800 | WRKY |
| Glyma20g35610 | MYB |

Glyma01g03260 Glyma09g38610 Glyma05g31720 Glyma15g39070 Glyma04g01610 Glyma12g01210 Glyma07g17180 Glyma18g40820 Glyma01g29630 Glyma10g06970 Glyma05g33750 Glyma15g43150 Glyma04g01700 Glyma12g03020 Glyma07g30360 Glyma18g41940 Glyma01g32250 Glyma10g09260 Glyma05g34130 Glyma16g01630 Glyma04g01750 Glyma12g05330 Glyma07g30430 Glyma18g47710 Glyma01g42000 Glyma10g11570 Glyma06g00320 Glyma16g02010 Glyma04g02100 Glyma12g05650 Glyma07g38390 Glyma18g48010 Glyma01g44000 Glyma10g11580 Glyma06g00380 Glyma16g02770 Glyma04g03820 Glyma12g06350 Glyma08g02800 Glyma18g52340 Glyma02g04320 Glyma10g17570 Glyma06g01690 Glyma16g03450 Glyma04g07090 Glyma12g06920 Glyma08g05560 Glyma19g01220 Glyma02g05350 Glyma10g22710 Glyma06g01850 Glyma16g15800 Glyma04g11180 Glyma12g30830 Glyma08g05970 Glyma19g01650 Glyma02g05490 Glyma10g26920 Glyma06g02200 Glyma16g19560 Glyma04g24430 Glyma12g34950 Glyma08g06820 Glyma19g06130 Glyma02g06830 Glyma10g29540 Glyma06g03920 Glyma16g23710 Glyma04g28020 Glyma13g00520 Glyma08g06890 Glyma19g06340 Glyma02g10540 Glyma10g36650 Glyma06g07180 Glyma16g23910 Glyma04g30670 Glyma13g04640 Glyma08g09160 Glyma19g06370 Glyma02g26150 Glyma10g38030 Glyma06g13640 Glyma17g04190 Glyma04g33480 Glyma13g07540 Glyma08g09480 Glyma19g27940 Glyma02g27130 Glyma10g38410 Glyma06g13880 Glyma17g06060 Glyma04g34330 Glyma13g07610 Glyma08g12920 Glyma19g32770 Glyma02g31480 Glyma10g38960 Glyma06g15200 Glyma17g06660 Glyma04g36250 Glyma13g23640 Glyma08g13160 Glyma19g33510 Glyma02g37860 Glyma10g40930 Glyma06g16700 Glyma17g09200 Glyma04g38360 Glyma13g23810 Glyma08g14280 Glyma19g33580 Glyma03g01720 Glyma10g43130 Glyma06g18640 Glyma17g12380 Glyma04g39670 Glyma13g27490 Glyma08g14970 Glyma19g36130

Glyma03g07560 Glyma10g43990 Glyma06g24470 Glyma17g13930 Glyma04g40970 Glyma13g33710 Glyma08g17110 Glyma19g38010 Glyma03g07570 Glyma10g44020 Glyma06g30000 Glyma17g14650

Glyma04g41210 Glyma13g35620 Glyma08g21900 Glyma19g41590 Glyma03g14630 Glyma10g44120 Glyma06g35690 Glyma17g17680 Glyma05g03340 Glyma13g39470 Glyma08g25610 Glyma20g09590 Glyma03g17360 Glyma11g01470 Glyma07g02240 Glyma17g20020 Glyma05g04210 Glyma13g41980 Glyma08g33520 Glyma20g21100 Glyma03g28240 Glyma11g03320 Glyma07g05490 Glyma17g29310 Glyma05g14730 Glyma13g44940 Glyma08g33560 Glyma20g23760 Glyma03g29870 Glyma11g03350 Glyma07g06160 Glyma17g34480 Glyma05g15660 Glyma14g10820 Glyma08g41620 Glyma20g28850 Glyma03g30610 Glyma11g10710 Glyma07g06860 Glyma17g34690 Glyma05g24260 Glyma14g10990 Glyma08g41660 Glyma20g29450 Glyma03g30730 Glyma11g13300 Glyma07g07040 Glyma18g00310 Glyma05g26230 Glyma14g13270 Glyma08g43950 Glyma20g30940 Glyma03g32740 Glyma11g14410 Glyma07g07120 Glyma18g03970 Glyma05g26510 Glyma14g27040 Glyma09g26040 Glyma20g37770 Glyma03g33400 Glyma11g14960 Glyma07g09820 Glyma18g08900 Glyma05g29800 Glyma15g03400 Glyma09g31980 Glyma20g38720 Glyma03g35380 Glyma11g18650 Glyma07g10060 Glyma18g14410 Glyma05g29820 Glyma15g11490 Glyma09g36120 Glyma20g38750 Glyma03g40510 Glyma11g21150 Glyma07g16230 Glyma18g14530 Glyma05g30040 Glyma15g24990 Glyma09g38340 Glyma20g38820 Glyma04g00270 Glyma11g34350 Glyma07g16250 Glyma18g40280 Glyma05g31070 Glyma15g32270

## Module 78

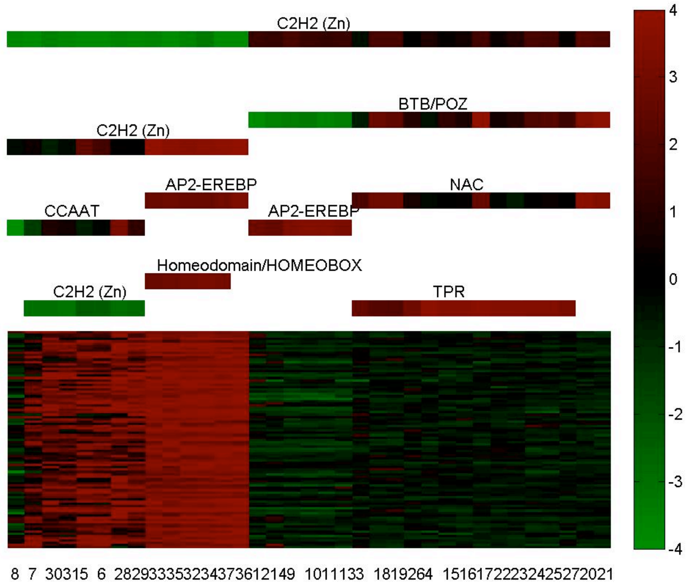


| GO_ACC | GO name | P value |
|---|---|---|
| GO:0007047 | P:cellular cell wall organization | 0.00098772 |
| GO:0042744 | P:hydrogen peroxide catabolic process | 0.02 |
| GO:0009877 | P:nodulation | 0.00014398 |
| GO:0009873 | P:ethylene mediated signaling pathway | 0.01 |
| GO:0006350 | P:transcription | 0.0035682 |

| | | |
|---|---|---|
| GO:0008360 | P:regulation of cell shape | 0.00060598 |
| GO:0006355 | P:regulation of transcription, DNA-dependent | 0.00016045 |
| GO:0009252 | P:peptidoglycan biosynthetic process | 0.00043081 |

| TF | Family | Binding site | Gene number |
|---|---|---|---|
| Glyma06 g30000 | TPR | | |
| Glyma07 g04050 | CCAAT | | |
| Glyma09 g04630 | AP2-EREBP | | |
| Glyma09 g33240 | AP2-EREBP | | |
| Glyma13 g35550 | NAC | | |
| Glyma13 g40240 | C2H2 (Zn) | 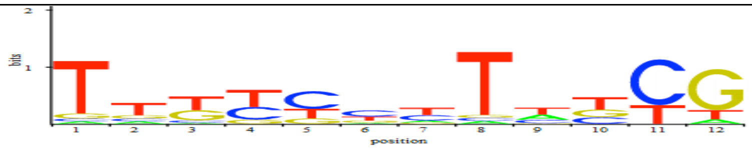 | 44 |
| Glyma14 g09310 | Homeodomain/HOMEOBOX | | |
| Glyma14 g13360 | C2H2 (Zn) | 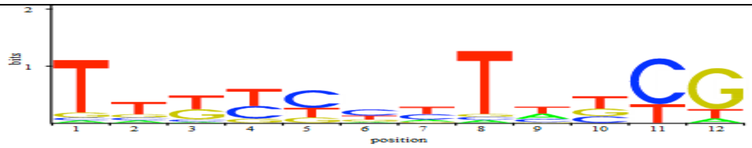 | 44 |
| Glyma18 g05720 | BTB/POZ | | |
| Glyma20 g00850 | C2H2 (Zn) | 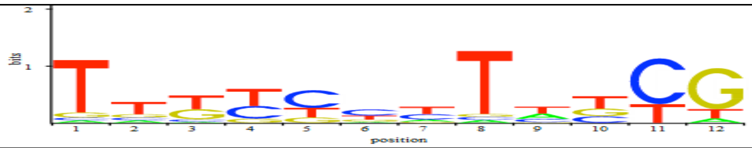 | 44 |

| | | | |
|---|---|---|---|

Glyma01g02460 Glyma11g01010 Glyma05g17430 Glyma16g04410 Glyma02g38990 Glyma13g25280
Glyma08g44760 Glyma18g47380 Glyma01g03450 Glyma11g06180 Glyma05g23630 Glyma16g05080
Glyma02g42730 Glyma13g27640 Glyma08g45320 Glyma18g51250 Glyma01g04330 Glyma11g12650
Glyma05g37230 Glyma16g08990 Glyma03g22840 Glyma13g30290 Glyma09g02590 Glyma19g04220
Glyma01g10100 Glyma11g19030 Glyma06g05090 Glyma16g24610 Glyma03g26790 Glyma13g33140
Glyma09g08270 Glyma19g07600 Glyma01g10150 Glyma11g20250 Glyma06g08340 Glyma16g34150
Glyma03g27470 Glyma13g34550 Glyma09g08550 Glyma19g29000 Glyma01g38430 Glyma11g20790
Glyma06g15690 Glyma17g00980 Glyma03g28450 Glyma13g36140 Glyma09g15550 Glyma19g30460
Glyma01g38440 Glyma11g29000 Glyma06g20810 Glyma17g01510 Glyma03g34170 Glyma13g39330
Glyma09g29580 Glyma19g31190 Glyma01g39460 Glyma11g31700 Glyma07g02680 Glyma17g01840
Glyma03g36000 Glyma13g41990 Glyma09g33510 Glyma19g36870 Glyma01g44530 Glyma11g35930
Glyma07g14410 Glyma17g14520 Glyma03g38160 Glyma14g36690 Glyma09g38920 Glyma19g38600
Glyma02g00560 Glyma11g36000 Glyma07g16080 Glyma17g16660 Glyma03g39040 Glyma14g37040
Glyma09g38930 Glyma19g40770 Glyma02g00640 Glyma12g09430 Glyma07g31200 Glyma18g02430
Glyma03g40970 Glyma15g01110 Glyma09g40210 Glyma19g40960 Glyma02g01020 Glyma12g30210
Glyma07g35780 Glyma18g02440 Glyma04g05010 Glyma15g03390 Glyma09g41190 Glyma19g41610
Glyma02g03360 Glyma12g30220 Glyma07g38900 Glyma18g02470 Glyma04g08250 Glyma15g07040
Glyma09g41490 Glyma19g43620 Glyma02g04180 Glyma12g30990 Glyma07g39230 Glyma18g05580
Glyma04g09570 Glyma15g08880 Glyma10g00850 Glyma20g04990 Glyma02g04190 Glyma12g34410
Glyma07g39800 Glyma18g06680 Glyma04g33640 Glyma15g13490 Glyma10g06190 Glyma20g22980
Glyma02g05930 Glyma12g35850 Glyma08g02330 Glyma18g35710 Glyma04g35380 Glyma15g19820
Glyma10g28820 Glyma20g27780 Glyma02g13810 Glyma13g20250 Glyma08g17530 Glyma18g44250
Glyma04g39240 Glyma15g20180 Glyma10g31020 Glyma20g29120 Glyma02g14160 Glyma13g20510
Glyma08g23320 Glyma18g44650 Glyma05g04040 Glyma15g41620 Glyma10g31350 Glyma20g34930
Glyma02g33000 Glyma13g24800 Glyma08g28220 Glyma18g47100 Glyma10g38630 Glyma20g36460
Glyma10g32670 Glyma20g36160

# Module 86

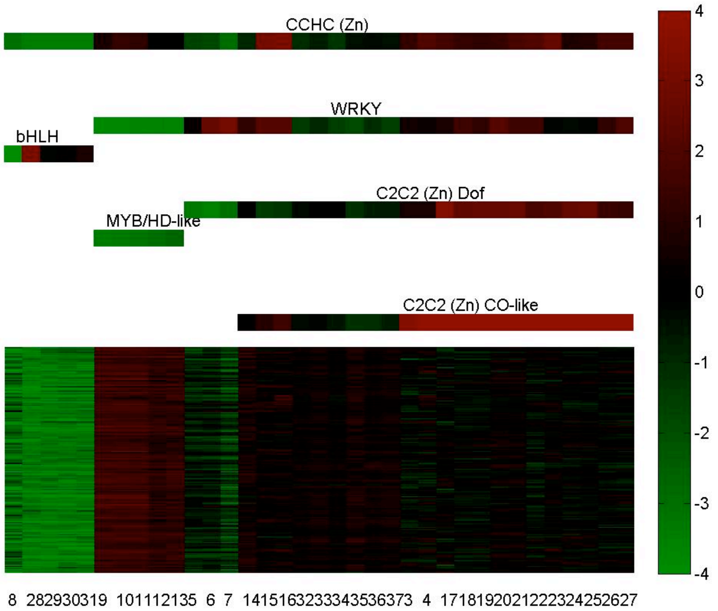


| GO_ACC | GO name | P value |
|---|---|---|
| GO:0043044 | P:ATP-dependent chromatin remodeling | 0.00000573 |
| GO:0045449 | P:regulation of transcription | 0.0000407 |
| GO:0042254 | P:ribosome biogenesis | 0.0000882 |
| GO:0016568 | P:chromatin modification | 0.0000288 |
| GO:0016332 | P:establishment or maintenance of polarity of... | 0.00015588 |
| GO:0008380 | P:RNA splicing | 0.000013 |
| GO:0007049 | P:cell cycle | 0.01 |
| GO:0006526 | P:arginine biosynthetic process | 0.00044438 |
| GO:0006457 | P:protein folding | 0.0014946 |
| GO:0006414 | P:translational elongation | 0.0077848 |
| GO:0006412 | P:translation | 0.0000155 |
| GO:0006397 | P:mRNA processing | 0.0000534 |
| GO:0006350 | P:transcription | 0.0010061 |
| GO:0006221 | P:pyrimidine nucleotide biosynthetic process | 0.0014347 |
| GO:0051260 | P:protein homooligomerization | 0.00062643 |
| GO:0006979 | P:response to oxidative stress | 0.02 |

| TF | Family | Binding site | Gene numbe |
|---|---|---|---|

| | | | r |
|---|---|---|---|
| Glyma03g25280 | bHLH | | |
| Glyma09g41050 | WRKY | | |
| Glyma17g10920 | C2C2 (Zn) Dof | | |
| Glyma18g51320 | C2C2 (Zn) CO-like | | |
| Glyma19g41150 | CCHC (Zn) | 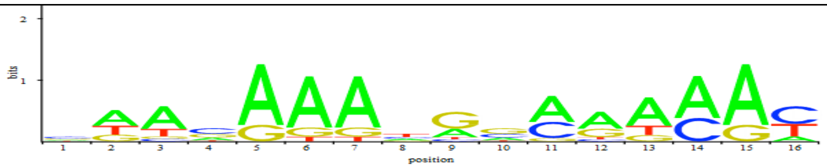 | 97 |
| Glyma12g10790 | MYB/HD-like | | |

Glyma01g01930 Glyma09g38430 Glyma05g34420 Glyma15g10370 Glyma03g27860 Glyma12g37130 Glyma07g37430 Glyma18g43380 Glyma01g03490 Glyma09g40260 Glyma05g35610 Glyma15g13130 Glyma03g29930 Glyma13g04140 Glyma07g38050 Glyma18g45750 Glyma01g04820 Glyma10g06560 Glyma05g36160 Glyma15g37200 Glyma03g30020 Glyma13g11700 Glyma07g38270 Glyma18g47910 Glyma01g24650 Glyma10g13810 Glyma05g36260 Glyma15g39920 Glyma03g30030 Glyma13g18530 Glyma07g38520 Glyma18g52420 Glyma01g27200 Glyma10g15910 Glyma05g37900 Glyma15g40700 Glyma03g30800 Glyma13g20760 Glyma08g01500 Glyma18g52430 Glyma01g29950 Glyma10g24060 Glyma06g03270 Glyma16g00880 Glyma03g33080 Glyma13g23700 Glyma08g01700 Glyma19g22730 Glyma01g34940 Glyma10g26870 Glyma06g03340 Glyma16g04160 Glyma03g34880 Glyma13g24880 Glyma08g02140 Glyma19g24590 Glyma01g37270 Glyma10g28200 Glyma06g05380 Glyma16g05220 Glyma03g35080 Glyma13g25960 Glyma08g03390 Glyma19g26870 Glyma01g37280 Glyma10g29080 Glyma06g07890 Glyma16g05600 Glyma03g35620 Glyma13g26270 Glyma08g03480 Glyma19g27670 Glyma01g38120 Glyma10g30870 Glyma06g08680 Glyma16g06710 Glyma03g38720 Glyma13g27240 Glyma08g05250 Glyma19g29230 Glyma01g38450 Glyma10g31980 Glyma06g09160 Glyma16g22290 Glyma03g40260 Glyma13g27250 Glyma08g05260 Glyma19g30210 Glyma01g41810 Glyma10g39580 Glyma06g10280 Glyma16g27570 Glyma03g41270 Glyma13g28720 Glyma08g05320 Glyma19g30680 Glyma01g43480 Glyma10g40140 Glyma06g10680 Glyma16g28550 Glyma03g41760 Glyma13g35400 Glyma08g06950 Glyma19g32830 Glyma01g44380 Glyma10g43190 Glyma06g14280 Glyma16g30190 Glyma04g03210 Glyma13g39500 Glyma08g08590 Glyma19g32910 Glyma01g44970 Glyma10g44310

Glyma06g15510 Glyma17g02200 Glyma04g03260 Glyma13g41120 Glyma08g10540 Glyma19g33650 Glyma02g02690 Glyma11g00660 Glyma06g16220 Glyma17g02470 Glyma04g06280 Glyma13g42030 Glyma08g10910 Glyma19g34290 Glyma02g04150 Glyma11g01140 Glyma06g17470 Glyma17g02540 Glyma04g07820 Glyma13g42230 Glyma08g10980 Glyma19g37780 Glyma02g08440 Glyma11g02020 Glyma06g18070 Glyma17g02640 Glyma04g08570 Glyma13g44000 Glyma08g11680 Glyma19g38260 Glyma02g10470 Glyma11g04650 Glyma06g36160 Glyma17g03120 Glyma04g10330 Glyma13g44290 Glyma08g13330 Glyma19g41310 Glyma02g10480 Glyma11g07200 Glyma06g37500 Glyma17g03200 Glyma04g10850 Glyma13g44490 Glyma08g13970 Glyma19g42870 Glyma02g17520 Glyma11g08020 Glyma06g40790 Glyma17g04200 Glyma04g12630 Glyma14g00890 Glyma08g15400 Glyma19g43880 Glyma02g18640 Glyma11g08370 Glyma06g44980 Glyma17g04930 Glyma04g16810 Glyma14g02130 Glyma08g18300 Glyma20g01130 Glyma02g33350 Glyma11g14310 Glyma06g44990 Glyma17g07970 Glyma04g36910 Glyma14g02940 Glyma08g18920 Glyma20g02360 Glyma02g36750 Glyma11g15160 Glyma06g47340 Glyma17g08630 Glyma04g37620 Glyma14g04380 Glyma08g20090 Glyma20g07060 Glyma02g38500 Glyma11g15410 Glyma06g47970 Glyma17g11430 Glyma04g39390 Glyma14g04830 Glyma08g20150 Glyma20g07280 Glyma02g38540 Glyma11g16290 Glyma07g00490 Glyma17g12000 Glyma04g43650 Glyma14g04930 Glyma08g20290 Glyma20g08920 Glyma02g39540 Glyma11g18080 Glyma07g00940 Glyma17g12440 Glyma05g00400 Glyma14g05120 Glyma08g20730 Glyma20g11560 Glyma02g40350 Glyma11g21590 Glyma07g01330 Glyma17g14280 Glyma05g03770 Glyma14g05810 Glyma08g20940 Glyma20g11590 Glyma02g42680 Glyma11g28420 Glyma07g01520 Glyma17g14300 Glyma05g03790 Glyma14g11400 Glyma08g21720 Glyma20g17960 Glyma02g43800 Glyma11g33720 Glyma07g02040 Glyma17g15010 Glyma05g04580 Glyma14g13540 Glyma08g23120 Glyma20g21330 Glyma02g43970 Glyma11g35740 Glyma07g02050 Glyma17g24730 Glyma05g10960 Glyma14g16830 Glyma08g23930 Glyma20g22190 Glyma02g44440 Glyma12g01100 Glyma07g02990 Glyma17g29760 Glyma05g13110 Glyma14g27330 Glyma08g29130 Glyma20g23700 Glyma02g45830 Glyma12g01290 Glyma07g04170 Glyma17g33020 Glyma05g13330 Glyma14g33430 Glyma08g41440 Glyma20g28160 Glyma02g46510 Glyma12g01980 Glyma07g04230 Glyma17g37880 Glyma05g25610 Glyma14g36580 Glyma08g44800 Glyma20g29190 Glyma02g47150 Glyma12g06240 Glyma07g06670 Glyma18g02670 Glyma05g27940 Glyma14g36670 Glyma08g44870 Glyma20g35670 Glyma02g47740 Glyma12g10130 Glyma07g08910 Glyma18g04500 Glyma05g28010 Glyma14g37570 Glyma08g45190 Glyma20g36600 Glyma03g00600 Glyma12g12300 Glyma07g11180 Glyma18g06300 Glyma05g28620 Glyma14g40260 Glyma08g46420 Glyma20g38250 Glyma03g02250 Glyma12g29030 Glyma07g13320 Glyma18g06720 Glyma05g30170 Glyma15g00780 Glyma08g46440 Glyma20g39130 Glyma03g07750 Glyma12g29130 Glyma07g14770 Glyma18g08080 Glyma05g30780 Glyma15g00980 Glyma09g02230 Glyma09g33090 Glyma03g12130 Glyma12g30780 Glyma07g21160 Glyma18g08100 Glyma05g32110 Glyma15g03190 Glyma09g11970 Glyma09g33980 Glyma03g14740 Glyma12g35150 Glyma07g30290 Glyma18g14770 Glyma05g33890 Glyma15g03350 Glyma09g25250 Glyma09g35780 Glyma03g27260 Glyma12g36550 Glyma07g34610 Glyma18g35940 Glyma05g34330 Glyma15g03420 Glyma09g31060 Glyma09g36050 Glyma03g27270 Glyma12g36570 Glyma07g36030 Glyma18g38350 Glyma05g34400 Glyma15g06100 Glyma09g36760 Glyma09g36230

# Module 93

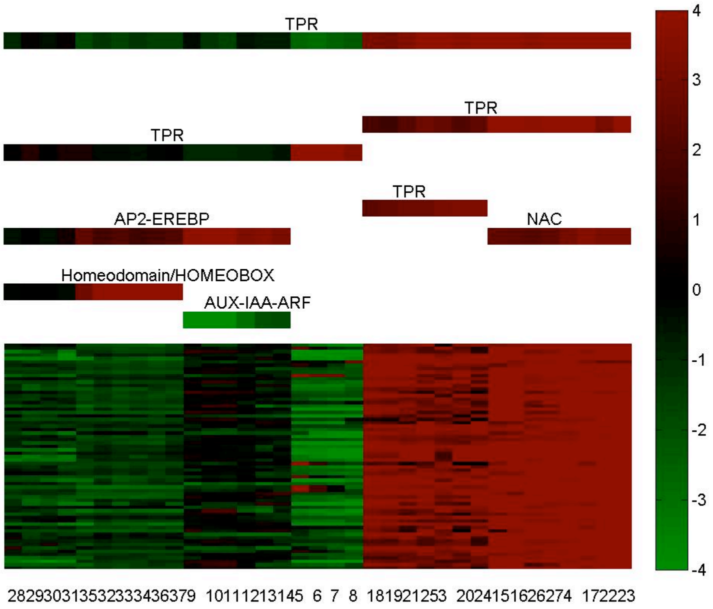


| GO_ACC | GO name | P value |
|---|---|---|
| GO:0007047 | P:cellular cell wall organization | 0.04 |
| GO:0055114 | P:oxidation reduction | 0.001759 |
| GO:0022900 | P:electron transport chain | 0.0063585 |
| GO:0018298 | P:protein-chromophore linkage | 0.00056279 |
| GO:0015979 | P:photosynthesis | 0.00000614 |
| GO:0009409 | P:response to cold | 0.0033912 |
| GO:0008360 | P:regulation of cell shape | 0.00021648 |
| GO:0006810 | P:transport | 0.0013866 |
| GO:0006457 | P:protein folding | 0.02 |
| GO:0009252 | P:peptidoglycan biosynthetic process | 0.00015329 |

| TF | Family |
|---|---|
| Glyma04g24430 | TPR |
| Glyma06g30000 | TPR |
| Glyma13g00950 | AP2-EREBP |
| Glyma14g10430 | Homeodomain/HOMEOBOX |
| Glyma15g02040 | AUX-IAA-ARF |

| | |
|---|---|
| Glyma16g02770 | TPR |
| Glyma17g00650 | NAC |
| Glyma12g31490 | TPR |

Glyma01g01370 Glyma10g01610 Glyma06g15890 Glyma16g04600 Glyma04g01750 Glyma11g36840 Glyma08g18750 Glyma18g50610 Glyma01g06110 Glyma10g24700 Glyma06g19560 Glyma17g03660 Glyma04g06970 Glyma12g36780 Glyma08g21340 Glyma19g03690 Glyma01g30350 Glyma10g27610 Glyma06g20990 Glyma17g31330 Glyma04g24430 Glyma13g37010 Glyma08g22840 Glyma19g06090 Glyma01g40810 Glyma10g40210 Glyma06g22220 Glyma17g38090 Glyma04g32360 Glyma14g04260 Glyma08g27420 Glyma19g28700 Glyma01g44150 Glyma10g42100 Glyma06g41650 Glyma18g00740 Glyma04g33290 Glyma14g04290 Glyma08g27450 Glyma19g41540 Glyma02g00240 Glyma10g42800 Glyma07g01680 Glyma18g06920 Glyma04g35220 Glyma14g05550 Glyma08g39520 Glyma19g45350 Glyma02g00440 Glyma10g43690 Glyma07g03260 Glyma18g07310 Glyma04g39090 Glyma14g13270 Glyma08g41460 Glyma20g02550 Glyma02g02300 Glyma11g01650 Glyma07g18150 Glyma18g14740 Glyma04g42870 Glyma14g15120 Glyma08g45550 Glyma20g23100 Glyma02g38280 Glyma11g04530 Glyma07g34740 Glyma18g19050 Glyma05g31510 Glyma14g36420 Glyma08g45560 Glyma20g24200 Glyma02g43440 Glyma11g25920 Glyma07g36900 Glyma18g36950 Glyma05g35150 Glyma14g39940 Glyma08g45590 Glyma20g24930 Glyma02g45170 Glyma11g26490 Glyma08g01890 Glyma18g40100 Glyma05g37700 Glyma15g19810 Glyma09g08260 Glyma20g27200 Glyma03g38930 Glyma11g27280 Glyma08g04570 Glyma18g43000 Glyma06g07060 Glyma15g40140 Glyma09g38490 Glyma20g36820 Glyma03g42240 Glyma11g36830 Glyma08g14740 Glyma18g47830 Glyma06g07070 Glyma15g43100 Glyma10g00310 Glyma15g43110 Glyma06g11890

## Module 94

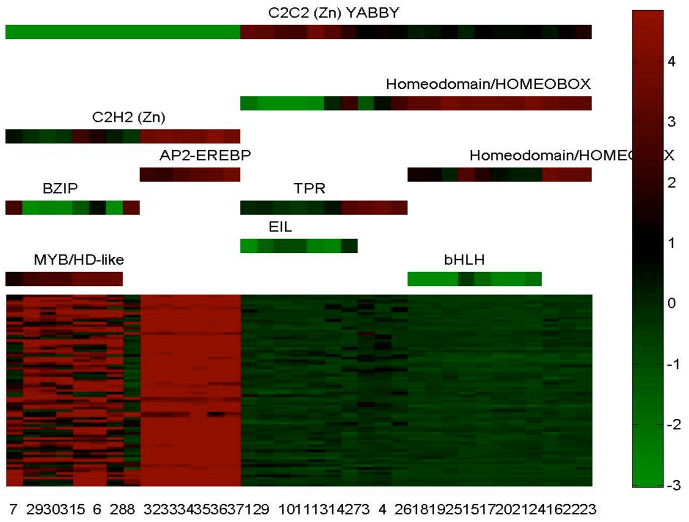

| GO_ACC | GO name | P value |
|---|---|---|
| GO:0006869 | P:lipid transport | 0.0000503 |
| GO:0042744 | P:hydrogen peroxide catabolic process | 0.000000733 |
| GO:0055114 | P:oxidation reduction | 7.06E-08 |
| GO:0006801 | P:superoxide metabolic process | 0.000058 |

| TF | Family |
|---|---|
| Glyma03g25280 | bHLH |
| Glyma03g38660 | MYB/HD-like |
| Glyma03g40730 | BZIP |
| Glyma11g02450 | Homeodomain/HOMEOBOX |
| Glyma13g03700 | EIL |
| Glyma13g22620 | C2C2 (Zn) YABBY |
| Glyma14g13360 | C2H2 (Zn) |
| Glyma16g02770 | TPR |
| Glyma19g29000 | AP2-EREBP |
| Glyma19g30810 | Homeodomain/HOMEOBOX |

Glyma01g04310 Glyma09g01680 Glyma05g04390 Glyma14g35190 Glyma02g15290 Glyma11g13770 Glyma07g04330 Glyma17g20560 Glyma01g04330 Glyma09g02610 Glyma05g21700 Glyma14g38170 Glyma02g39750 Glyma11g17550 Glyma07g04340 Glyma18g02690 Glyma01g17600 Glyma09g02680 Glyma05g26900 Glyma15g07700 Glyma02g40020 Glyma11g35700 Glyma07g04400 Glyma18g10500 Glyma01g25890 Glyma09g12200 Glyma05g26920 Glyma15g13510 Glyma03g17000 Glyma11g36000 Glyma07g31200 Glyma18g43040 Glyma01g34180 Glyma09g12380 Glyma05g30290 Glyma15g13550 Glyma03g17260 Glyma12g05770 Glyma07g33170 Glyma18g51880 Glyma01g37630 Glyma09g21010 Glyma06g01540 Glyma15g23980 Glyma03g38660 Glyma12g08260 Glyma08g09870 Glyma18g53300 Glyma01g45110 Glyma10g00950 Glyma06g04540 Glyma15g42000 Glyma03g42480 Glyma12g10850 Glyma08g09880 Glyma19g00840 Glyma02g00840 Glyma10g08120 Glyma06g05370 Glyma16g03710 Glyma04g04400 Glyma13g19520 Glyma08g13270 Glyma19g01120 Glyma02g02860 Glyma10g30110 Glyma06g38530 Glyma16g04750 Glyma04g05230 Glyma13g25280 Glyma08g17190 Glyma19g05260 Glyma02g03360 Glyma10g30120 Glyma06g43940 Glyma16g07830 Glyma04g10890 Glyma13g27110 Glyma08g24760 Glyma19g05800 Glyma02g07130 Glyma11g03810 Glyma06g45910 Glyma17g02260 Glyma04g12290 Glyma13g27440 Glyma08g24770 Glyma19g13540 Glyma02g07140 Glyma11g07670

Glyma06g45920 Glyma17g14850 Glyma04g16710 Glyma13g30330 Glyma08g28980 Glyma19g35270 Glyma02g07150 Glyma11g12810 Glyma06g48140 Glyma17g14880 Glyma04g40530 Glyma13g31690 Glyma08g48230 Glyma19g41250 Glyma05g00750 Glyma14g28740 Glyma19g45260

# Module 98

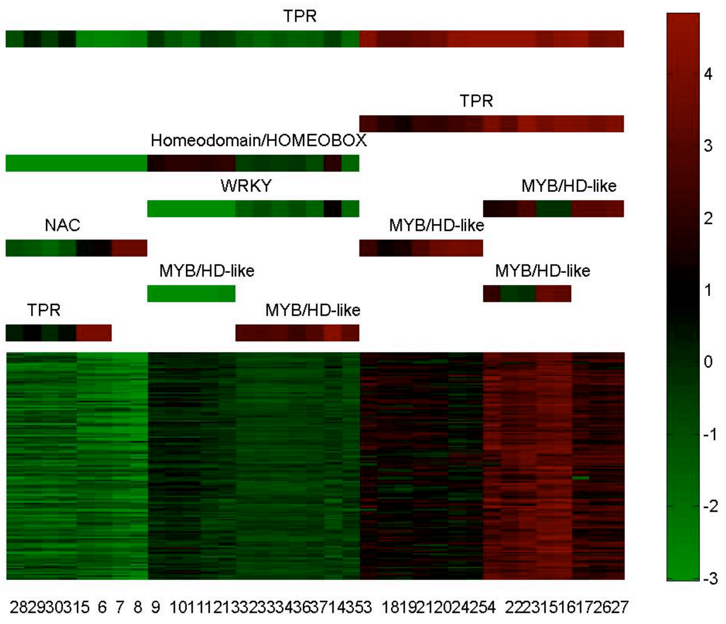


| GO_ACC | GO name | P value |
|---|---|---|
| GO:0015995 | P:chlorophyll biosynthetic process | 0.000038 |
| GO:0051301 | P:cell division | 0.06 |
| GO:0042254 | P:ribosome biogenesis | 0.00096845 |
| GO:0015996 | P:chlorophyll catabolic process | 0.0000293 |
| GO:0015979 | P:photosynthesis | 0.000000988 |
| GO:0010206 | P:photosystem II repair | 0.0000118 |
| GO:0007049 | P:cell cycle | 0.04 |
| GO:0006950 | P:response to stress | 0.0059723 |
| GO:0006508 | P:proteolysis | 0.0014759 |
| GO:0006457 | P:protein folding | 0.01 |
| GO:0006412 | P:translation | 1.21E-11 |
| GO:0030163 | P:protein catabolic process | 0.00025395 |

| TF | Family |
|---|---|
| Glyma03g00980 | MYB/HD-like |

| | |
|---|---|
| Glyma06g30000 | TPR |
| Glyma08g04670 | MYB/HD-like |
| Glyma09g41050 | WRKY |
| Glyma11g06640 | Homeodomain/HOMEOBOX |
| Glyma13g35550 | NAC |
| Glyma16g01980 | MYB/HD-like |
| Glyma17g13780 | MYB/HD-like |
| Glyma12g31490 | TPR |
| Glyma12g10790 | MYB/HD-like |
| Glyma04g24430 | TPR |

Glyma01g02150 Glyma10g43740 Glyma06g35690 Glyma15g01540 Glyma04g24960 Glyma13g00520 Glyma09g03340 Glyma18g00460 Glyma01g04090 Glyma11g01840 Glyma06g43060 Glyma15g05210 Glyma04g36420 Glyma13g04500 Glyma09g09000 Glyma18g00950 Glyma01g06470 Glyma11g05750 Glyma06g44820 Glyma15g05290 Glyma04g38360 Glyma13g05750 Glyma09g09190 Glyma18g02800 Glyma01g09310 Glyma11g06470 Glyma06g45440 Glyma15g05980 Glyma04g38480 Glyma13g07540 Glyma09g12580 Glyma18g05320 Glyma01g34700 Glyma11g10110 Glyma07g02580 Glyma15g06650 Glyma04g43250 Glyma13g10300 Glyma09g25500 Glyma18g07320 Glyma01g38940 Glyma11g12070 Glyma07g07880 Glyma15g06990 Glyma05g01410 Glyma13g11270 Glyma09g30460 Glyma18g12880 Glyma01g39200 Glyma11g13300 Glyma07g09430 Glyma15g08680 Glyma05g02800 Glyma13g11740 Glyma09g32370 Glyma18g47500 Glyma01g39500 Glyma11g14460 Glyma07g16890 Glyma15g24560 Glyma05g05450 Glyma13g12120 Glyma09g32600 Glyma19g01060 Glyma01g40920 Glyma11g14960 Glyma07g31830 Glyma15g32540 Glyma05g08580 Glyma13g19160 Glyma09g33790 Glyma19g01260 Glyma01g43630 Glyma11g18700 Glyma07g32550 Glyma15g40370 Glyma05g20200 Glyma13g20470 Glyma09g38820 Glyma19g01580 Glyma02g00780 Glyma11g19820 Glyma07g35510 Glyma15g42860 Glyma05g22730 Glyma13g20700 Glyma09g41960 Glyma19g03250 Glyma02g03660 Glyma11g26510 Glyma07g38300 Glyma16g04030 Glyma05g23860 Glyma13g20930 Glyma10g00570 Glyma19g03810 Glyma02g13790 Glyma11g28150 Glyma07g39720 Glyma16g04110 Glyma05g31670 Glyma13g20940 Glyma10g02660 Glyma19g06130 Glyma02g15670 Glyma11g31950 Glyma07g40340 Glyma16g04920 Glyma05g31720 Glyma13g21210 Glyma10g02950 Glyma19g25720 Glyma02g16830 Glyma11g35610 Glyma08g01060 Glyma16g25200 Glyma05g36670 Glyma13g22310 Glyma10g06160 Glyma19g28260 Glyma02g17130 Glyma11g36560 Glyma08g01520 Glyma16g27500 Glyma05g38010 Glyma13g23810 Glyma10g06500 Glyma19g29390 Glyma02g31480 Glyma11g37030 Glyma08g01570 Glyma16g31250 Glyma05g38050 Glyma13g23850 Glyma10g06740 Glyma19g30670 Glyma02g33070 Glyma12g02430

Glyma08g02870 Glyma17g00460 Glyma05g38570 Glyma13g24050 Glyma10g07290 Glyma19g36140 Glyma02g36820 Glyma12g04280 Glyma08g14920 Glyma17g01100 Glyma06g00380 Glyma13g24640 Glyma10g13190 Glyma19g37550 Glyma02g42300 Glyma12g05330 Glyma08g14970 Glyma17g02440 Glyma06g02200 Glyma13g28400 Glyma10g17570 Glyma19g39720 Glyma02g47060 Glyma12g06380 Glyma08g16210 Glyma17g07900 Glyma06g03140 Glyma13g30560 Glyma10g24540 Glyma19g44720 Glyma03g27850 Glyma12g06920 Glyma08g16370 Glyma17g08370 Glyma06g03510 Glyma13g32680 Glyma10g25710 Glyma20g03950 Glyma03g33410 Glyma12g08670 Glyma08g17110 Glyma17g10480 Glyma06g10160 Glyma13g39470 Glyma10g27990 Glyma20g13740 Glyma03g37100 Glyma12g09720 Glyma08g18580 Glyma17g13470 Glyma06g10490 Glyma13g43820 Glyma10g29800 Glyma20g18870 Glyma04g00330 Glyma12g11620 Glyma08g19000 Glyma17g15730 Glyma06g11450 Glyma13g44130 Glyma10g30990 Glyma20g20070 Glyma04g02100 Glyma12g16050 Glyma08g19730 Glyma17g16470 Glyma06g16570 Glyma14g01690 Glyma10g31000 Glyma20g24990 Glyma04g03100 Glyma12g26520 Glyma08g19810 Glyma17g17050 Glyma06g16700 Glyma14g06590 Glyma10g31740 Glyma20g33150 Glyma04g03420 Glyma12g30020 Glyma08g23440 Glyma17g21800 Glyma06g18470 Glyma14g17300 Glyma10g32060 Glyma20g35580 Glyma04g05890 Glyma12g30830 Glyma08g27150 Glyma17g29660 Glyma06g24430 Glyma14g26460 Glyma10g34420 Glyma20g35870 Glyma04g10170 Glyma12g32970 Glyma08g35830 Glyma17g29740 Glyma06g24470 Glyma14g39750 Glyma10g38950 Glyma20g37530 Glyma04g19020 Glyma12g36780 Glyma08g37360 Glyma17g38220 Glyma06g30020 Glyma15g01180 Glyma10g42030 Glyma20g38450

## Part D

**Figures 1 and 2 below illustrate how to determine the initial cluster number.**

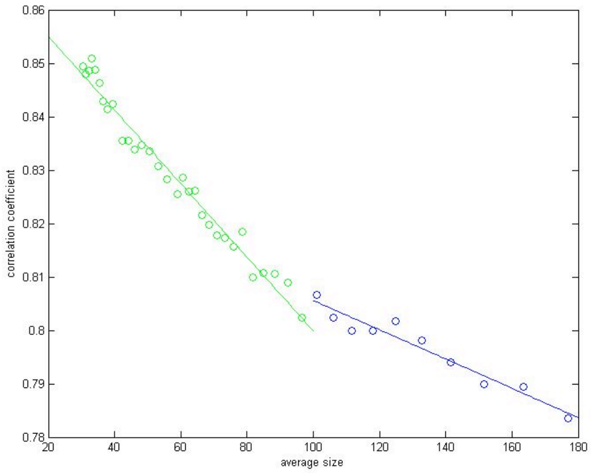


Figure 1

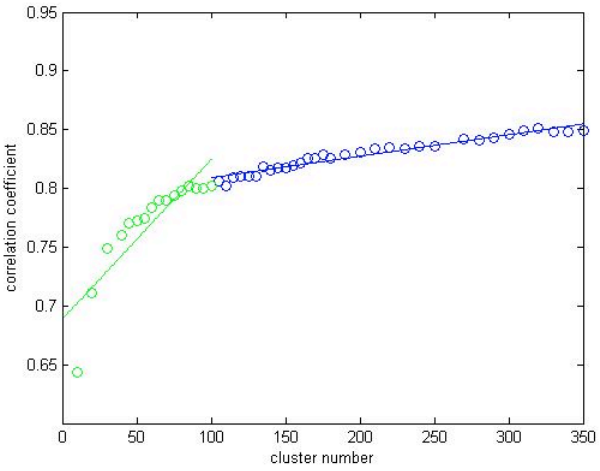


Figure 2
